# Genome diversity in the Neolithic Globular Amphorae culture and the spread of Indo-European languages

**DOI:** 10.1098/rspb.2017.1540

**Published:** 2017-11-22

**Authors:** Francesca Tassi, Stefania Vai, Silvia Ghirotto, Martina Lari, Alessandra Modi, Elena Pilli, Andrea Brunelli, Roberta Rosa Susca, Alicja Budnik, Damian Labuda, Federica Alberti, Carles Lalueza-Fox, David Reich, David Caramelli, Guido Barbujani

**Affiliations:** 1 Department of Life Sciences and Biotechnology, University of Firenze, Firenze, Italy; 2 Department of Biology, University of Firenze, Firenze, Italy; 3 Department of Human Biology, Cardinal Stefan Wyszyński University, Warsaw, Poland; 4 CHU Sainte-Justine Research Center, Department of Pediatrics, Université de Montréal, Montréal, PQ, Canada H3T 1C5; 5 Department of Evolutionary Biology, Institute for Biochemistry and Biology, Potsdam University, Potsdam, Germany; 6 Institute of Evolutionary Biology, University Pompeu Fabra, Barcelona, Spain; 7 Department of Genetics, Harvard Medical School, Boston, MA, USA; 8 Howard Hughes Medical Institute, Harvard Medical School, Boston, MA, USA

**Keywords:** population genomics, ancient DNA, migration, Neolithic, Indo-European, approximate Bayesian computation

## Abstract

It is unclear whether Indo-European languages in Europe spread from the Pontic steppes in the late Neolithic, or from Anatolia in the Early Neolithic. Under the former hypothesis, people of the Globular Amphorae culture (GAC) would be descended from Eastern ancestors, likely representing the Yamnaya culture. However, nuclear (six individuals typed for 597 573 SNPs) and mitochondrial (11 complete sequences) DNA from the GAC appear closer to those of earlier Neolithic groups than to the DNA of all other populations related to the Pontic steppe migration. Explicit comparisons of alternative demographic models via approximate Bayesian computation confirmed this pattern. These results are not in contrast to Late Neolithic gene flow from the Pontic steppes into Central Europe. However, they add nuance to this model, showing that the eastern affinities of the GAC in the archaeological record reflect cultural influences from other groups from the East, rather than the movement of people.

## 1. Introduction

Almost all Europeans speak Indo-European (IE) languages, and certainly not by chance. However, the place of origin of the first IE speakers has not been identified, nor has any consensus emerged about the time and the mechanisms by which IE languages spread over Western Eurasia. Based on the linguistic and archaeological evidence, the earliest speakers of a proto-IE language have often been identified with people living in the Pontic steppes about 6000 years ago [1], with their subsequent westward diffusion occurring in parallel with that of the Kurgan [2], or the Yamna [3] pastoral cultures. Conversely, genetic evidence of demic diffusion from the Near East into Europe [4] led Renfrew [5,6] to propose an earlier spread of Indo-European from Anatolia (9500–8000 years ago), through a single expansion carrying Neolithic technologies, genes and languages into much of Europe. In principle, language change does not need to be accompanied by migration, because cultural contacts, or a combination of cultural and demographic changes, may also lead to changes at the linguistic level. A common feature of the Kurgan and Anatolian models is that both postulate a migration fuelled by cultural innovations, horse riding or farming, respectively, which in turn facilitated language spread. This means that the spread of cultural novelty, documented by archaeological and linguistic evidence, has entailed demographic changes, which in principle have left their signature at the genomic level.

The majority of linguists currently support the hypothesis of a late, Pontic spread, here referred to as the Kurgan hypothesis [7], although Bayesian analyses of linguistic variation [8,9] seem to be easier to reconcile with an early diffusion of IE languages from Anatolia. However this inference is highly sensitive to prior assumptions, and when a different set of plausible prior assumptions is used, the same methods support the chronology suggested by the Kurgan hypothesis [10]. Recently, genetic data have provided strong new evidence relevant to this debate. There is ample genetic evidence that extensive migration accompanied the European spread of Neolithic technologies from the Near East [11–14]. Neolithic farmers came to occupy territories once inhabited by Mesolithic hunters and gatherers [15–20] which, for the sake of simplicity, we shall jointly consider here as hunter-gatherers. However, the hunter-gatherers did not go extinct, as data from Central Europe and Spain suggest a resurgence of a genomic component associated with them, during the Middle and late Neolithic [17,18,20]. There is now genetic evidence of population movements from the Russian steppes into Central Europe in the Bronze Age [20,21] and Iron Age [22]. These processes may or may not have had large-scale consequences at the demographic and linguistic level, but the later expansion would be consistent with a spread of languages associated with the Kurgan hypothesis [18,21].

In the great majority of ancient DNA studies, migration and admixture processes were not explicitly modelled, but instead inferred from levels of genetic resemblance among samples, as shown by principal component analysis (PCA) plots, clustering, and fitting of admixture graphs and clade tests based on *f*_3_- and *f*_4_-statistics (e.g. [18,20,21]). While giving a general overview of the data, these exploratory methods do not allow for formal comparison of alternative models, nor do they estimate parameters such as migration rates and population sizes. For that purpose, and to obtain insight into the origins of genomic variation in Middle Neolithic Central Europe, we collected and typed samples of 17 individuals from the Megalithic barrow of Kierzkowo (Poland), which is archaeologically assigned to the Globular Amphorae culture (GAC). The GAC is documented in Central and Eastern Europe, from the Elbe to the middle Dnieper, around 5400–4800 BP. It plays a crucial role in this debate because it has been argued to be associated with the first Indo-European migrations based on its burial rituals, including burial of livestock, usage of domestic horse, and presence of amber sun-disks [1]. Gimbutas [23] argued that when the Kurgan culture expanded from its homeland in the steppe and forest-steppe of Ukraine and South Russia, it did so in three waves, thus leading to the diffusion of the IE languages (see electronic supplementary material, figure S1). The GAC people are regarded by Gimbutas as part of the first wave, associated with the spread of the Yamna culture from the Pontic region to the Danube basin and the Balkans, between 5100 and 4900 BP [1]. If Gimbutas' theory is correct, the people of the GAC should have Yamna related admixture, as well as genetic affinity to the populations associated with the later, Bell Beaker culture, documented in many areas of Europe 4800 to 3800 BP.

## 2. Material and methods

### (a) Newly characterized ancient samples

The samples analysed in this study come from a Megalithic barrow, an elongated oval of almost 22 m in length and up to 6 m in breadth, in Kierzkowo, Żnin district, northwestern Poland [24], a typical example of the GAC burial rituals. The barrow was situated on top of a hill and contained in its western part a chamber about 10 m long, 1.5 m wide, made of stone slabs with a height of about 1 m, and divided in two unequal parts by a boulder. Inside the chamber, Neolithic human bones were gathered into two large clusters and a smaller one, mixed with animal bones, the latter bearing signs of dismemberment. Most of the skeletal material was fragmented and mixed, but human bones belonging to at least 23 different individuals were recovered. From 17 initial samples for which we had bone material, three were excluded because they were probably buried there much later as their position outside the burial chamber could have indicated; this was confirmed by radiocarbon dating (electronic supplementary material, table S1; see ‘Sample selection for population genetic analysis’ in the electronic supplementary material). After discarding samples with low DNA content, or which turned out to represent the same individual, we characterized 14 new mitochondrial genomes, 11 of them from the Neolithic period (see below). The nuclear genome data presented in this study are whole genome single nucleotide polymorphism (SNP) data on six individuals reported as part of a parallel broad-range study of ancient genetic variation of Eastern and Southeastern Europe [25].

### (b) Genomic data

#### (i) Population genetic reference data

**Figure 1.**
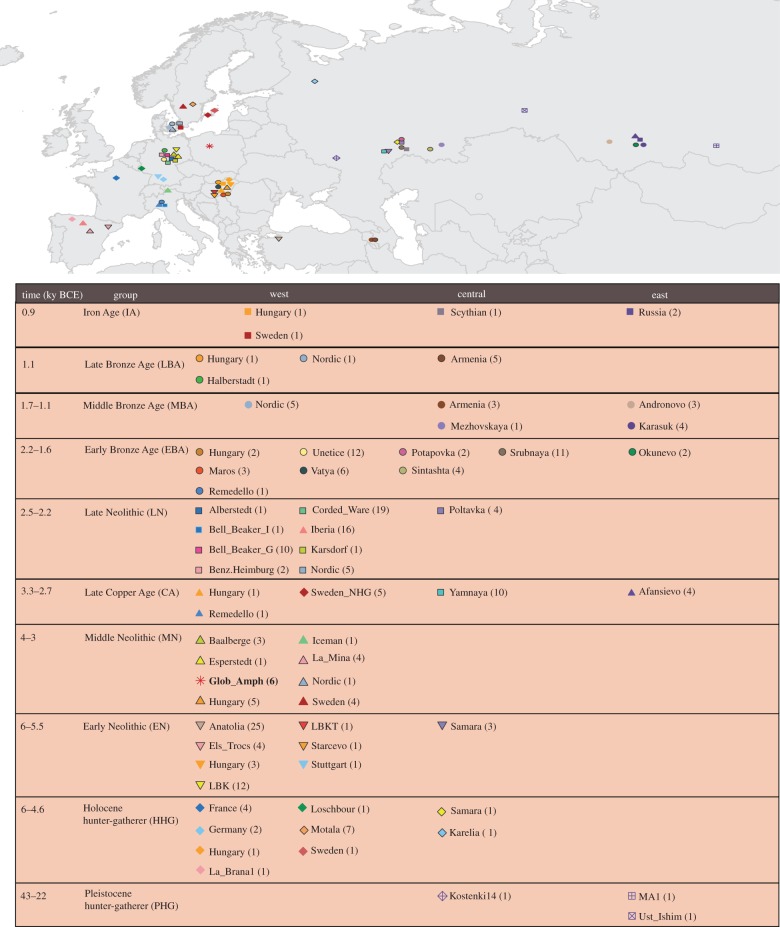
Geographical location and timescale of the ancient individuals. Sampling locations and ages for the ancient samples. The colours and the symbols for each population are the same in all the analyses. In bold, the samples included in the optimized dataset. See also electronic supplementary material, table S2.

To analyse the GAC individuals in the context of ancient and present-day genetic diversity, we merged them with 249 ancient individuals (grouped by archaeological culture and chronology, figure 1 and electronic supplementary material, table S2) and 777 west Eurasian individuals (electronic supplementary material, figure S2 and table S3). All samples were genotyped at 597 573 sites targeted both by the Affymetrix Human Origins array and on the in-solution enrichment reagent used in several ancient DNA studies [26]. We then created an optimized dataset, selecting in each ancient population only SNPs covered in at least one GA individual, which brought the number of SNPs to 350 680. For each ancient population, we then filtered out from this subset the SNPs showing missing genotype in all individuals. We ordered the ancient populations based on the number of SNPs selected by the filtering process, and adding the populations one by one we identified a common subset of SNPs, namely 101 979 SNPs in 39 populations, for a total of 199 ancient individuals spanning from the Pleistocene to the Iron Age (electronic supplementary material, figure S3). Using PLINK [27], we extracted these positions from 777 modern individuals. This way, we assembled two datasets: *AP*, including both ancient and present-day individuals and *A*, including only ancient individuals.

#### (ii) Data analysis

We ran PCA on the *AP* dataset using the snpgdsPCA function in the SNPRelate package [28]. For details on this and other methods, see Data analysis in the electronic supplementary material. To avoid possible confounding effects caused by post-mortem deamination, only transversions were considered, for a total of 18 198 SNPs. Next, we calculated a matrix of genetic distances between pairs of individuals in the dataset *AP*, using the software 4P [29], considering only the SNPs for which both individuals had non-missing genotypes. To account for the non-diploid data of ancient individuals, a random allele was selected for each heterozygous modern individual (using a custom-made Perl script), which made the dataset completely homozygous. The distance matrix was visualized by multidimensional scaling (MDS), using the cmdscale function in R [30].

Population structure was inferred from both datasets using ADMIXTURE [31]; we assumed that the number of clusters ranged between *K*= 2 and *K*= 10, using 10 replicates per *K* with different random seeds. The optimal value of *K* was evaluated through a cross-validation procedure, thus identifying the number of ancestral populations for which the model had the best predictive accuracy. To summarize the degree of genetic relatedness between the GAC samples and the populations of the *A* dataset, we estimated outgroup *f*_3_-statistics using the ADMIXTOOLS program qp3Pop [26], in the form *f*_3_ (X, Globular Amphorae; Mbuti). We also estimated the *f*_3_ statistic considering as target the individuals of the Corded Ware population, in the form *f*_3_ (X, Corded Ware; Mbuti). Maximum-likelihood trees summarizing variation in the *AP* dataset were inferred by TreeMix [32], adding from one to seven migration edges to account for the residual covariance not explained by the tree structure. Finally, zones of increased or decreased genetic similarity between populations with respect to random expectations, corresponding to increased or decreased migrational exchanges, were mapped by EEMS, a method inferring from the data estimated effective migration surfaces [33] (see Data analysis in the electronic supplementary material).

### (c) Mitochondrial data

#### (i) Samples and sequencing

Seventeen specimens, bones and teeth, were selected (electronic supplementary material, table S4) and their mtDNAs were analysed independently in two different laboratories (see Mitochondrial data in the electronic supplementary material and electronic supplementary material, table S5). We collected from the literature ancient mtDNA data from the same populations described in the nuclear data section (figure 1 and electronic supplementary material, table S6). The sequences of 213 samples available in FASTQ format were analysed applying the same pipeline described in the ‘Mitochondrial DNA sequence pre-processing and mapping’ section. A subset of samples, namely 56 individuals belonging to five populations, was extracted from the initial dataset and used together with nine GAC samples for the coalescent simulations and additional exploratory analysis (electronic supplementary material, table S7). Phylogenetic networks, based on nucleotide variation in the two mtDNA datasets, were constructed using the median joining algorithm [34] implemented in Network 5.0 program (http://www.fluxus-technology.com).

#### (ii) Analysis of demographic models

**Figure 2.**
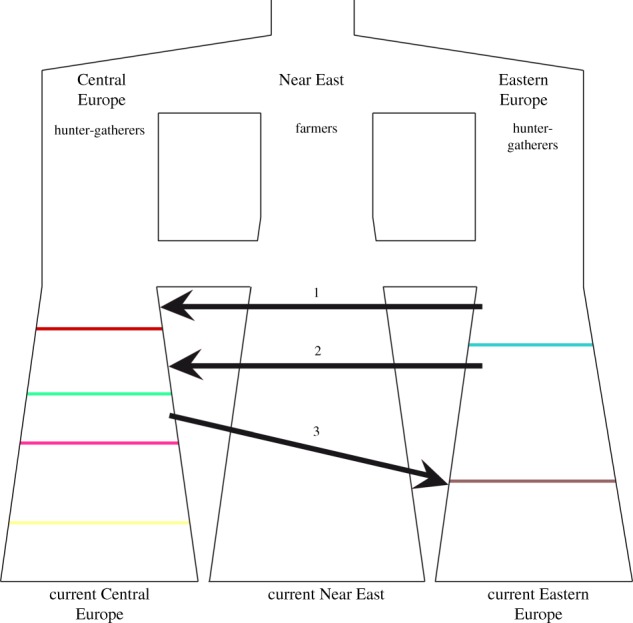
Scheme summarizing the five alternative models compared via ABC random forest. We generated by coalescent simulation mtDNA sequences under five models, differing as to the number of migration events considered. The coloured lines represent the ancient samples included in the analysis, namely Unetice (yellow line), Bell Beaker (purple line), Corded Ware (green line) and Globular Amphorae (red line) from Central Europe, Yamnaya (light blue line) and Srubnaya (brown line) from Eastern Europe. The arrows refer to the three waves of migration tested. Model NOMIG was the simplest one, in which the six populations did not have any genetic exchanges; models MIG1, MIG2 and MIG1, 2 differed from NOMIG in that they included the migration events number 1, 2 (from Eastern to Central Europe, respectively before and after the onset of the GAC), or both. Model MIG2, 3 represents a modification of MIG2 model also including a back migration from Central to Eastern Europe after the development of the Corded Ware culture.

We compared different demographic models, each characterized by a different set of migration events, via approximate Bayesian computation via random forest (ABC-rf) (see electronic supplementary material for details and electronic supplementary material, table S8). In all cases, we assumed some level of genetic continuity across three geographical regions, namely Eastern Europe, Central Europe and the Near East; the models then differed as for the presence of one or two migration events, connecting different regions. The analysis required two main steps; in the first one we estimated the number and the extent of migration waves from the Pontic steppes (arrows 1 and 2 in figure 2); in the second one we quantified the degree of resemblance among Corded Ware people and Early Bronze Age individuals from Eastern Europe, considering the possibility of a third, eastward migration (arrow 3 in figure 2). Other details about the models are in the electronic supplementary material. To compare these models, we applied the ABC-rf considering 50 000 simulations per model and 500 trees in the forest, using the *abcrf* and the *predict* functions provided in the abcrf R package. To evaluate the ability of the ABC-rf procedure to distinguish among the models tested, we calculated the classification error using as PODs each dataset of our reference table. The ability of the selected models to actually generate the observed variation was assessed using linear discriminant analysis (LDA) and PCA. To estimate the models' parameters we selected the best 5000 simulations out of 1 million for each model selected. Posterior probabilities for models and parameters were calculated using R scripts from http://code.google.com/p/popabc/source/browse/#svn%2Ftrunk%2Fscripts, modified by S.G.

## 3. Results

### (a) Genomic data

#### (i) Relationships among individuals and populations

**Figure 3.**
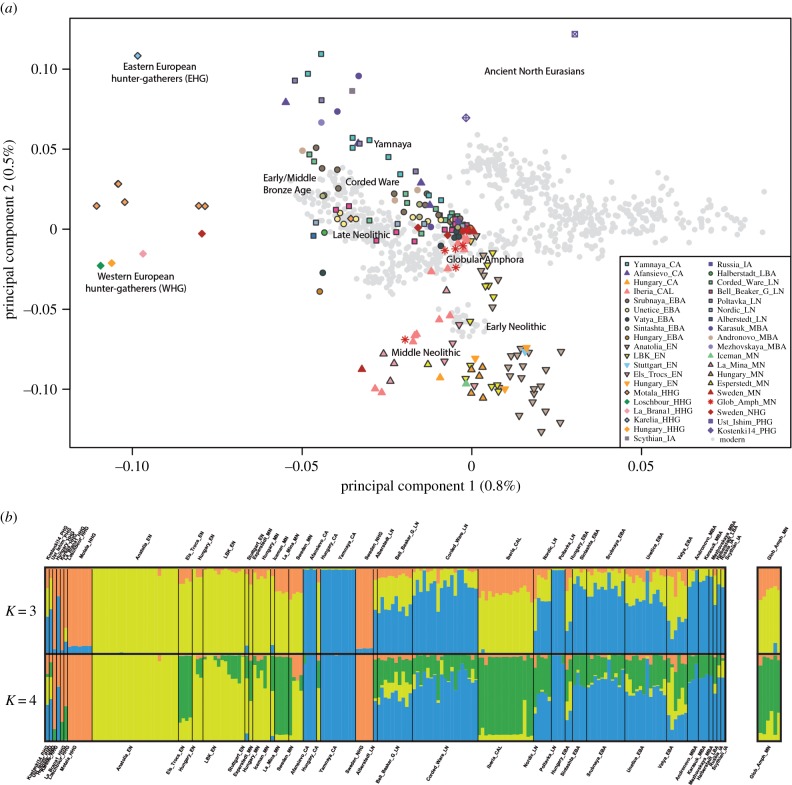
(*a*) Principal component analysis on genomic diversity in ancient and modern individuals. (*b*) *K* = 3,4 ADMIXTURE analysis based only on ancient variation. (*a*) Principal component analysis of 777 modern West Eurasian samples with 199 ancient samples. Only transversions considered in the PCA (to avoid confounding effects of post-mortem damage). We represented modern individuals as grey dots, and used coloured and labelled symbols to represent the ancient individuals. (*b*) Admixture plots at *K* = 3 and *K* = 4 of the analysis conducted only considering the ancient individuals. The full plot is shown in electronic supplementary material, figure S7. The ancient populations are sorted by a temporal scale from Pleistocene to Iron Age. The GAC samples of this study are displayed in the box on the right.

To explore the genetic affinities among ancient and modern-day individuals, we examined a PCA plot of the *AP* dataset. The newly reported GAC individuals fell within a cluster comprising most Early and Middle Neolithic individuals (figure 3*a* and electronic supplementary material, figure S4). As previously observed [20], a clear separation is apparent between hunter-gatherers and samples of more recent periods, with the Bronze Age individuals at the top of the plot, the Late Neolithic samples in a central position and the Early and Middle Neolithic samples at the bottom. We found again a Europe–Near East cline along the principal component 1 in modern populations, and the clustering of early farmers across Europe with present-day Sardinians [18,20,35] (electronic supplementary material, figure S5).

We also computed a matrix of genetic distances between pairs of individuals in the *AP* dataset, considering for each pair of individuals only the shared SNPs. The MDS plot confirms the pattern shown by PCA, again showing three well-differentiated clusters corresponding to the Palaeolithic hunter-gatherers, to the samples spanning from the Late Copper Age to the Bronze Age, and to Middle and Early Neolithic people, including those from the GAC (electronic supplementary material, figure S6).

Clustering by ADMIXTURE [31] of the genotypes in our ancient, *A*, dataset (figure 3*b*; see electronic supplementary material, figure S7 and figure S8 for the complete analysis of both datasets *AP* and *A*), revealed three ancestral components, clearly separating three groups. The orange component is found at high frequencies in hunter-gathering populations such as the Holocene samples from Hungary and in the Motala samples from Sweden. All Early Neolithic populations are characterized by having a large proportion of their genotypes represented by a yellow component, which, strikingly, is also strongly represented in Chalcolithic samples from Iberia consistent with a common origin from first farmers of Anatolia [20]. By contrast, the individuals belonging to the Yamna, Corded Ware, Afanasievo and Andronovo populations, showed a high frequency of a third, blue component, which is consistent with the scenario of a common ancestry in the Pontic steppes followed by westward migration.

Except for one of our Kierzkowo samples (in which it represented about 15% of the genotype), the Steppe-related component was absent altogether in the GAC population. All other GAC samples showed, instead, a mix between a major Early Neolithic component (up to 83%), and the component found at high frequencies among hunter-gatherers (up to 30%). When the ADMIXTURE is asked to cluster the samples into four rather than three groups, the Early Neolithic cluster fissions into two, and the new component (green) is present in the GAC, as well as in several other Western European populations. This second Neolithic component, here referred to as Western Europe Neolithic, accounts for a large share of the ancestry of individuals such as those from Iberia (Iberia_CA), La Mina (LaMina_MN) and Els Trocs (Els_Trocs_EN).

The ADMIXTURE analysis on the *AP* dataset (electronic supplementary material, figure S8) confirms the pattern of relationship between the studied populations. Additional genomic components become apparent, an expected consequence of demographic changes occurred in later prehistoric and in historic periods. However, considering six groups, we could reproduce the clusters previously described in the ancient samples: Early Neolithic (red), hunter-gatherer (yellow), Steppe-related (green) and Western Europe Neolithic (orange). As was the case in the analysis conducted only on ancient samples, the GAC population showed both the Western Europe Neolithic and the Early Neolithic component, with negligible, if any, the presence of the component so strongly associated with the Kurgan migration.

#### (ii) Shared ancestry

**Figure 4.**
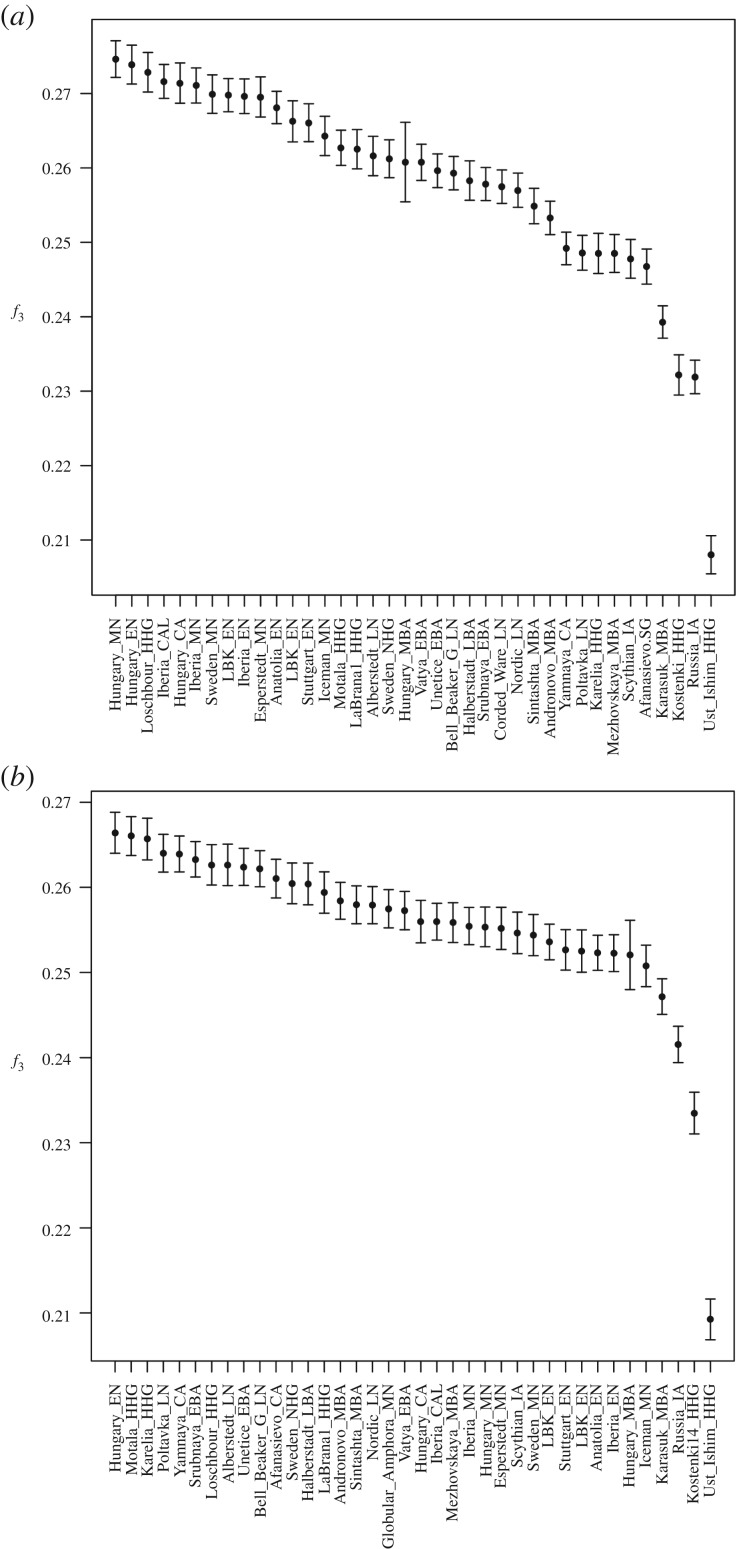
Outgroup *f*_3_ statistics. (*a*) Test in the form *f*_3_ (X, Globular Amphorae; Mbuti). (*b*) Test in the form *f*_3_ (X, Corded Ware; Mbuti), where X is all other ancient populations. Black error bars represent two standard errors.

Moving from individual to population comparisons, we summarized levels of shared genetic ancestry between pairs of populations since their divergence from an African outgroup calculating sets of *f*_3_-statistics, in the form (X, GAC; Mbuti), where X represents, in turn, each ancient population in our dataset. Once again, the GAC people appeared to have more in common with the other Middle Neolithic samples, in particular from Hungary, Iberia and Sweden, than with geographically closer samples (figure 4). A genetic link with the Loschbour sample is also apparent, supporting the hypothesis that, around the Middle Neolithic, farmers of Near-Eastern origin, after a first phase of expansion without admixture [4], began to incorporate in their communities the residual western hunters and gatherers [18]. Remarkably, we did not find evidence of any GAC clear genetic link with the Yamna sample, as well as with any other populations related with the Kurgan Migration Hypothesis. We then calculated the same statistics in the form of *f*_3_ (X, Corded Ware; Mbuti), to verify whether we could detect signals of introgression from the Pontic steppes in the later, Corded Ware population, as already observed by Haak *et al.* [18] and Allentoft *et al*. [21]; a clear genetic link became apparent. Other samples showing high similarity with Corded Ware individuals are the Afanasievo and Andronovo samples. Finally, we confirmed that in the Late Neolithic there is an increased similarity between farmers and hunter-gatherers, a likely consequence of the assimilation of the latter into the former, shown by the high level of drift shared by the Corded Ware sample and the hunter-gatherers from Sweden and Russia.

#### (iii) Inferring migration

These evolutionary links represent departures from a simplistic, tree-like model of population split, followed by divergence in isolation. To account for population contacts after their initial separation, we then added a number of putative migration events to the maximum-likelihood population tree inferred from our data [32]. The initial tree without superimposed gene flow nicely reproduced the three main clusters observed in the ADMIXTURE analysis, namely the Holocene hunters and gatherers (orange component in ADMIXTURE), the populations related to those of the Pontic Steppes (blue component) and the Early and Middle Neolithic populations, including the GAC (yellow component) (electronic supplementary material, figure S9). We then added to the model seven gene flow episodes, each graphically represented by an edge accounting for an additional fraction of the covariance. In this way, we found evidence of genetic exchanges involving hunters and gatherers (e.g. Loschbour) and Middle Neolithic populations, but none of these migration events actually involved the GAC.

Finally, by a graphic method, EEMS [33], we identified zones where the apparent rate of migration was higher or lower than expected under isolation by distance (electronic supplementary material, figure S10). We separately analysed sets of samples of comparable age. Generally, we found patterns consistent with isolation by distance [36] with limited zones where migration rates appeared slightly higher than expected (electronic supplementary material, figure S10*a*–*c* and *d*–*g*). The GAC population followed this trend, showing only an increased gene flow with the contemporary Middle Neolithic samples from Sweden (electronic supplementary material, figure S10*c*). The only evidence for reduced gene flow is an apparent barrier surrounding Hungary in the Copper Age map.

### (b) Mitochondrial data

#### (i) Mitochondrial DNA mapping results

Results obtained from mitochondrial DNA enrichment are summarized in electronic supplementary material, table S4. Samples 6.3 and 7.5 had mean coverage <1 fold and were excluded from further analyses. Among the other samples, the average mtDNA coverage ranged between 6.3 and 244.6, and the average fragment length was between 50.9 and 67.6 bp. As already mentioned, radiocarbon dating of some bone remains found outside the burial chamber indicates they belong to historical times (see ‘Globular Amphorae culture and the archaeological site of Kierzkowo’ in electronic supplementary material). Average fragment lengths were the same for the samples found inside (Neolithic period) and outside (historical period) the burial chamber. By contrast, the two groups of samples differed for the deamination rate at read termini: the proportion of C to T misincorporations is between 30% and 40% in the Neolithic individuals, and only 11% and 12%, respectively, in historical samples 8.8 and 8.9, possibly correlated with their different ages [37]. Sample 6.2, not directly dated but found outside the chamber as well, shows 11% misincorporation, much like the historical samples. No extensive contamination by modern DNA was detected and nine mitochondrial genomes were selected as described in electronic supplementary material for population genetic analysis.

#### (ii) mtDNA data analysis

For 11 out of 17 GAC individuals, mitochondrial DNA was typed by a capture NGS run. The samples were analysed with the NGS pipeline described in Modi *et al*. [38]. Three individuals appeared genealogically related according to whole genome analysis (I2433, mother of I2407 and I2435). Thus, in successive analyses we only considered sample I2433, the one with the highest coverage, bringing the GAC sample size to 9 (electronic supplementary material, table S5).

To formally test the Steppe migration hypothesis, we selected a subset of the mtDNA data including the nine GAC individuals and 56 samples from five populations (see electronic supplementary material, table S7; the complete dataset is in electronic supplementary material, table S6, and the correspondence median network in electronic supplementary material, figure S11), and we ran some preliminary analyses on it. In the neighbour joining (NJ) tree inferred from the *ϕ*_ST_ pairwise distances estimated for this subset, the Early Bronze Age people, represented by the Srubnaya culture, appear connected with the eastern Corded Ware peoples, and also close to the Yamna. The GAC samples are clearly separated from those populations, and show instead a closer relationship with the western, Late Neolithic, Bell Beaker population (electronic supplementary material, figure S12).

The median-joining network [34] (electronic supplementary material, figure S13) shows GAC sequences falling in haplogroups H, J, K, U and W. The relationships between the GAC and other populations of the same time period are evident (electronic supplementary material, figure S14, inset d), especially with the population from Sweden and, although less so, with the Baalberge population from Germany.

#### (iii) Demographic history reconstruction

ABC-rf ([39]; see prior distributions in electronic supplementary material, table S8) gave the strongest support to the MIG2 model (posterior probability = 0.40; the number of votes associated with each model are reported in electronic supplementary material, table S9), involving a single migration from the Pontic steppes into Central Europe, just before the onset of the Corded Ware culture, but after the moment at which our GAC samples are dated. Given the low discrimination power resulting from this four-model comparison (electronic supplementary material, table S10), and to better investigate the relationship between the two models receiving the highest number of votes (i.e. MIG2 and MIG1,2), we also performed a direct comparison between MIG2 and MIG1,2. The classification error was lower (electronic supplementary material, table S11), and the probability associated with the selected model, MIG2, was 0.62 (electronic supplementary material, table S12). The proportion of Corded Ware lineages actually derived from Yamna people was estimated to be 0.33 (median value) or 0.43 (modal value) (electronic supplementary material, table S13).

As a second step, we compared the MIG2 model with a model also including a back migration to Eastern Europe (MIG2,3, figure 2), thus exploring the possibility that the Sintashta are derived directly from an Eastward migration of Corded Ware people, as proposed by Allentoft *et al*. [21]. Because we had no high-quality mitochondrial data for the Sintashta, we chose as a proxy the Srubnaya, which appeared very similar in previous analyses of nuclear variation. The comparison of these two models via ABC-rf marginally favoured MIG2,3, but only with a posterior probability of 0.53 (electronic supplementary material, tables S14 and S15). The estimated median proportions of Yamna related lineages contributing to Corded Ware lineages, and of Corded Ware related lineages contributing to the Srubnaya lineages, were respectively 0.31 and 0.27 (electronic supplementary material, table S16). Note that the value estimated for the proportion of Corded Ware lineages coming from Yamna is consistent between the two models, whereas the estimate obtained for the admixture with Yamna in the Srubnaya mitochondrial genome has low *R*^2^ value, indicating that we might not have enough power to quantify its extent. Both the MIG2 and the MIG2,3 models provided a good ability to reproduce the observed data, as it is shown by the LDA and the PCA plots in electronic supplementary material, figure S15.

## 4. Discussion

In its classical formulation, the Kurgan hypothesis, i.e. a late Neolithic spread of proto-Indo-European languages from the Pontic steppes, regards the GAC people as largely descended from Late Neolithic ancestors from the East, most likely representing the Yamna culture; these populations then continued their Westward movement, giving rise to the later Corded Ware and Bell Beaker cultures. Gimbutas [23] suggested that the spread of Indo-European languages involved conflict, with eastern populations spreading their languages and customs to previously established European groups, which implies some degree of demographic change in the areas affected by the process. The genomic variation observed in GAC individuals from Kierzkowo, Poland, does not seem to agree with this view. Indeed, at the nuclear level, the GAC people show minor genetic affinities with the other populations related with the Kurgan Hypothesis, including the Yamna. On the contrary, they are similar to Early-Middle Neolithic populations, even geographically distant ones, from Iberia or Sweden. As already found for other Late Neolithic populations [18], in the GAC people's genome there is a component related to those of much earlier hunting-gathering communities, probably a sign of admixture with them. At the nuclear level, there is a recognizable genealogical continuity from Yamna to Corded Ware. However, the view that the GAC people represented an intermediate phase in this large-scale migration finds no support in bi-dimensional representations of genome diversity (PCA and MDS), ADMIXTURE graphs, or in the set of estimated *f*_3_-statistics.

For a formal test of these findings, mtDNA data, with its absence of recombination, have valuable properties and allow explicit modelling. Analysis of mitochondrial DNA also benefits from the extensive mathematical methodology that has been developed for studying population history based on such data. Population relationships inferred from mitochondrial data closely resembled those inferred from nuclear data, and so it seemed unlikely that the two datasets may reflect very different demographic events. The models including no migration (NOMIG), or a migration from the Pontic steppes before the onset of the GAC (MIG1) found very limited support in the ABC analysis. The best fit in the first round of simulations was obtained including only a later migration from Yamna, i.e. one not affecting the GAC individuals for which we have information (MIG2), followed by the model in which two subsequent migrations are considered (MIG1,2). Adding, in a second round of simulations, a further episode of gene flow, this time eastwards (MIG2,3), improved the fit, but not dramatically so. The proportion of mtDNA lineages possibly derived from those of the Yamna people is less than 50%.

In short, simulation-based tests on mtDNA variation do not suggest that the GAC people of this study have special links with migrants from the Pontic steppes, but show a direct connection between the Yamna and later Central Europe cultures (Corded Ware and Bell beaker), who derived almost half of their mitochondrial variation from them. The emerging picture is thus one in which migrations from the Pontic steppes into Central Europe left a trace in the genomes of the Corded Ware culture, but not in those of the GAC.

At this stage, it is hard to say whether and to what extent the finding that migrations from the Pontic steppes had little or no demographic impact on the GAC also has implications for the Kurgan hypothesis. There is little doubt that in the Late Neolithic there were indeed migration processes from the Pontic steppes into Central Europe, documented by the archaeological and genetic links between the Yamna and Corded Ware cultures. However, depending on the number of people involved, migrations may or may not leave a recognizable trace in the genetic makeup of a population. The data we provide show that such a trace is not apparent in the genomes of the GAC people. Rather, evolutionary connections are evident between the GAC and other European groups, both at the nuclear and mitochondrial level. In short not all population relationships in the Central European late Neolithic correspond to those proposed in the original Kurgan model. Of course, there is also the possibility that GAC sites other than those investigated in this study might show different genomic features, but at this stage, this is only a matter of speculation. Therefore, either the GAC people pre-existed and were extraneous to the Pontic Steppe migration process envisaged by Gimbutas, or the Pontic steppes migrants' contribution was represented by few individuals, too few indeed to leave a trace in the genetic makeup of the GAC population.

To get a deeper insight into the linguistic changes prompted by Neolithic migration, one should have an idea of the languages spoken in that period, which is currently out of reach. Therefore, our work leaves open the possibility that the GAC was in contact with Yamna, but in this case, the interaction was mostly at the cultural level, entailing very limited migration, if any, contrary to the predictions of Gimbutas' Kurgan hypothesis. One alternative is that that the Pontic steppe migration did not profoundly affect mitochondrial variation, as implied by several studies showing an excess of migrating males in expanding pastoral economies (see Saag *et al*. unpublished data [40] and Kristiansen *et al*. [41], and references therein). However, in this case, the substantial contribution of males should at least result in some degree of similarity between GAC and Yamnaya at the nuclear level, which did not emerge in this study. In both cases, a transmission of cultural traits from the Pontic steppes to the GAC, and later further West, is conceivable and not ruled out by our data; further archaeological work, including studies of other GAC sites, may shed additional light on this.

## Supplementary Material

Electronic Supplementary Information

## Supplementary Material

Supplementary Figures

## Data accessibility

Samples information and GenBank accession numbers of the 14 mtDNA genome sequences reported in this study are summarized in electronic supplementary material, table S4. The nuclear genome data analysed in this study are reported as part of a parallel study that includes the GAC samples among many other groups from eastern and southeastern Europe [25]. All the fastsimcoal input files for the demographic models can be found in the Appendix of electronic supplementary material.
