# Supplementary material for: Genome diversity in the Neolithic Globular Amphorae culture and the spread of Indo-European languages
Source: Proc Biol Sci. 2017 Nov 22;284(1867):20171540. doi: 10.1098/rspb.2017.1540 (PMC5719168; doi:10.1098/rspb.2017.1540)
Supplement: Electronic Supplementary Information [file rspb20171540supp1.pdf]

Genome diversity in the Neolithic Globular Amphorae culture and the spread of Indo-European languages

Francesca Tassi[†,1], Stefania Vai[†,2], Silvia Ghirotto[1], Martina Lari[2], Alessandra Modi[2], Elena Pilli[2], Andrea Brunelli[1], Roberta Rosa Susca[1], Alicja Budnik[3], Damian Labuda[4], Federica Alberti[5], Carles Lalueza-Fox[6], David Reich[7,8], David Caramelli*[2], Guido Barbujani*[1]

[1] Department of Life Sciences and Biotechnology, University of Ferrara, Italy

[2] Department of Biology, University of Firenze, Italy

[3] Department of Human Biology, Cardinal Stefan Wyszyński University, Warsaw, Poland

[4] CHU Sainte-Justine Research Center, Department of Pediatrics, Université de Montréal, Montréal, PQ, Canada H3T 1C5

[5] Department of Evolutionary Biology, Institute for Biochemistry and Biology, Potsdam University, Germany

[6] Institute of Evolutionary Biology, University Pompeu Fabra, Barcelona, Spain

[7] Department of Genetics, Harvard Medical School, Boston, Massachusetts, USA

[8] Howard Hughes Medical Institute, Harvard Medical School, Boston, Massachusetts, USA

[†]These authors contributed equally to this work

Correspondence and requests for materials should be addressed to D.C (email: david.caramelli@unife.it) or to G.B (email: g.barbujani@unife.it)

Supplementary Materials

Globular Amphorae Culture and the archaeological site of Kierzkowo

The process of neolithization of territories in the basins of upper Elbe, Oder and Vistula, was characterised by a shift from settled communities based on farming and husbandry to mobile shepherd communities during the fifth millennium BP. This transformation corresponds to the passage from Funnel Beaker Culture (TRB), through Globular Amphorae Culture (GAC), to Corded Ware Culture. Relationships between these cultural complexes are not yet fully explained. Some scholars, such as Gimbutas, interpreted these changes hypothesizing a migration of pastoral groups from the steppes of southern Ukraine, also associated with the spread of proto-Indo-European languages (Kurgan hypothesis) [1]. The GAC culture assumes a crucial role in this theory. It was distributed across central and eastern Europe, from the Elbe to the middle Dnieper, around 3,400-2,800 BC and was characterized by an apparently mobile economy, animal husbandry dominated by pigs and cattle, presence of a domestic horse, distinctive pottery and burial rituals [2-5].

The archaeological site of Kierzkowo is located in the Żnin district (Kujavia-Pommern voivodeship, North-Western Poland) and represents an excellent example of the Globular Amphorae rituals. The burial context consists of a megalithic barrow of almost 22 meters long and 3-6 meters wide, built from slabs of stone and rocks. A chamber is present inside the barrow, and it is divided by a large stone into two unequal parts.

Three main assemblages of human bones were found in the inner tomb chamber, disposed in separate layers, without anatomical order and often fragmented and mixed. Some bones were also located under the large stone dividing the chamber. A total of 428 bone fragments were identified corresponding to at least 23 individuals of both sexes and of all age categories, probably placed in the barrow as secondary internment. There were some less numerous bones found outside of the chamber. In many instances, human bones were mixed with cattle and swine bones [6].

Seventeen human specimens from Kierzkowo were analysed for mitochondrial and nuclear DNA. After genetic analysis, some of the human bones have been radiocarbon dated at 4,120 ± 30 BP (Beta-430712, sample K3_4), 4,460 ± 30 BP (Beta-430713, sample K8_2a), 4,390 ± 30 BP (Beta-430714, sample K8_4) in agreement with a previous radiocarbon date on animal bone found in the burial of 4,270 ± 40 [7]. Two other human samples collected outside the chamber were dated at 210 ± 30 BP (Beta-430715, sample K8_8) and 130 ± 30 BP (Beta-430716, sample K8_9) indicating that the bone assemblage these samples come from, is not related to the Neolithic context.

Data analysis

Genomic Data

Nuclear DNA analysis was restricted to individuals from whom genome-wide nuclear DNA was successfully obtained according to the criteria presented in Mathieson et al. unpublished data which is the primary report of the nuclear data [8]. During the assembly of the genotypic dataset (referred to as "A" dataset in the M&M section), we went through three different stages, including first a large number of "well covered" SNPs genotyped in a small number of populations, and then increasing twice the sample size, each time at the expense of the average coverage. For each dataset, we ran the whole analysis (PCA, ADMIXTURE, etc,), obtaining consistent results. The results discussed in the text refer to a dataset including 199 samples belonging to 39 populations, each covered at 101,979 SNPs (see figure S3)

ADMIXTURE analysis

The two datasets were analyzed using ADMIXTURE [9]. First, we pruned the data for LD, using PLINK [10], setting windows of 200 SNPs with a step size of 25 SNPs and an r^2 threshold of 0.5 [11]. This filtering yielded a total of 76,231 SNPs for the *AP* dataset and 78,554 SNPs for the *A* dataset. We explored the clustering between *K*= 2 and *K*= 10 using ten replicates fivefold cross-validation at each K using different random seeds. The minimal cross-validation error was found at *K*=3. We comment on the graphs based on *K*= 3 and *K*= 4 clusters, since it is there that the main ancestral components emerge more clearly. Common signals between the different replicates were identified using the LargeKGreedy mode of CLUMPP [12] and plotted using the software Distruct [13].

Outgroup f_3-Statistics

To summarize the degree of genetic relatedness between the GAC samples and the populations of the *A* dataset, we estimated outgroup f_3-statistics using the ADMIXTOOLS program qp3Pop [14], in the form f_3 (X, Globular Amphorae; Mbuti). This statistic measures the amount of genetic drift shared by GAC and population X, after their divergence from an African common ancestor, here approximated by the Mbuti. Standard errors were computed using a weighted block jack-knife approach [15] over 5-Mb blocks. We used the option 'inbreed'=YES, as the ancient samples are represented by randomly sampled alleles rather than diploid genotypes. Using the same setting, we also estimated the f_3 statistic considering as target the individuals of the Corded Ware population, in the form f_3 (X, Corded Ware; Mbuti).

Treemix Analysis

Maximum-likelihood trees summarizing variation in the *AP* dataset were inferred by TreeMix [17]. We rooted the trees with Ust-Ishim. Correction for low samples sizes was turned off (-noss) since some groups were represented by single individuals. We computed standard errors of migration weights (-se) and the

covariance using blocks of 500 SNPs. We tested different numbers of migration edges (from 1 to 7) to account for the residual covariance not explained by the tree structure.

Estimated Effective Migration Surfaces

Zones of increased or decrease genetic similarity between populations with respect to random expectation, corresponding to increased or decreased migrational exchanges, were mapped by EEMS, a method to represent genetic variation as Estimated Effective Migration Surfaces[17]. We divided the ancient dataset into time groups (as seen in figure1) and computed pairwise differences between individuals belonging to the same time set using the *bed2diffs* script available with EEMS from Github at https://github.com/dipetkov/eems. For each time set, we produced an EEMS surface averaging five runs with 500, 700 and 900 demes, as the number of demes simulated during the grid construction phase can influence the scale of the deviation from overall migration detected [36]. Each single run consisted of 200,000 burn-in steps followed by 500,000 MCMC iterations sampled every 10,000 steps. We plotted the averaged EEMS and checked for MCMC convergence using the rEEMSplots enclosed in the EEMS software.

Mitochondrial Data

Seventeen specimens were selected for DNA analysis consisting of bones and teeth (table S1). One fragment from each sample was analyzed in the Molecular Anthropology Laboratory of the University of Florence for mitochondrial DNA and another from the same specimen in the Department of Genetics at Harvard Medical School for mitochondrial and nuclear DNA.

Sample preparation and DNA extraction

In the Molecular Anthropology Laboratory of Florence, bones were cleaned removing the outer layer using a dentist drill with disposable tips. Then each sample was irradiated by ultraviolet light for 45min in a Biolink DNA Crosslinker (Biometra). The inner part of the compact bone from diaphysis and the inner portion of the root for teeth were powdered using the same dentist drill. The DNA was extracted from approximately 50 mg of bone powder following a published silica-based protocol optimized for highly degraded samples [18] and DNA was eluted in 50 µl of TET buffer (10 nM Tris. 1 mM EDTA. 0.05% Tween-20).

Library preparation, mitochondrial and nuclear DNA enrichment and sequencing

Twenty microliters of each extract were converted into double-strand Illumina library without enzymatic damage repair in order to preserve the damage patterns of DNA fragments, using a combination of two indexes per sample following the protocol described in Modi et al 2017 [19]. In Florence no UDG treatment was adopted in order to keep and consequently analyze damage pattern along DNA fragments [20]. In

Harvard, libraries were prepared following a protocol with partial UDG treatment [21]. For each sample, both libraries were then enriched for human mitochondrial DNA (mtDNA) following the bead-capture method described in Maricic et al. 2010 [22]. Mitochondrial DNA enriched libraries were pooled in equal concentration and sequenced on an Illumina MiSeq paired-end run with 2×75+8+8 cycles.

Mitochondrial DNA sequence pre-processing and mapping

Raw reads were processed as in Modi et al. 2017 with a minimum overlap of 10 bp between paired-end reads during merging and discarding sequences below 30 bp. The revised Cambridge Reference Sequence (rCRS, NC_012920.1[23]) was used as reference sequences for mapping using BWA-0.6.29 [24] with -l 1000 -n 0.01 -o 2 parameters setting. After removing PCR duplicates, only mapped reads with mapping quality ≥ 30 were kept and used for base calling and to obtain consensus sequences for the mitochondrial genome. After visual inspection, final base calling was allowed only for a position covered at least by three reads and with a concordance between them of at least 70%. DNA damage pattern was estimated using MapDamage 2.0 [25] and contamination estimation was performed using ContamMix 1.0.10 [26]. To avoid miscalling, all the polymorphic positions (table S5) reported in the vcf output file were then visually inspected. The assembly to the reference was visualized in Tablet [27] and we masked all the positions covered by less than three reads. We applied the IUPAC code, when the concordance across reads was lower than 70%.

In a first step, this workflow was followed for each sample, analyzing separately sequences obtained from the two experimental analyses (Florence and Harvard). A perfect match was found in the mitochondrial profiles obtained from the two results for each samples. For this reason, the two set of reads were merged together to perform a final analysis.

Sample selection for population genetic analysis

For mitochondrial data, samples were selected according to three criteria to be considered for phylogenetic and population genetic analysis: coverage of the genome, degradation pattern and contamination estimate. Therefore, samples 6.3 and 7.5 were discarded owing to their low coverage. All other samples with a mean coverage of at least 6-fold show no extensive modern DNA contamination that could invalidate the reconstruction of the consensus sequence.

Sample 8.8 and 8.9, directly dated and found outside the burial chamber, were discarded for their recent age; they show a misincorporation percentage lower than other samples, possibly correlated with their different age [28]. Sample 6.2 showed a misincorporation rate more similar to that of the historical samples rather than the Neolithic ones. The 6.2 individual was found outside the burial chamber, together

with 8.8 and 8.9. A preliminary analysis based on nuclear data (data not shown) showed samples 8.8, 8.9 and 6.2 clustering together in a separated area of a PCA plot compared to the other samples from Kierzkowo. For these reasons, sample 6.2 was also discarded for further analysis.

Mitochondrial and nuclear DNA profiles for samples 8.4 and 7.1 revealed that the two samples belonged to the same individual. For this reason, results obtained from the two samples were merged together and the I2407 code was used to indicate the individual in the next analyses.

Mitochondrial haplotypes of samples 5.1 (I2433), 8.4/K7.1 (I2407) and 6.1 (I2435) indicated a possible maternal relationship between the individuals. Considering the ages estimated by anthropometric approach for the three individuals, 5.1 (I2433) could probably be the mother of the other two. Nuclear DNA analysis confirmed the relationship and indicated samples 7.6 (I2440) as the father of this family. For this reason, 8.4/K7.1 (I2407) and 6.1 (I2435) were discarded for population genetic analysis and only 5.1 (I2433) (mother) and 7.6 (I2440) (father) were kept as genetically unrelated (table S5).

Mitochondrial genetic diversity

Explorative analyses were conducted pooling the nine mtDNA GAC samples with a total of 213 mtDNA ancient samples collected from published papers (see detail in table S6) for the same populations described in the nuclear data section. The samples collected belong to 51 populations from different Neolithic and pre-Neolithic cultures (figure 1). The consensus sequences for the samples collected available in the FASTQ format were obtained applying the same quality filters used for the GAC samples. Complete sequences were aligned to rCRS by MUSCLE v3.8.31 [29] and manually checked. Haplogroup assignment, based on PhyloTree Build 16 [30], was estimated in HaploGrep [31] for each sample in the dataset (table S6). Pairwise φST distances were calculated by the Arlequin software [32] with Kimura's 2P distance correction. Using 100,000 replications, a bootstrapped Neighbour Joining (NJ) tree was inferred from the φST pairwise distances thanks to the *nj* and *boot.phylo* functions available within the ape R cran package [33, 34]. Phylogenetic networks, based on nucleotide variation in the two mtDNA datasets, were constructed using the Median Joining algorithm [35] implemented in Network 5.0 program (http://www.fluxus-technology.com). The ε value was set to 0 and the transversions were weighted 3x the weight of transitions. Networks were subjected to maximum parsimony post-analysis. For the eleven GAC the polymorphic positions, with respect to the rCRS, were also annotated (see table S5).

Analysis of demographic models

We compared alternative models of migration from the Pontic steppes by ABC Random Forest. This new

ABC-rf algorithm is based on a machine learning tool (called "Random Forest" [36]) that learns from a reference table of simulations coming from different scenarios how to predict the most suitable model at each possible value of a set of covariates (i.e. all summary statistics used to summarize the data). Once the model is selected, the posterior probability of that model is predicted by another Random Forest, obtained from regressing the probability of error of the same covariates. This procedure differs from the classical algorithm in which model choice is based on a logistic regression to estimate models' posterior probabilities. Random forest uses a classification algorithm, which allows one to overcome the difficulties in the choice of the summary statistics, while gaining a larger discriminative power among the competing models [37]. The simulated datasets for each scenario were obtained using *fastsimocal2* [38], in the software package ABCtoolbox [39].

For the models' comparison we considered a total of 100,000 serial coalescent simulations, i.e. 50,000 simulations for each model, and 500 trees in the forest. We performed the model selection in two sequential analyses. First, we compared four models considering zero, one or two waves of migration from Eastern Europe; then we compared the most probable model within this set with one also considering a back migration from Central to Eastern Europe after the development of the Corded ware culture (figure 2). Under all five models, we considered a single population which split 50,000 years ago into three main demes (Central Europe, Near East and Eastern Europe), evolving at a constant population size. Starting from 10,000 years ago, the Near East deme started to grow, mimicking the demographic expansion following the development of agriculture. We modelled the admixture between Near Eastern farmers and Central-Eastern Europe hunter-gatherers happening about 8,500 years ago [40], with a proportion of farmer lineages into the hunter-gatherer demes of 40% [41]. From this moment on, also the Central and the Eastern Europe demes started to grow in size.

The Unetice sample (7 individuals), the Bell Beaker sample (11 individuals), the Corded Ware sample (16 individuals) and the GAC sample (9 individuals) were placed in the Central Europe deme 3,500, 3,800, 4,000, 4,400 yBP, respectively. The Srubnaya sample (11 individuals) and the Yamna sample (11 individuals) were in the Eastern Europe deme, respectively 3,700 and 4,300 yBP. Under Model NOMIG, populations simply evolved as described above, without further contacts. Under Model MIG1 we simulated a migration wave from Eastern to Central Europe before the onset of the GA culture (i.e. from 5,000 to 6,000 yBP; arrow number 1 in figure. 2); under Model MIG2 we simulated another migration in the same direction, after the onset of the GA culture and prior to the Corded Ware sampling time (from 4,000 to 4,300 yBP; arrow number 2 in figure 2). Model MIG1,2 considered both waves of migration simulated under the previous models. We compared these 4 models by ABC-rf, considering 50,000 simulations per model and 500 trees in the forest, using the functions provided in the abcrf R package. We also directly compared MIG2 and MIG1,2, since these two models provided the highest fit with the observed data. The model

emerging from this analysis as the one with the highest posterior probability (MIG2) was then compared with a model also incorporating a back migration from Central to Eastern Europe after the sampling of the Corded Ware people and before the onset of the Srubnaya culture; this model is referred to as MIG2,3. All the ABCToolbox input files are available in the *Appendix*. Summary statistics were calculated by arlsumstat [42], describing both within-population (number of haplotypes, haplotype diversity, total and private number of segregating sites, Tajima's D, and average number of pairwise differences for each population), and between-population diversity (F_{ST} and mean number of pairwise differences between populations). We validated the model selection procedure calculating the classification error through the *abcrf* function of the abcrf R package. To do this, we used as pseudo-observed datasets each dataset of our reference table. To verify whether the selected models are able to generate the observed data, we performed a linear discriminant analysis (LDA) and a principal component analysis (PCA) of the statistics generated by each of these two models, and we verified whether the observed values fall within the variation generated by the tested scenarios. The LDA plot were generated using the *plot.abcrf* function of the abcrf R package, whereas the PCA plot were generated by the *PCA* function of the FactoMineR R package. To estimate the models' parameters for models MIG2 and MIG2,3, we ran further simulations, approximately 1 million simulations per model. Model parameters were estimated by a locally weighted multivariate regression [43] after a logtan transformation [44] of the 5,000 best-fitting simulations from either model. To calculate the posterior probabilities for models and parameters we used R scripts [33] from http://code.google.com/p/popabc/source/browse/#svn%2Ftrunk%2Fscripts, modified by SG. When estimating model's parameters we reduced the dimensionality of the space of statistics via Partial Least Square (PLS) transformation [45]. Under this approach, we defined a set of orthogonal linear- combinations of summary statistics best explaining the variance in the model parameter space. After the inspection of the Root Mean Square Error Plots, we selected 5 PLS to calculate the posterior probabilities of model's parameters. The parameters shared by different models have the same prior distributions; the complete list of estimated models' parameters and of the associated prior distributions is presented in supplementary table S8; we used a generation time of 29 years.

Supplementary tables

Table S1. Description of the analyzed specimens, anthropometric sex and age determination and available radiocarbon dating. Sample ID (corresponding to the nomenclature used during the archaeological excavation) and Lab code (corresponding to the laboratory label assigned to successfully genotyped GAC individuals) are indicated. Note that Lab code is the same for sample 7.1 and 8.4, because the mitochondrial and nuclear profiles indicated they belong to the same individual. Samples 6.2, 6.3, 7.5, 8.8 and 8.9 were discarded for population genetic analysis because of their low coverage or recent radiocarbon dating.

Sample ID	Lab code	Anatomical element	Sex and Age estimation	Conventional radiocarbon age	Calibrated radiocarbon Result (95% probability)
**3.1**	I2301	Left ulna	Male (50- X years)		
**3.4**	I2403	Left femur	Male	4120 ± 30 BP (Beta – 430712)	Cal BC 2,870 to 2,800 (Cal BP 4,820 to 4,750), Cal BC 2,780 to 2,575 (Cal BP 4,730 to 4,525)
**5.1**	I2433	Tooth/mandible	Female (50-60 years)		
**5.3a/5.3b**	I2434	Cranium/right femur	Female (20-30 years)		
**6.1**	I2435	femur	Female (30-50 years)		
**6.2**		right ulna	Female over 25		
**6.3**		mandible	Female		
**7.1**	I2407	maxilla	Youngster (14-19 years)		
**7.5**		Right humerus	Youngster (14-19 years)		
**7.6**	I2440	Right humerus	Adult		
**8.1**	I2801	Right femur	Child (2 years)		
**8.2**	I2405	Tibia	Child (8 years)	4460 ± 30 BP (Beta – 430713)	Cal BC 3,335 to 3,210 (Cal BP 5,285 to 5,160), Cal BC 3,190 to 3,150 (Cal BP 5,140 to 5,100), Cal BC 3,140 to 3,020 (Cal BP 5,090 to 4,970)
**8.3**	I2803	Right tibia	Child (14 years)		
**8.4**	I2407	Left femur	Child (10-14 years)	4390 ± 30 BP (Beta – 430713)	Cal BC 3,095 to 2,915 (Cal BP 5,045 to 4,865)
**8.5**	I2441	Left pelvis	Newborn		
**8.8**		Left femur	Child (infant)	210 ± 30 BP (Beta – 430715)	Cal AD 1,645 to 1,685 (Cal BP 305 to 265), Cal AD 1,735 to 1,805 (Cal BP 215 to 145), Cal AD 1,930 to Post 1,950 (Cal BP 20 to Post 0)
**8.9**		Right femur	Child (2-3 years)	130 ± 30 BP (Beta – 430716)	Cal AD 1,670 to 1,780 (Cal BP 280 to 170), Cal AD 1,800 to Post 1,950 (Cal BP 150 to Post 0)

Table S2 - Details of ancient samples used in analyses. Related to figure 1.

Sample	Sex	Epoch	Country	Location	DateBP	Analysis label	SNPnuclear_source
**I2403**	M	MN	Poland	Kierzkowo	4870-4575	Glob Amph MN	[8]
**I2405**	M	MN	Poland	Kierzkowo	5335-5020	Glob Amph MN	[8]
**I2433**	F	MN	Poland	Kierzkowo	5100-4900	Glob Amph MN	[8]
**I2434**	F	MN	Poland	Kierzkowo	5400-4800	Glob Amph MN	[8]
**I2435**	M	MN	Poland	Kierzkowo	5100-4900	Glob Amph MN	[8]
**I2440**	M	MN	Poland	Kierzkowo	5100-4900	Glob Amph MN	[8]
**I1503**	M	IA	Hungary	Ludas-Varju-Dulo	2980-2830	Hungary IA	[47]
**RISE174**	F	IA	Sweden	Oxie 7	2427-2611	Sweden IA	[48]
**RISE492**	M	IA	Russia	Sabinka 2	2396-2209	Russia IA	[48]
**RISE504**	M	IA	Russia	Kytmanovo	2721-2889	Russia IA	[48]
**I0247**	M	IA	Russia	Nadezhdinka, Volga Steppes, Samara	2375-2203	Scythian IA	[49]
**I0099**	M	LBA	Germany	Halberstadt-Sonntagsfeld	3193-2979	Halberstadt LBA	[49]
**I1504**	M	LBA	Hungary	Ludas-Varju-Dulo	3270-3110	Hungary LBA	[49]
**RISE276**	M	LBA	Denmark	Trundholm mose II	2794-2547	Nordic LBA	[48]
**RISE396**	F	LBA	Armenia	Kapan	3192-2937	Armenia LBA	[48]
**RISE397**	M	LBA	Armenia	Kapan	3048-2855	Armenia LBA	[48]
**RISE407**	F	LBA	Armenia	Norabak	3115-2895	Armenia LBA	[48]
**RISE408**	M	LBA	Armenia	Norabak	3209-3009	Armenia LBA	[48]
**RISE412**	F	LBA	Armenia	Noratus	3193-2945	Armenia LBA	[48]
**RISE21**	F	MBA	Denmark	Karlstrup	3426-3281	Nordic MBA	[48]
**RISE47**	M	MBA	Denmark	Sebber skole	3499-3324	Nordic MBA	[48]
**RISE175**	M	MBA	Sweden	Abekås I	3395-3132	Nordic MBA	[48]
**RISE207**	M	MBA	Sweden	Ängamöllan	3493-3302	Nordic MBA	[48]
**RISE210**	M	MBA	Sweden	Ängamöllan	3432-3292	Nordic MBA	[48]
**RISE413**	M	MBA	Armenia	Nerquin Getashen	3906-3698	Armenia MBA	[48]
**RISE416**	M	MBA	Armenia	Nerquin Getashen	3643-3445	Armenia MBA	[48]
**RISE423**	M	MBA	Armenia	Nerquin Getashen	3402-3211	Armenia MBA	[48]
**RISE493**	M	MBA	Russia	Sabinka 2	3531-3427	Karasuk MBA	[48]
**RISE494**	M	MBA	Russia	Sabinka 2	3416-3268	Karasuk MBA	[48]
**RISE496**	F	MBA	Russia	Arban 1	3414-3261	Karasuk MBA	[48]
**RISE502**	F	MBA	Russia	Bystrovka	3496-3306	Karasuk MBA	[48]
**RISE503**	F	MBA	Russia	Kytmanovo	3727-3511	Andronovo	[48]
**RISE505**	F	MBA	Russia	Kytmanovo	3746-3626	Andronovo	[48]
**RISE512**	M	MBA	Russia	Kytmanovo	3446-3298	Andronovo	[48]
**RISE523**	F	MBA	Russia	Kapova cave	3598-3398	Mezhovskaya	[48]
**I1502**	F	EBA	Hungary	Kompolt-Kigyoser	4190-3980	Hungary EBA	[49]
**I0115**	F	EBA	Germany	Esperstedt	3954-3760	Unetice EBA	[49]
**I0117**	F	EBA	Germany	Esperstedt	4272-4039	Unetice EBA	[49]
**I0804**	M	EBA	Germany	Eulau	4137-3965	Unetice EBA	[49]
**I0803**	F	EBA	Germany	Eulau	4132-3942	Unetice EBA	[49]
**I0164**	F	EBA	Germany	Quedlinburg VIII	4023-3894	Unetice EBA	[49]
**I0116**	M	EBA	Germany	Esperstedt	4134-3939	Unetice EBA	[49]
**I0047**	F	EBA	Germany	Halberstadt-Sonntagsfeld	4111-3891	Unetice EBA	[49]
**RISE109**	F	EBA	Poland	Wojkowice	3954-3772	Unetice EBA	[48]
**RISE139**	F	EBA	Poland	Chociwel	4135-3923	Unetice EBA	[48]
**RISE145**	M	EBA	Poland	Polwica	4188-3958	Unetice EBA	[48]
**RISE150**	F	EBA	Poland	Przeclawice	3885-3693	Unetice EBA	[48]
**RISE154**	F	EBA	Poland	Szczepankowice	3925-3765	Unetice EBA	[48]
**RISE247**	M	EBA	Hungary	Százhalombatta-Földvár	3746-3611	Vatya EBA	[48]
**RISE254**	M	EBA	Hungary	Százhalombatta-Földvár	4128-3909	Vatya EBA	[48]
**RISE479**	M	EBA	Hungary	Erd 4	4000-3500	Vatya EBA	[48]
**RISE480**	F	EBA	Hungary	Erd 4	3700-3500	Vatya EBA	[48]
**RISE483**	F	EBA	Hungary	Erd 4	4000-3500	Vatya EBA	[48]
**RISE484**	F	EBA	Hungary	Erd 4	4000-3500	Vatya EBA	[48]
**RISE349**	F	EBA	Hungary	Battonya Vörös Oktober	4034-3784	Hungary EBA	[48]
**RISE371**	F	EBA	Hungary	Szöreg - C (Sziv Utca)	4136-3941	Maros EBA	[48]
**RISE373**	F	EBA	Hungary	Szöreg - C (Sziv Utca)	3886-3696	Maros EBA	[48]
**RISE374**	M	EBA	Hungary	Szöreg - C (Sziv Utca)	3866-3619	Maros EBA	[48]
**RISE386**	M	EBA	Russia	Bulanovo	4298-4045	Sintashta EBA	[48]
**RISE391**	F	EBA	Kazakhsta	Tanabergen II	4120-3887	Sintashta EBA	[48]
**RISE394**	F	EBA	Russia	Bulanovo	3949-3754	Sintashta EBA	[48]
**RISE395**	F	EBA	Russia	Bol'shekaraganskii	3960-3756	Sintashta EBA	[48]
**RISE486**	M	EBA	Italy	Remedello di Sotto	4134-3773	Remedello EBA	[48]
**RISE515**	F	EBA	Russia	Verkhni Askiz Village	4340-4145	Okunevo EBA	[48]
**RISE516**	F	EBA	Russia	Verkhni Askiz Village	4201-4036	Okunevo EBA	[48]
**I0235**	F	EBA	Russia	Rozhdestvenno I, Samara Steppes, Samara	3850-3600	Srubnaya EBA	[49]
**I0234**	F	EBA	Russia	Rozhdestveno I, Samara Steppes, Samara	3850-3600	Srubnaya EBA	[49]
**I0431**	F	EBA	Russia	Spiridonovka II, Samara River, Samara	3850-3600	Srubnaya EBA	[49]
**I0430**	M	EBA	Russia	Spiridonovka II, Samara River, Samara	3850-3600	Srubnaya EBA	[49]
**I0424**	M	EBA	Russia	Uvarovka I, Samara River, Samara	3850-3600	Srubnaya EBA	[49]
**I0232**	M	EBA	Russia	Novoselki, Northern Forest, Samara	3850-3200	Srubnaya EBA	[49]
**I0360**	M	EBA	Russia	Spiridonovka IV, Samara River, Samara	3850-3200	Srubnaya EBA	[49]
**I0358**	F	EBA	Russia	Spiridonovka IV, Samara River, Samara	3906-3631	Srubnaya EBA	[49]
**I0361**	M	EBA	Russia	Spiridonovka IV, Samara River, Samara	3850-3200	Srubnaya EBA	[49]
**I0359**	F	EBA	Russia	Spiridonovka IV, Samara River, Samara	3850-3200	Srubnaya EBA	[49]
**I0422**	F	EBA	Russia	Barinovka I, Samara River, Samara	3850-3200	Srubnaya EBA	[49]
**I0418**	F	EBA	Russia	Utyevka IV, Samara River, Samara	4125-3769	Potapovka EBA	[49]
**I0246**	M	EBA	Russia	Utyevka VI, Samara_River, Samara	4469-3928	Potapovka EBA	[49]
**I0118**	F	LN	Germany	Alberstedt	4471-4246	Alberstedt LN	[49]

**I0171**	F	LN	Germany	Benzingerode-Heimburg	4287-4041	BenzigerodeHei		[49]
**I0059**	F	LN	Germany	Benzingerode-Heimburg	4337-4138	BenzigerodeHei		[49]
**I0806**	M	LN	Germany	Quedlinburg VII	4431-4150	Els Trocs EN		[49]
**I0113**	F	LN	Germany	Quedlinburg XII	4346-4033	Bell Beaker G LN		[49]
**I0112**	F	LN	Germany	Quedlinburg XII	4457-4142	Bell Beaker G LN		[49]
**I0060**	F	LN	Germany	Rothenschirmbach	4428-4149	Bell Beaker G LN		[49]
**I0111**	F	LN	Germany	Rothenschirmbach	4475-4204	Bell Beaker G LN		[49]
**I0108**	F	LN	Germany	Rothenschirmbach	4575-4299	Bell Beaker G LN		[49]
**I1546**	F	LN	Germany	Benzingerode-Heimburg	4500-4050	Bell Beaker G LN		[49]
**I0805**	M	LN	Germany	Quedlinburg VII	4467-4142	Bell Beaker G LN		[49]
**I1549**	F	LN	Germany	Benzingerode-Heimburg	4500-4050	Bell Beaker G LN		[49]
**I1530**	M	LN	Germany	Rothenschirmbach	4458-4140	Bell Beaker G LN		[49]
**I2478**	M	LN	Italy	via Guidorossi, Parma	4200-3930	Bell Beaker I LN		[8]
**I0550**	F	LN	Germany	Karsdorf	4570-4471	Karsdorf LN		[49]
**I0106**	F	LN	Germany	Esperstedt	4464-4210	Corded Ware LN		[49]
**I0049**	F	LN	Germany	Esperstedt	4464-4210	F3 LN		[49]
**I0103**	F	LN	Germany	Esperstedt	4578-4468	Corded Ware LN		[49]
**I0104**	M	LN	Germany	Esperstedt	4559-4296	Corded Ware LN		[49]
**I1542**	M	LN	Germany	Esperstedt	4500-4050	Corded Ware LN		[49]
**I1536**	M	LN	Germany	Esperstedt	4500-4050	Corded Ware LN		[49]
**I1544**	M	LN	Germany	Esperstedt	4500-4050	Corded Ware LN		[49]
**I1538**	M	LN	Germany	Esperstedt	4500-4050	Corded Ware LN		[49]
**I1539**	F	LN	Germany	Esperstedt	4625-4291	Corded Ware LN		[49]
**I1534**	M	LN	Germany	Esperstedt	4500-4050	Corded Ware LN		[49]
**I1540**	M	LN	Germany	Esperstedt	4500-4050	Corded Ware LN		[49]
**I1532**	M	LN	Germany	Esperstedt	4500-4050	Corded Ware LN		[49]
**RISE00**	F	LN	Estonia	Sope	-	Corded Ware LN		[48]
**RISE1**	M	LN	Poland	Oblaczkowo	4865-4578	Corded Ware LN		[48]
**RISE431**	M	LN	Poland	Leki Male	4286-4048	Corded Ware LN		[48]
**RISE434**	M	LN	Germany	Tiefbrunn	4880-4630	Corded Ware LN		[48]
**RISE435**	F	LN	Germany	Tiefbrunn	4863-4498	Corded Ware LN		[48]
**RISE436**	M	LN	Germany	Tiefbrunn	4868-4580	Corded Ware LN		[48]
**RISE446**	M	LN	Germany	Bergrheinfeld	4829-4465	Corded Ware LN		[48]
**RISE42**	M	LN	Denmark	Marbjerg	4191-3972	Nordic LN		[48]
**RISE71**	F	LN	Denmark	Falshöj	4196-4023	Nordic LN		[48]
**RISE97**	F	LN	Sweden	Fredriksberg	4025-3885	Nordic LN		[48]
**RISE98**	M	LN	Sweden	L Beddinge 56	4275-4032	Nordic LN		[48]
**RISE179**	M	LN	Sweden	Abekås I	4010-3776	Nordic LN		[48]
**I0371**	M	LN	Russia	Grachevka II, Sok River, Samara	4872-4583	Poltavka LN		[49]
**I0126**	M	LN	Russia	Kutuluk III, Kutuluk River, Samara	4867-4486	Poltavka LN		[49]
**I0440**	M	LN	Russia	Lopatino II, Sok River, Samara	4887-4666	Poltavka LN		[49]
**I0374**	M	LN	Russia	Nikolaevka III, Samara River, Samara	4800-4200	Poltavka LN		[49]
**I1282**	M	LN	Spain	El Mirador Cave, Atapuerca, Burgos	4900-4346	Iberia CAL		[49]
**I1302**	M	LN	Spain	El Mirador Cave, Atapuerca, Burgos	4900-4346	Iberia CAL		[49]
**I1276**	F	LN	Spain	El Mirador Cave, Atapuerca, Burgos	4900-4346	Iberia CAL		[49]
**I1284**	M	LN	Spain	El Mirador Cave, Atapuerca, Burgos	4900-4346	Iberia CAL		[49]
**I1280**	F	LN	Spain	El Mirador Cave, Atapuerca, Burgos	4900-4346	Iberia CAL		[49]
**I1277**	M	LN	Spain	El Mirador Cave, Atapuerca, Burgos	4568-4346	Iberia CAL		[49]
**I1272**	F	LN	Spain	El Mirador Cave, Atapuerca, Burgos	4857-4496	Iberia CAL		[49]
**I1281**	F	LN	Spain	El Mirador Cave, Atapuerca, Burgos	4865-4575	Iberia CAL		[49]
**I1300**	F	LN	Spain	El Mirador Cave, Atapuerca, Burgos	4900-4346	Iberia CAL		[49]
**I1271**	F	LN	Spain	El Mirador Cave, Atapuerca, Burgos	4900-4346	Iberia CAL		[49]
**I1303**	M	LN	Spain	El Mirador Cave, Atapuerca, Burgos	4900-4346	Iberia CAL		[49]
**I0581**	M	LN	Spain	El Mirador Cave, Atapuerca, Burgos	4900-4679	Iberia CAL		[49]
**ATP16**	F	LN	Spain	El Portalon Cave, Sierra de Atapuerca	5261-4916	Iberia CAL		[50]
**ATP17**	M	LN	Spain	El Portalon Cave, Sierra de Atapuerca	5007-4871	Iberia CAL		[50]
**ATP20**	F	LN	Spain	El Portalon Cave, Sierra de Atapuerca	4289-4050	Iberia CAL		[50]
**Matojo**	M	LN	Spain	El Portalon Cave, Sierra de Atapuerca	5010-5879	Iberia CAL		[50]
**I1497**	F	LCA	Hungary	Apc-Berekalya I	4900-4700	Hungary CA		[49]
**I0231**	M	LCA	Russia	Ekaterinovka, Southern Steppe, Samara	4921-4762	Yamna CA		[49]
**I0370**	M	LCA	Russia	Ishkinovka I, Eastern Orenburg, Pre-Ural	5300-4700	Yamna CA		[49]
**I0441**	F	LCA	Russia	Kurmanaevka III, Buzuluk, Samara	5010-4622	Yamna CA		[49]
**I0444**	M	LCA	Russia	Kutuluk I, Kutuluk River, Samara	5335-4882	Yamna CA		[49]
**I0439**	M	LCA	Russia	Lopatino I, Sok River, Samara	5321-4921	Yamna CA		[49]
**I0357**	F	LCA	Russia	Lopatino I, Sok River, Samara	5090-4913	Yamna CA		[49]
**I0429**	M	LCA	Russia	Lopatino I, Sok River, Samara	5339-4918	Yamna CA		[49]
**I0438**	M	LCA	Russia	Luzkhi I, Samara River, Samara	5021-4635	Yamna CA		[49]
**I0443**	M	LCA	Russia	Lopatino II, Sok River, Samara	5300-4700	Yamna CA		[49]
**RISE240**	F	LCA	Russia	Sukhaya Termista I	4880-4632	Yamna CA		[48]
**Ajvide52**	M	LCA	Sweden	Gotland	4900-4600	Sweden NHG		[51]
**Ajvide53**	F	LCA	Sweden	Gotland	4900-4600	Sweden NHG		[51]
**Ajvide58**	M	LCA	Sweden	Gotland	4900-4600	Sweden NHG		[51]
**Ajvide59**	M	LCA	Sweden	Gotland	4900-4600	Sweden NHG		[51]
**Ajvide70**	M	LCA	Sweden	Gotland	4900-4600	Sweden NHG		[51]
**RISE487**	M	LCA	Italy	Remedello di Sotto	5483-5107	Remedello CA		[48]
**RISE508**	F	LCA	Russia	River Kuyum	5331-4935	Afansievo CA		[48]
**RISE509**	F	LCA	Russia	Bateni	4887-4677	Afansievo CA		[48]
**RISE510**	F	LCA	Russia	Bateni	4851-4468	Afansievo CA		[48]
**RISE511**	F	LCA	Russia	Bateni	4909-4679	Afansievo CA		[48]
**Iceman**	M	MN	Italy	..	-	Iceman MN		[52]
**Gokhem2**	F	MN	Sweden	Gokhem, Vastergotland	5050-4750	Sweden MN		[51]
**Gokhem4**	M	MN	Sweden	Gokhem, Vastergotland	5050-4750	Sweden MN		[51]
**Gokhem5**	F	MN	Sweden	Gokhem, Vastergotland	5280-4890	Sweden MN		[51]

**Gokhem7**	F	MN	Sweden	Gokhem, Vastergotland	5050-4750	Sweden MN	[51]
**I0405**	M	MN	Spain	La Mina	5900-5600	La Mina MN	[19]
**I0407**	F	MN	Spain	La Mina	5900-5600	La Mina MN	[49]
**I0408**	F	MN	Spain	La Mina	5900-5600	La Mina MN	[49]
**I0406**	M	MN	Spain	La Mina	5900-5600	La Mina MN	[49]
**I0172**	M	MN	Germany	Esperstedt	5360-5086	Esperstedt MN	[49]
**I0807**	M	MN	Germany	Esperstedt	5970-5710	Baalberge MN	[49]
**I0559**	M	MN	Germany	Quedlinburg IX	5652-5527	Baalberge MN	[49]
**I0560**	F	MN	Germany	Quedlinburg IX	5640-5376	Baalberge MN	[49]
**I1500**	M	MN	Hungary	Kompolt-Kigyoser	7210-6990	Hungary MN	[49]
**I1499**	F	MN	Hungary	Garadna	7210-7010	Hungary MN	[49]
**I1495**	M	MN	Hungary	Apc-Berekalya I	6490-6360	Hungary MN	[49]
**I1498**	F	MN	Hungary	Debrecen Tocopart Erdoalja	7290-7060	Hungary MN	[49]
**I1496**	M	MN	Hungary	Apc-Berekalya I	7300-6950	Hungary MN	[49]
**RISE61**	M	MN	Denmark	Kyndelöse	4851-4492	Nordic MN	[48]
**I0018**	F	EN	Germany	Viesenhaeuser Hof, Stuttgart-Muehlhausen	7310-7070	Stuttgart EN	[53]
**I0176**	F	EN	Hungary	Szemely-Hegyes	7207-6944	LBKT EN	[49]
**I0409**	F	EN	Spain	Els Trocs	7310-7218	Els Trocs EN	[49]
**I0412**	M	EN	Spain	Els Trocs	7308-7080	Els Trocs EN	[49]
**I0410**	M	EN	Spain	Els Trocs	7295-7066	Els Trocs EN	[49]
**I0413**	F	EN	Spain	Els Trocs	7302-7074	Els Trocs EN	[49]
**I0795**	M	EN	Germany	Karsdorf	7216-7036	LBK EN	[49]
**I0054**	F	EN	Germany	Unterwiederstedt	7222-7022	LBK EN	[49]
**I0046**	F	EN	Germany	Halberstadt-Sonntagsfeld	7212-6989	LBK EN	[49]
**I0048**	M	EN	Germany	Halberstadt-Sonntagsfeld	7211-7009	LBK EN	[49]
**I0056**	M	EN	Germany	Halberstadt-Sonntagsfeld	7212-7006	LBK EN	[49]
**I0057**	F	EN	Germany	Halberstadt-Sonntagsfeld	7218-7019	LBK EN	[49]
**I0100**	F	EN	Germany	Halberstadt-Sonntagsfeld	7202-6852	LBK EN	[49]
**I0659**	M	EN	Germany	Halberstadt-Sonntagsfeld	7211-6963	LBK EN	[49]
**I0821**	M	EN	Germany	Halberstadt-Sonntagsfeld	7201-6850	LBK EN	[49]
**I0022**	F	EN	Germany	Viesenhaeuser Hof, Stuttgart-Muehlhausen	7500-6800	LBK EN	[49]
**I0026**	F	EN	Germany	Viesenhaeuser Hof, Stuttgart-Muehlhausen	7500-6800	LBK EN	[49]
**I0025**	F	EN	Germany	Viesenhaeuser Hof, Stuttgart-Muehlhausen	7500-6800	LBK EN	[49]
**I1508**	F	EN	Hungary	Berettyóújfalu-Morotva-Liget	7710-7570	Hungary EN	[49]
**I1505**	F	EN	Hungary	Polgar Ferenci hat	7290-7050	Hungary EN	[49]
**I1506**	F	EN	Hungary	Polgar Ferenci hat	7310-7070	Hungary EN	[49]
**I0174**	M	EN	Hungary	Alsonyek-Bataszek, Mernoki telep	7702-7536	Starcevo EN	[49]
**I0434**	M	EN	Russia	Khvalynsk II, Volga River, Samara	7200-6000	Samara EN	[49]
**I0433**	M	EN	Russia	Khvalynsk II, Volga River, Samara	7200-6000	Samara EN	[49]
**I0122**	M	EN	Russia	Khvalynsk II, Volga River, Samara	7200-6000	Samara EN	[49]
**I1581**	F	EN	Turkey	Barcin	8500-8200	Anatolia EN	[49]
**I1583**	M	EN	Turkey	Barcin	8500-8200	Anatolia EN	[49]
**I1580**	F	EN	Turkey	Barcin	8500-8200	Anatolia EN	[49]
**I1585**	F	EN	Turkey	Barcin	8500-8200	Anatolia EN	[49]
**I1579**	F	EN	Turkey	Barcin	8500-8200	Anatolia EN	[49]
**I1100**	F	EN	Turkey	Barcin	8500-8200	Anatolia EN	[49]
**I1102**	M	EN	Turkey	Barcin	8500-8200	Anatolia EN	[49]
**I1099**	M	EN	Turkey	Barcin	8500-8200	Anatolia EN	[49]
**I1103**	M	EN	Turkey	Barcin	8500-8200	Anatolia EN	[49]
**I1101**	M	EN	Turkey	Barcin	8500-8200	Anatolia EN	[49]
**I1097**	M	EN	Turkey	Barcin	8500-8200	Anatolia EN	[49]
**I0744**	M	EN	Turkey	Barcin	8500-8200	Anatolia EN	[49]
**I1096**	M	EN	Turkey	Barcin	8500-8200	Anatolia EN	[49]
**I1098**	F	EN	Turkey	Barcin	8500-8200	Anatolia EN	[49]
**I0708**	M	EN	Turkey	Barcin	8500-8200	Anatolia EN	[49]
**I0745**	M	EN	Turkey	Barcin	8500-8200	Anatolia EN	[49]
**I0746**	M	EN	Turkey	Barcin	8500-8200	Anatolia EN	[49]
**I0707**	F	EN	Turkey	Barcin	8500-8200	Anatolia EN	[49]
**I0709**	M	EN	Turkey	Barcin	8500-8200	Anatolia EN	[49]
**I0725**	F	EN	Turkey	Mentese	8400-7600	Anatolia EN	[49]
**I0736**	F	EN	Turkey	Barcin	8500-8200	Anatolia EN	[49]
**I0726**	F	EN	Turkey	Mentese	8400-7600	Anatolia EN	[49]
**I0723**	M	EN	Turkey	Mentese	8400-7600	Anatolia EN	[49]
**I0724**	M	EN	Turkey	Mentese	8400-7600	Anatolia EN	[49]
**I0727**	M	EN	Turkey	Mentese	8400-7600	Anatolia EN	[49]
**I0001**	M	HHG	Luxembo	Echternach	8210-7990	Loschbour HHG	[53]
**Ire8**	M	HHG	Sweden	Gotland	5100-4150	Sweden HHG	[51]
**I0061**	M	HHG	Russia	Yuzhnyy Oleni Ostrov, Karelia	8850-8000	Karelia HHG	[49]
**I0124**	M	HHG	Russia	Lebyanzhinka IV, Sok River, Samara	7657-7541	Samara HHG	[49]
**LaBrana1**	M	HHG	Spain	La Brana-Arintero, Leon	7990-7740	La Brana1 HHG	[54]
**I1507**	M	HHG	Hungary	Tiszaszolos-Domahaza	7780-7640	Hungary HHG	[49]
**I0013**	M	HHG	Sweden	Motala	7898-7531	Motala HHG	[49]
**I0011**	F	HHG	Sweden	Motala	7898-7531	Motala HHG	[49]
**I0015**	M	HHG	Sweden	Motala	7898-7531	Motala HHG	[49]
**I0012**	M	HHG	Sweden	Motala	7898-7531	Motala HHG	[49]
**I0014**	F	HHG	Sweden	Motala	7898-7531	Motala HHG	[49]
**I0017**	M	HHG	Sweden	Motala	7720-7630	Motala HHG	[49]
**I0017**	M	HHG	Sweden	Motala	7720-7630	Motala HHG	[53]
**BerryAuBac**	M	HHG	France	Berry Au Bac	7370-7220	France HHG	[55]
**Ranchot88**	F	HHG	France	Ranchot	10290-9980	France HHG	[55]
**LesCloseau**	F	HHG	France	Les Closeaux	10290-9610	France HHG	[55]
**Chaudardes**	M	HHG	France	Chaudardes	8410-8100	France HHG	[55]
**Bockstein**	F	HHG	Germany	Swabian Jura, Baden-Wu_rttemberg, Bockstein-	8420-8210	Germany HHG	[55]

**Ofnet**	F	HHG	Germany	Ofnet	8480-8110	Germany HHG	[55]
**MA1**	M	PHG	Russia	Mal'ta	24570-	MA1 PHG	[56]
**Kostenki14**	M	PHG	Russia	Kostenki	38730-	Kostenki14 PHG	[55]
**Ust_Ishim**	M	PHG	Russia	Ust'-Ishim	47530-	Ust Ishim PHG	[55]

Table S3 - Overview of Modern West Eurasians genotyped on the Human Origins array.

Population	Geographic_label	n.
**Abkhasian**	EastEurope	9
**Adygei**	Russia	17
**Albanian**	EastEurope	6
**Armenian**	EastEurope	10
**Ashkenazi_Jew**	EastEurope	7
**Balkar**	Russia	10
**Basque**	WestEurasia	29
**BedouinA**	MiddleEast	25
**BedouinB**	MiddleEast	19
**Belarusian**	EastEurope	10
**Bulgarian**	EastEurope	10
**Canary_Islanders**	SouthEurope	2
**Chechen**	Russia	9
**Croatian**	EastEurope	10
**Cypriot**	SouthEurope	8
**Czech**	EastEurope	10
**Druze**	MiddleEast	39
**English**	NorthEurope	10
**Estonian**	NorthEurope	10
**Finnish**	NorthEurope	7
**French**	WestEurasia	32
**Georgian**	EastEurope	10
**Georgian_Jew**	EastEurope	7
**Greek**	SouthEurope	20
**Hungarian**	EastEurope	20
**Icelandic**	NorthEurope	12
**Iranian**	MiddleEast	8
**Iranian_Jew**	MiddleEast	9
**Iraqi_Jew**	MiddleEast	6

Population	Geographic_label	n.
**Italian_North**	SouthEurope	20
**Italian_South**	SouthEurope	1
**Jordanian**	MiddleEast	9
**Kumyk**	Russia	8
**Lebanese**	MiddleEast	8
**Lezgin**	Russia	9
**Libyan_Jew**	NorthAfrica	9
**Lithuanian**	EastEurope	10
**Maltese**	SouthEurope	8
**Mordovian**	Russia	10
**Moroccan_Jew**	NorthAfrica	6
**North_Ossetian**	Russia	10
**Norwegian**	NorthEurope	11
**Orcadian**	WestEurasia	13
**Palestinian**	MiddleEast	38
**Russian**	Russia	22
**Sardinian**	WestEurasia	27
**Saudi**	MiddleEast	8
**Scottish**	NorthEurope	4
**Sicilian**	SouthEurope	11
**Spanish**	SouthEurope	53
**Spanish_North**	SouthEurope	5
**Syrian**	MiddleEast	8
**Tunisian_Jew**	NorthAfrica	7
**Turkish**	MiddleEast	56
**Turkish_Jew**	MiddleEast	8
**Ukrainian**	EastEurope	9
**Yemenite_Jew**	MiddleEast	8

Table S4. Mitochondrial DNA enrichment results

Sample	Lab	mtDNA	Missing	Fragment	5'	Contaminatio	Contaminati	mtDNA	GenBank ID
**3.1**	I2301	12.99	83	67.6	32	6.56	11.80-2.62	H1b	MF114211
**3.4**	I2403	67.39	15	63.8	33	0.08	1.61-0.01	U5b2b1	MF114212
**5.1**	I2433	172.32	3	61.5	39	3.89	5.21-2.85	H28	MF114213
**5.3**	I2434	64.79	5	65.2	32	1.31	2.27-0.71	U5b1d1	MF114214
**6.1**	I2435	21.00	435	59.8	30	0.00	0.01-0.00	H28	MF114215
**6.2**		82.05	0	67.0	11	0.39	0.05-0.01	J1c2	MF114222
**6.3**		0.35	16383	61.2	-	-	-	-	-
**7.1**	I2407	124.76	2	61.0	38	2.30	2.61-2.04	H28	MF114220
**7.5**		0.44	16262	54.3	-	-	-	-	-
**7.6**	I2440	37.41	133	66.3	23	0.61	1.13-0.29	H1b	MF114216
**8.1**	I2801	13.93	318	56.9	40	0.80	0.89-0.67	H1b	MF114217
**8.2**	I2405	17.05	281	50.9	33	2.30	5.47-0.39	W5	MF114218
**8.3**	I2803	6.27	1389	66.7	38	0.94	2.67-0.20	H	MF114219
**8.4**	I2407	35.95	12	64.2	32	0.34	1.62-0.02	H28	MF114220
**8.5**	I2441	244.58	1	64.0	31	3.86	4.18-3.49	K1b1a1	MF114221
**8.8**		221.18	0	63.7	11	0.50	10.57-0.07	U5b2a2b1	MF114223
**8.9**		6.94	1126	62.1	12	0.22	2.71-0.02	U3b	MF114224

Table S5. Globular Amphorae samples mtDNA variant positions with respect to the rCRS

Sample ID	Range	Haplogroup	Polymorphisms
**I2301**	1-16569	H1	263G 750G 1438G 3010A 4769G 8860G 15326G 16519C
**I2403**	1-16569	U5b2b1a1	73G 150T 263G 750G 1438G 1721T 2706G 3197C 3861G 4769G 7028T 7768G 8860G 9477A 11467G 11653G 11719A 12308G 12372A 12634G 13617C 13630G 13637G 14182C 14766T 15326G 15497A 16270T 16292T 16362C
**I2405**	1-16569	W5	73G 189G 263G 709A 750G 1243C 1438G 2706G 3505G 4769G 5046A 5460A 6528T 7028T 8251A 8860G 8994A 11674T 11719A 11947G 12414C 12705T 14766T 15326G 15775G 15884C 16223T 16292T 16362C 16519C
**I2433**<br>**I2407**<br>**I2435**	1-16569	H28a	186A 263G 750G 1438G 4769G 8715C 8860G 11191T 15326G 16093C 16519C
**I2434**	1-16569	U5b1d1a	73G 150T 263G 742C 750G 1438G 2706G 3197C 3441G 4769G 5437T 5656G 5918C 7028T 7768G 8860G 9477A 11467G 11719A 12308G 12372A 13617C 14182C 14766T 15326G 15355A 15721C 16270T
**I2440**	1-16569	H1b	146C 263G 750G 1438G 3010A 4769G 8860G 15326G 16356C 16519C
**I2441**	1-16569	K1b1a1	73G 152C 263G 750G 1189C 1438G 1811G 2706G 3480G 4769G 5913A 7028T 8429T 8805G 8860G 9055A 9698C 9962A 10289G 10398G 10550G 11299C 11467G 11719A 11923G 12308G 12372A 13967T 14167T 14766T 14798C 15257A 15326G 15946T 16093C 16224C 16311C 16319A 16463G 16519C
**I2801**	1-16569	H1b	263G 750G 1438G 3010A 4769G 8860G 15326G 16093C 16356C 16519C
**I2803**	1-16569	H	263G 750G 1438G 4769G 8860G 11215N 15326G 16093C 16519C

Table S6. Dataset of mtDNA ancient samples.

Sample	Analysis label	Country	Geo_area	Haplogroup	dateBP	Source
**RISE511**	Afansievo_CA	Russia	East	J2a2a	4909-4679	[48]
**I0051**	Alberstedt_LN	Germany	Central	H3b	4494-4344	[11]
**I0118**	Alberstedt_LN	Germany	Central	H+16311	4471-4246	[49]
**I0707**	Anatolia_EN	Turkey	Central	K1	8500-8200	[49]
**I0708**	Anatolia_EN	Turkey	Central	N1b1a	8500-8200	[49]
**I0709**	Anatolia_EN	Turkey	Central	U3	8500-8200	[49]
**I0723**	Anatolia_EN	Turkey	Central	X2m2	8400-7600	[49]
**I0724**	Anatolia_EN	Turkey	Central	K1a4	8400-7600	[49]
**I0725**	Anatolia_EN	Turkey	Central	N1a1a1	8400-7600	[49]
**I0726**	Anatolia_EN	Turkey	Central	H	8400-7600	[49]
**I0727**	Anatolia_EN	Turkey	Central	K1a2	8400-7600	[49]
**I0736**	Anatolia_EN	Turkey	Central	N1a1a1a	8500-8200	[49]
**I0744**	Anatolia_EN	Turkey	Central	J1c11	8500-8200	[49]
**I0745**	Anatolia_EN	Turkey	Central	U8b1b1	8500-8200	[49]
**I0746**	Anatolia_EN	Turkey	Central	K1a	8500-8200	[49]
**I1096**	Anatolia_EN	Turkey	Central	N1a1a1	8500-8200	[49]
**I1097**	Anatolia_EN	Turkey	Central	W1+119	8500-8200	[49]
**I1098**	Anatolia_EN	Turkey	Central	X2d	8500-8200	[49]
**I1099**	Anatolia_EN	Turkey	Central	T2b	8500-8200	[49]
**I1100**	Anatolia_EN	Turkey	Central	K1a	8500-8200	[49]
**I1101**	Anatolia_EN	Turkey	Central	T2b	8500-8200	[49]
**I1102**	Anatolia_EN	Turkey	Central	K1a3a	8500-8200	[49]
**I1103**	Anatolia_EN	Turkey	Central	K1b1b1	8500-8200	[49]
**I1579**	Anatolia_EN	Turkey	Central	K1a+150	8500-8200	[49]
**I1580**	Anatolia_EN	Turkey	Central	H5	8500-8200	[49]
**I1581**	Anatolia_EN	Turkey	Central	U3	8500-8200	[49]
**I1583**	Anatolia_EN	Turkey	Central	K1a2	8500-8200	[49]
**I1585**	Anatolia_EN	Turkey	Central	J1c	8500-8200	[49]
**RISE505**	Andronovo_MBA	Russia	East	U4a1b	3746-3626	[48]
**RISE408**	Armenia_LBA	Armenia	East	I5c	3209-3009	[48]
**RISE412**	Armenia_LBA	Armenia	East	U4c1a	3193-2945	[48]
**I0212**	Baalberge_MN	Germany	Central	T2c1d1	5944-5852	[11]
**I0556**	Baalberge_MN	Germany	Central	U8a1	5950-5400	[11]
**I0557**	Baalberge_MN	Germany	Central	U5b2a2	5950-5400	[11]
**I0559**	Baalberge_MN	Germany	Central	H+16311	5652-5527	[49]
**I0560**	Baalberge_MN	Germany	Central	T2e1	5640-5376	[49]
**I0808**	Baalberge_MN	Germany	Central	H7d	5950-5400	[11]
**I0822**	Baalberge_MN	Germany	Central	K1e	5630-5581	[11]
**I0060**	Bell_Beaker_G_LN	Germany	Central	K1a2c	4428-4149	[49]
**I0108**	Bell_Beaker_G_LN	Germany	Central	H5a3	4575-4299	[49]
**I0111**	Bell_Beaker_G_LN	Germany	Central	H3ao	4475-4204	[49]
**I0112**	Bell_Beaker_G_LN	Germany	Central	H13a1a2	4457-4142	[49]
**I0113**	Bell_Beaker_G_LN	Germany	Central	J1c5	4346-4033	[49]
**I0805**	Bell_Beaker_G_LN	Germany	Central	H1	4467-4142	[49]
**I0806**	Bell_Beaker_G_LN	Germany	Central	H1	4431-4150	[49]
**I1530**	Bell_Beaker_G_LN	Germany	Central	H3ao	4458-4140	[49]
**I1546**	Bell_Beaker_G_LN	Germany	Central	U5a1b1	4500-4050	[49]
**I1549**	Bell_Beaker_G_LN	Germany	Central	W1c1	4500-4050	[49]
**RISE564**	Bell_Beaker_G_LN	Germany	Central	H+16311	4550-3950	[48]
**I0058**	BenzigerodeHeimburg_LN	Germany	Central	H1e	4283-4146	[11]
**I0059**	BenzigerodeHeimburg_LN	Germany	Central	H1	4337-4138	[49]
**I0171**	BenzigerodeHeimburg_LN	Germany	Central	U5a1a2a	4287-4041	[49]
**RISE00**	Corded_Ware_LN	Estonia	Central/East	H5a1	-	[48]
**I0049**	Corded_Ware_LN	Germany	Central	X2b4	4464-4210	[49]
**I0050**	Corded_Ware_LN	Germany	Central	U5a2d	4800-4050	[11]
**I0103**	Corded_Ware_LN	Germany	Central	W6a	4578-4468	[49]
**I0104**	Corded_Ware_LN	Germany	Central	U4b1a1a1	4559-4296	[49]
**I0106**	Corded_Ware_LN	Germany	Central	T2a1b1	4464-4210	[49]
**I1532**	Corded_Ware_LN	Germany	Central	J1c2e	4500-4050	[49]
**I1534**	Corded_Ware_LN	Germany	Central	K1a1b2a	4500-4050	[49]
**I1536**	Corded_Ware_LN	Germany	Central	U5a1g2	4500-4050	[49]
**I1538**	Corded_Ware_LN	Germany	Central	J1c5	4500-4050	[49]
**I1539**	Corded_Ware_LN	Germany	Central	J1c1b1a	4625-4291	[49]
**I1540**	Corded_Ware_LN	Germany	Central	J1c5f	4500-4050	[49]
**I1542**	Corded_Ware_LN	Germany	Central	K1b1a	4500-4050	[49]
**I1544**	Corded_Ware_LN	Germany	Central	J1c3j	4500-4050	[49]
**RISE435**	Corded_Ware_LN	Germany	Central	J1b1a1	4863-4498	[48]
**RISE446**	Corded_Ware_LN	Germany	Central	U5b1c2	4829-4465	[48]
**I0409**	Els_Trocs_EN	Spain	West	J1c3	7310-7218	[49]
**I0410**	Els_Trocs_EN	Spain	West	T2c1d+152	7295-7066	[49]
**I0412**	Els_Trocs_EN	Spain	West	N1a1a1	7308-7080	[49]
**I0413**	Els_Trocs_EN	Spain	West	V	7302-7074	[49]
**I0172**	Esperstedt_MN	Germany	Central	T2b	5360-5086	[49]
**BerryAuBac**	France_HHG	France	Central	U5b1a	7370-7220	[58]
**Chaudardes1**	France_HHG	France	Central	U5b1b	8410-8100	[58]
**LesCloseaux3**	France_HHG	France	Central	U5a2	10290-9610	[58]
**Ranchot88**	France_HHG	France	Central	U5b1	10290-9980	[58]
**Bockstein**	Germany_HHG	Germany	Central	U5b1d1	8420-8210	[58]
**Ofnet**	Germany_HHG	Germany	Central	U5b1d1	8480-8110	[58]

**I0099**	Halberstadt_LBA	Germany	Central	H23	3193-2979	[49]
**I1497**	Hungary_CA	Hungary	Central	H	4900-4700	[49]
**I1502**	Hungary_EBA	Hungary	Central	K1c1	4190-3980	[49]
**I1505**	Hungary_EN	Hungary	Central	J1c5	7290-7050	[49]
**I1506**	Hungary_EN	Hungary	Central	U5b2c	7310-7070	[49]
**I1508**	Hungary_EN	Hungary	Central	K1a1	7710-7570	[49]
**I1507**	Hungary_HHG	Hungary	Central	R1b1	7780-7640	[49]
**I1504**	Hungary_LBA	Hungary	Central	K1a1a	3270-3110	[49]
**I1495**	Hungary_MN	Hungary	Central	N1a1a1a	6490-6360	[49]
**I1496**	Hungary_MN	Hungary	Central	K1a3a3	7300-6950	[49]
**I1498**	Hungary_MN	Hungary	Central	H	7290-7060	[49]
**I1499**	Hungary_MN	Hungary	Central	X2b-T226C	7210-7010	[49]
**I1500**	Hungary_MN	Hungary	Central	J1c1	7210-6990	[49]
**ATP12-1420**	Iberia_CAL	Spain	West	H3c	5010-5879	[50]
**ATP16**	Iberia_CAL	Spain	West	X2c2	5261-4916	[50]
**ATP17**	Iberia_CAL	Spain	West	H2	5007-4871	[50]
**ATP2**	Iberia_CAL	Spain	West	U5b3	4849-4628	[50]
**ATP20**	Iberia_CAL	Spain	West	U5a1c1a	4289-4050	[50]
**ATP3**	Iberia_CAL	Spain	West	K1a2b	5466-5312	[50]
**ATP7**	Iberia_CAL	Spain	West	J1c1b1	5295-4894	[50]
**I0581**	Iberia_CAL	Spain	West	X2b+226	4900-4679	[49]
**I1271**	Iberia_CAL	Spain	West	K1a+195	4900-4346	[49]
**I1272**	Iberia_CAL	Spain	West	K1b1a	4857-4496	[49]
**I1276**	Iberia_CAL	Spain	West	H3c3	4900-4346	[49]
**I1277**	Iberia_CAL	Spain	West	H3	4568-4346	[49]
**I1280**	Iberia_CAL	Spain	West	J1c1	4900-4346	[49]
**I1281**	Iberia_CAL	Spain	West	H1t	4865-4575	[49]
**I1282**	Iberia_CAL	Spain	West	H3	4900-4346	[49]
**I1284**	Iberia_CAL	Spain	West	H3	4900-4346	[49]
**I1300**	Iberia_CAL	Spain	West	K1a2a	4900-4346	[49]
**I1302**	Iberia_CAL	Spain	West	J2b1a3	4900-4346	[49]
**I1303**	Iberia_CAL	Spain	West	U3a1	4900-4346	[49]
**I1314**	Iberia_CAL	Spain	West	J2a1a1	4880-4630	[49]
**Iceman**	Iceman_MN	Italy	West	K1f	-	[59]
**RISE495**	Karasuk_MBA	Russia	East	D4j1	3350-2850	[48]
**RISE497**	Karasuk_MBA	Russia	East	A+152+16362	3350-2850	[48]
**I0061**	Karelia_HHG	Russia	East	C	8850-8000	[49]
**I0550**	Karsdorf_LN	Germany	Central	T1a1	4570-4471	[49]
**Kostenki14**	Kostenki14_PHG	Russia	East	U2	38730-36310	[60]
**LaBrana1**	La_Brana1_HHG	Spain	West	U5b2c1	7990-7740	[54]
**I0404**	La_Mina_MN	Spain	West	J2a1a1	5900-5600	[11]
**I0405**	La_Mina_MN	Spain	West	K1a1b1	5900-5600	[49]
**I0406**	La_Mina_MN	Spain	West	H1	5900-5600	[49]
**I0407**	La_Mina_MN	Spain	West	K1b1a1	5900-5600	[49]
**I0408**	La_Mina_MN	Spain	West	U5b1	5900-5600	[49]
**I0019**	LBK_EN	Germany	Central	H+16311	7500-6800	[11]
**I0020**	LBK_EN	Germany	Central	T2e	7500-6800	[11]
**I0021**	LBK_EN	Germany	Central	T2b	7500-6800	[11]
**I0022**	LBK_EN	Germany	Central	T2+150	7500-6800	[49]
**I0023**	LBK_EN	Germany	Central	H	7500-6800	[11]
**I0024**	LBK_EN	Germany	Central	W1+119	7500-6800	[11]
**I0025**	LBK_EN	Germany	Central	T2b	7500-6800	[49]
**I0026**	LBK_EN	Germany	Central	T2b	7500-6800	[49]
**I0027**	LBK_EN	Germany	Central	H40	7500-6800	[11]
**I0046**	LBK_EN	Germany	Central	T2c1+146	7212-6989	[49]
**I0048**	LBK_EN	Germany	Central	K1a+195	7211-7009	[49]
**I0054**	LBK_EN	Germany	Central	J1c17	7222-7022	[49]
**I0056**	LBK_EN	Germany	Central	T2b	7212-7006	[49]
**I0100**	LBK_EN	Germany	Central	N1a1a1a	7202-6852	[49]
**I0101**	LBK_EN	Germany	Central	U5a1+@16192	7500-6775	[11]
**I0102**	LBK_EN	Germany	Central	N1a1a1a3	7030-6948	[11]
**I0659**	LBK_EN	Germany	Central	N1a1a1a2	7211-6963	[49]
**I0795**	LBK_EN	Germany	Central	H1	7216-7036	[49]
**I0796**	LBK_EN	Germany	Central	H+152	7500-6775	[11]
**I0797**	LBK_EN	Germany	Central	H46b	7500-6775	[49]
**I0820**	LBK_EN	Germany	Central	W1+119	7298-7247	[11]
**I0821**	LBK_EN	Germany	Central	X2d	7201-6850	[49]
**I1550**	LBK_EN	Germany	Central	K1a2	7500-6775	[49]
**I0176**	LBKT_EN	Hungary	Central	N1a1a1a3	7207-6944	[49]
**I0001**	Loschbour_HHG	Luxembourg	Central	U5b1a	8210-7990	[16]
**MA1**	MA1_PHG	Russia	East	U	24570-24140	[56]
**RISE373**	Maros_EBA	Hungary	Central	K1a2a	3886-3696	[48]
**RISE524**	Mezhovskaya_MBA	Russia	East	J2b1a	3250-2650	[48]
**I0011**	Motala_HHG	Sweden	Central	U5a1	7898-7531	[49]
**I0012**	Motala_HHG	Sweden	Central	U2e1h	7898-7531	[49]
**I0014**	Motala_HHG	Sweden	Central	U5a2d	7898-7531	[49]
**I0016**	Motala_HHG	Sweden	Central	U5a2	7898-7531	[11]
**I0017**	Motala_HHG	Sweden	Central	U2e1h	7720-7630	[49]
**RISE98**	Nordic_LN	Sweden	Central	K1b1a1	4275-4032	[48]
**RISE210**	Nordic_MBA	Sweden	Central	T2a1a	3432-3292	[48]
**RISE516**	Okunevo_EBA	Russia	East	H6a1b	4201-4036	[48]
**I0126**	Poltavka_LN	Russia	East	H6a2	4867-4486	[49]
**I0371**	Poltavka_LN	Russia	East	U2d2	4872-4583	[49]

**I0374**	Poltavka_LN	Russia	East	H13a1a	4800-4200	[49]
**I0440**	Poltavka_LN	Russia	East	I3a	4887-4666	[49]
**I0246**	Potapovka_EBA	Russia	East	C	4469-3928	[49]
**I0418**	Potapovka_EBA	Russia	East	T1a1	4125-3769	[49]
**I0122**	Samara_EN	Russia	East	H2a1	7200-6000	[49]
**I0433**	Samara_EN	Russia	East	U5a1i	7200-6000	[49]
**I0434**	Samara_EN	Russia	East	U4	7200-6000	[49]
**I0247**	Scythian_IA	Russia	East	G2a+152	2375-2203	[49]
**RISE391**	Sintashta_EBA	Kazakhstan	East	N1a1a1a1	4120-3887	[48]
**RISE386**	Sintashta_EBA	Russia	East	J1c1b1a	4298-4045	[48]
**RISE392**	Sintashta_EBA	Russia	East	J2b1a2a	4126-3896	[48]
**RISE394**	Sintashta_EBA	Russia	East	U2e1e	3949-3754	[48]
**RISE395**	Sintashta_EBA	Russia	East	U2e1h	3960-3756	[48]
**I0232**	Srubnaya_EBA	Russia	East	U5a1f2	3850-3200	[49]
**I0234**	Srubnaya_EBA	Russia	East	I1a1	3850-3600	[49]
**I0235**	Srubnaya_EBA	Russia	East	K1b2a	3850-3600	[49]
**I0358**	Srubnaya_EBA	Russia	East	H6a1a	3906-3631	[49]
**I0359**	Srubnaya_EBA	Russia	East	U5a2a1	3850-3200	[49]
**I0360**	Srubnaya_EBA	Russia	East	U5a1	3850-3200	[49]
**I0361**	Srubnaya_EBA	Russia	East	H5b	3850-3200	[49]
**I0422**	Srubnaya_EBA	Russia	East	T1a1	3850-3200	[49]
**I0424**	Srubnaya_EBA	Russia	East	T2b4+152	3850-3600	[49]
**I0430**	Srubnaya_EBA	Russia	East	H3g	3850-3600	[49]
**I0431**	Srubnaya_EBA	Russia	East	H2b	3850-3600	[49]
**I0174**	Starcevo_EN	Hungary	Central	N1a1a1	7702-7536	[49]
**Ire8**	Sweden_HHG	Sweden	Central	U4	5100-4150	[51]
**Gokhem2**	Sweden_MN	Sweden	Central	H1c	5050-4750	[51]
**Gokhem5**	Sweden_MN	Sweden	Central	K1e	5280-4890	[51]
**Gokhem7**	Sweden_MN	Sweden	Central	H24	5050-4750	[51]
**Ajvide52**	Sweden_NHG	Sweden	Central	V	4900-4600	[51]
**Ajvide58**	Sweden_NHG	Sweden	Central	U4	4900-4600	[51]
**Ajvide70**	Sweden_NHG	Sweden	Central	U4	4900-4600	[51]
**I0047**	Unetice_EBA	Germany	Central	V	4111-3891	[49]
**I0115**	Unetice_EBA	Germany	Central	U5a1i1	3954-3760	[49]
**I0116**	Unetice_EBA	Germany	Central	W3a1	4134-3939	[49]
**I0117**	Unetice_EBA	Germany	Central	I2'3	4272-4039	[49]
**I0164**	Unetice_EBA	Germany	Central	U5b2a1b	4023-3894	[49]
**I0803**	Unetice_EBA	Germany	Central	H4a1a1	4132-3942	[49]
**I0804**	Unetice_EBA	Germany	Central	H3+152	4137-3965	[49]
**Ust_Ishim**	Ust_Ishim_PHG	Russia	East	R	47530-42610	[57]
**I0231**	Yamna_CA	Russia	East	U4a1	4921-4762	[49]
**I0355**	Yamna_CA	Russia	East	K1b2a	5500-4700	[11]
**I0357**	Yamna_CA	Russia	East	W6c	5090-4913	[49]
**I0370**	Yamna_CA	Russia	East	H13a1a1	5300-4700	[49]
**I0429**	Yamna_CA	Russia	East	T2c1a2	5339-4918	[49]
**I0438**	Yamna_CA	Russia	East	U5a1a1	5021-4635	[49]
**I0439**	Yamna_CA	Russia	East	U5a1a1	5321-4921	[49]
**I0443**	Yamna_CA	Russia	East	W3a1a	5300-4700	[49]
**I0444**	Yamna_CA	Russia	East	H6a1b	5335-4882	[49]
**RISE547**	Yamna_CA	Russia	East	T2a1a	4887-4634	[48]
**RISE552**	Yamna_CA	Russia	East	T2a1a	4849-4143	[48]

The Geo_area feature refers to the scheme in figure 1 (main text). Haplogroups assigned might not completely correspond to those in the source paper, due to different NGS filters applied or different Tools used.

Table S7. Dataset of mtDNA ancient samples analyzed in the ABC analysis.

Sample	Analysis label	Country	Geo_area
**I0060**	Bell_Beaker_G_LN	Germany	Central
**I0108**	Bell_Beaker_G_LN	Germany	Central
**I0111**	Bell_Beaker_G_LN	Germany	Central
**I0112**	Bell_Beaker_G_LN	Germany	Central
**I0113**	Bell_Beaker_G_LN	Germany	Central
**I0805**	Bell_Beaker_G_LN	Germany	Central
**I0806**	Bell_Beaker_G_LN	Germany	Central
**I1530**	Bell_Beaker_G_LN	Germany	Central
**I1546**	Bell_Beaker_G_LN	Germany	Central
**I1549**	Bell_Beaker_G_LN	Germany	Central
**RISE564**	Bell_Beaker_G_LN	Germany	Central
**RISE00**	Corded_Ware_LN	Estonia	Central/East
**I0049**	Corded_Ware_LN	Germany	Central
**I0050**	Corded_Ware_LN	Germany	Central
**I0103**	Corded_Ware_LN	Germany	Central
**I0104**	Corded_Ware_LN	Germany	Central
**I0106**	Corded_Ware_LN	Germany	Central
**I1532**	Corded_Ware_LN	Germany	Central
**I1534**	Corded_Ware_LN	Germany	Central
**I1536**	Corded_Ware_LN	Germany	Central
**I1538**	Corded_Ware_LN	Germany	Central
**I1539**	Corded_Ware_LN	Germany	Central
**I1540**	Corded_Ware_LN	Germany	Central
**I1542**	Corded_Ware_LN	Germany	Central
**I1544**	Corded_Ware_LN	Germany	Central
**RISE435**	Corded_Ware_LN	Germany	Central
**RISE446**	Corded_Ware_LN	Germany	Central
**I2301**	Glob_Amph_MN	Poland	West
**I2403**	Glob_Amph_MN	Poland	West
**I2405**	Glob_Amph_MN	Poland	West
**I2433**	Glob_Amph_MN	Poland	West
**I2434**	Glob_Amph_MN	Poland	West
**I2440**	Glob_Amph_MN	Poland	West
**I2441**	Glob_Amph_MN	Poland	West
**I2801**	Glob_Amph_MN	Poland	West
**I2803**	Glob_Amph_MN	Poland	West
**I0232**	Srubnaya_EBA	Russia	East
**I0234**	Srubnaya_EBA	Russia	East
**I0235**	Srubnaya_EBA	Russia	East
**I0358**	Srubnaya_EBA	Russia	East
**I0359**	Srubnaya_EBA	Kazakhstan	East
**I0360**	Srubnaya_EBA	Russia	East
**I0361**	Srubnaya_EBA	Russia	East
**I0422**	Srubnaya_EBA	Russia	East
**I0424**	Srubnaya_EBA	Russia	East
**I0430**	Srubnaya_EBA	Russia	East
**I0431**	Srubnaya_EBA	Russia	East
**I0047**	Unetice_EBA	Russia	Central
**I0115**	Unetice_EBA	Hungary	Central
**I0116**	Unetice_EBA	Sweden	Central
**I0117**	Unetice_EBA	Sweden	Central
**I0164**	Unetice_EBA	Sweden	Central
**I0803**	Unetice_EBA	Sweden	Central
**I0804**	Unetice_EBA	Sweden	Central
**I0231**	Yamna_CA	Sweden	East
**I0355**	Yamna_CA	Germany	East
**I0357**	Yamna_CA	Germany	East
**I0370**	Yamna_CA	Germany	East
**I0429**	Yamna_CA	Germany	East
**I0438**	Yamna_CA	Germany	East
**I0439**	Yamna_CA	Germany	East
**I0443**	Yamna_CA	Germany	East
**I0444**	Yamna_CA	Russia	East
**RISE547**	Yamna_CA	Russia	East
**RISE552**	Yamna_CA	Russia	East

The Geo_area feature refers to the scheme in figure 1.

Table S8 - Prior distributions of the parameters of the tested demographic models

Parameter	Shape	Lower boundary	Upper boundary
**P***	uniform	0.01	0.5
**ACE**	Log uniform	100	10,000
**AEE**	Log uniform	100	10,000
**ANE**	Log uniform	1,000	100,000
**CCE**	Log uniform	1,000	100,000
**CEE**	Log uniform	1,000	100,000
**CNE**	Log uniform	1,000	100,000
**mut****	Log uniform	3.45E-9	3.45E-8

*** all the admixture proportions have the same prior distributions**

**** mutation rate per year per nucleotide**

Prior distribution associated to models' parameters. P is the admixture proportion; ACE, AEE and ANE are, respectively, the ancient effective population sizes of Central Europe, Eastern Europe and Near East; CCE, CEE and CNE are, respectively, the current effective population sizes of Central Europe, Eastern Europe and Near East.

Table S9 - Votes assigned to each model by Random Forests procedure and posterior probability for the selected model (comparison among 4 models).

Model selected	votes NOMIG	votes MIG1	votes MIG2	votes MIG1,2	PP
MIG2	44	107	180	169	0.40

Table S10 - Random forests confusion matrix and classification error (comparison among 4 models).

	NOMIG	MIG1	MIG2	MIG1,2	Classification error
**NOMIG**	32535	13892	9333	4393	0.46
**MIG1**	8902	18075	5419	9972	0.57
**MIG2**	5931	7396	23347	12355	0.52
**MIG1,2**	2632	10637	11901	23280	0.52

Table S11 - Random forests confusion matrix and classification error (comparison between MIG2 and MIG1,2 models).

	MIG2	MIG1,2	Classification error
**MIG2**	33352	17284	0.34
**MIG1,2**	16648	32716	0.34

Table S12 - Votes assigned to each model by Random Forest procedure and posterior probability for the selected model (comparison between MIG2 and MIG1,2 models).

Model selected	votes MIG2	votes MIG1,2	PP
MIG2	265	235	0.62

Table S13 - Parameter estimation for Model MIG2

	Median	Mode	95% HPD-LowB	95%HPD-UppB	R Squared
**P_{CW}**	0.33	0.43	0.07	0.5	0.28
**ACE**	5,327	6,048	1,388	9,836	0.57
**AEE**	6,843	8,080	3,130	10,000	0.60
**ANE**	1,557	1,000	1,000	4,643	0.49
**CCE**	30,808	12,841	1,000	89,139	0.12
**CEE**	33,145	14,822	2,960	92,931	0.12
**CNE**	15,315	1,000	1,000	84,802	0.12
**mut****	1.34E-08	1.37E-08	6.00E-09	2.11E-08	0.51

** mutation rate per year per nucleotide

Parameter estimation for Model MIG2. P_{CW} is the proportion of Corded Ware lineages coming from Yamna deme; ACE, AEE and ANE are, respectively, the ancient effective population sizes of Central Europe, Eastern Europe and Near East; CCE, CEE and CNE are, respectively, the current effective population sizes of Central Europe, Eastern Europe and Near East.

Table S14 - Votes assigned to each model by Random Forest procedure and posterior probability for the selected model (comparison between MIG2 and MIG2,3 models).

MIG2,3	225	275	0.53

Table S15 - Random forests confusion matrix and classification error (comparison between MIG2 and MIG2,3 models).

	MIG2	MIG2,3	Classification error
**MIG2**	37863	18346	0.33
**MIG2,3**	12137	31654	0.28

Table S16 - Parameter estimation for Model MIG2,3

	Median	Mode	95% HPD-LowB	95%HPD-UppB	R Squared
**P_{CW}**	0.31	0.42	0.04	0.49	0.27
**P_{BM}**	0.27	0.5	0.04	0.5	0.03
**ACE**	5,370	5,458	1,556	10,000	0.56
**AEE**	6,236	6,983	2,460	10,000	0.56
**ANE**	1,509	1,000	1,000	4,168	0.49
**CCE**	30,523	12,891	1,000	89,900	0.12
**CEE**	32,643	14,267	1,544	91,198	0.11
**CNE**	14,296	1,000	1,000	81,831	0.11
**mut****	1.38E-08	1.37E-08	6.14E-09	2.17E-08	0.50

** mutation rate per year per nucleotide

Parameter estimation for Model MIG2,3. P_{CW} is the proportion of Corded Ware lineages coming from Yamna deme; PBM is the proportion of Srubnaya lineages coming from a back migration after the onset of the Corded Ware culture; ACE, AEE and ANE are, respectively, the ancient effective population sizes of Central Europe, Eastern Europe and Near East; CCE, CEE and CNE are, respectively, the current effective population sizes of Central Europe, Eastern Europe and Near East.

Appendix

Input files for the demographic models

Fastsimcoal2 [38] requires two input files: the first input file is a template file (*.tpl) specifying the sample sizes, the sampling times, as well as the demographic model. The second input file specifies search ranges for the parameters to be estimated (*.est).

In the following, we provide the input files for all the models simulated.

MODEL NOMIG

Template file (.tpl)

```
//Parameters  for the coalescence simulation program : simcoal.exe
9 samples to simulate
//Deme sizes (haploid number of genes)
CCE
0
0
0
0
CNE
CEE
0
0
//Sample sizes
0
7 121
11 131
16 138
9 152
0
0
11 128
11 148
//Growth rates
GCE
0
0
0
0
GNE
GEE
0
0
//Number of migration matrices : If 0 : No migration between demes
0
//Historical event: time, source, sink, migrants, new deme size, new growth rate, new migration
matrix
14 historical events
121 1 0 1 1 GCE 0
128 7 6 1 1 GEE 0
131 2 0 1 1 GCE 0
138 3 0 1 1 GCE 0
148 8 6 1 1 GEE 0
152 4 0 1 1 GCE 0
293 0 0 1 1 0 0
294 0 5 0.4 1 GNE 0
295 6 6 1 1 0 0
296 6 5 0.4 1 GNE 0
345 5 5 1 1 0 0
1724 0 5 1 1 0 0
1725 6 5 1 1 0 0
1726 5 5 1 1 0 0
//Number of independent chromosome
```

```
1 1
//Number of contiguous linkage blocks
1
//Per Block: Data type, No. of loci, Recombination rate to the right-side locus, plus optional parameters
DNA 8399 0 mut 0.9375
```

Estimation file (.est)

```
// Priors and rules file
// *********************
[PARAMETERS]
//#isInt? #name        #dist.#min     #max
//all N are in number of diploid individuals
0      LOG_CCE unif    3      5      hide
0      LOG_CNE unif    3      5      hide
0      LOG_CEE unif    3      5      hide
0      LOG_ACE unif    2      4      hide
0      LOG_ANE unif    3      5      hide
0      LOG_AEE unif    2      4      hide
0      LOG_mut unif    -7     -6     hide
 [RULES]
LOG_ACE < LOG_CCE
LOG_ANE < LOG_CNE
LOG_AEE < LOG_CEE
[COMPLEX PARAMETERS]
1      CCE = pow10( LOG_CCE ) output
1      CNE = pow10( LOG_CNE ) output
1      CEE = pow10( LOG_CEE ) output
1      ACE = pow10( LOG_ACE ) output
1      ANE = pow10( LOG_ANE ) output
1      AEE = pow10( LOG_AEE ) output
0      mut = pow10(LOG_mut)  output
0      R0 = ACE/CCE   hide
0      L0 = log(R0)   hide
0      GCE = L0/293   output
0      R1 = ANE/CNE   hide
0      L1 = log(R1)   hide
0      GNE = L1/345   output
0      R2 = AEE/CEE   hide
0      L2 = log(R2)   hide
0      GEE = L2/295   output
```

MODEL MIG1

Template file (.tpl)

```
//Parameters  for the coalescence simulation program : simcoal.exe
9 samples to simulate
//Deme sizes (haploid number of genes)
CCE
0
0
0
0
CNE
CEE
0
0
//Sample sizes
0
7 121
11 131
16 138
9 152
0
0
11 128
11 148
//Growth rates
GCE
0
0
0
0
```

```
GNE
GEE
0
0
//Number of migration matrices : If 0 : No migration between demes
0
//Historical event: time, source, sink, migrants, new deme size, new growth rate, new migration matrix
15 historical events
121 1 0 1 1 GCE 0
128 7 6 1 1 GEE 0
131 2 0 1 1 GCE 0
138 3 0 1 1 GCE 0
148 8 6 1 1 GEE 0
152 4 0 1 1 GCE 0
TADM1 0 6 P1 1 GEE 0
293 0 0 1 1 0 0
294 0 5 0.4 1 GNE 0
295 6 6 1 1 0 0
296 6 5 0.4 1 GNE 0
345 5 5 1 1 0 0
1724 0 5 1 1 0 0
1725 6 5 1 1 0 0
1726 5 5 1 1 0 0
//Number of independent chromosome
1 1
//Number of contiguous linkage blocks
1
//Per Block: Data type, No. of loci, Recombination rate to the right-side locus, plus optional parameters
DNA 8399 0 mut 0.9375
```

Estimation file (.est)

```
// Priors and rules file
// *********************
[PARAMETERS]
//#isInt? #name       #dist.#min     #max
//all N are in number of diploid individuals
0       LOG_CCE unif    3       5       hide
0       LOG_CNE unif    3       5       hide
0       LOG_CEE unif    3       5       hide
0       LOG_ACE unif    2       4       hide
0       LOG_ANE unif    3       5       hide
0       LOG_AEE unif    2       4       hide
0       LOG_mut unif    -7      -6      hide
1       TADM1   unif    172     207     output
0       P1      unif    0.01    0.5     output
[RULES]
LOG_ACE < LOG_CCE
LOG_ANE < LOG_CNE
LOG_AEE < LOG_CEE
[COMPLEX PARAMETERS]
1       CCE = pow10( LOG_CCE ) output
1       CNE = pow10( LOG_CNE ) output
1       CEE = pow10( LOG_CEE ) output
1       ACE = pow10( LOG_ACE ) output
1       ANE = pow10( LOG_ANE ) output
1       AEE = pow10( LOG_AEE ) output
0       mut = pow10(LOG_mut)   output
0       R0 = ACE/CCE   hide
0       L0 = log(R0)   hide
0       GCE = L0/293   output
0       R1 = ANE/CNE   hide
0       L1 = log(R1)   hide
0       GNE = L1/345   output
0       R2 = AEE/CEE   hide
0       L2 = log(R2)   hide
0       GEE = L2/295   output
```

MODEL MIG2

Template file (.tpl)

```
//Parameters  for the coalescence simulation program : simcoal.exe
9 samples to simulate
//Deme sizes (haploid number of genes)
CCE
0
```

```
0
0
0
CNE
CEE
0
0
//Sample sizes
0
7 121
11 131
16 138
9 152
0
0
11 128
11 148
//Growth rates
GCE
0
0
0
0
GNE
GEE
0
0
//Number of migration matrices : If 0 : No migration between demes
0
//Historical event: time, source, sink, migrants, new deme size, new growth rate, new migration matrix
15 historical events
121 1 0 1 1 GCE 0
128 7 6 1 1 GEE 0
131 2 0 1 1 GCE 0
138 3 0 1 1 GCE 0
TADM2 0 6 P2 1 GEE 0
148 8 6 1 1 GEE 0
152 4 0 1 1 GCE 0
293 0 0 1 1 0 0
294 0 5 0.4 1 GNE 0
295 6 6 1 1 0 0
296 6 5 0.4 1 GNE 0
345 5 5 1 1 0 0
1724 0 5 1 1 0 0
1725 6 5 1 1 0 0
1726 5 5 1 1 0 0
//Number of independent chromosome
1 1
//Number of contiguous linkage blocks
1
//Per Block: Data type, No. of loci, Recombination rate to the right-side locus, plus optional parameters
DNA 8399 0 mut 0.9375
```

Estimation file (.est)

```
// Priors and rules file
// *********************
[PARAMETERS]
//#isInt? #name	#dist.#min	#max
//all N are in number of diploid individuals
0	LOG_CCE	unif	3	5	hide
0	LOG_CNE	unif	3	5	hide
0	LOG_CEE	unif	3	5	hide
0	LOG_ACE	unif	2	4	hide
0	LOG_ANE	unif	3	5	hide
0	LOG_AEE	unif	2	4	hide
0	LOG_mut	unif	-7	-6	hide
1	TADM2	unif	139	147	output
0	P2	unif	0.01	0.5	output
```

```
[RULES]
LOG_ACE < LOG_CCE
LOG_ANE < LOG_CNE
LOG_AEE < LOG_CEE
[COMPLEX PARAMETERS]
1      CCE = pow10( LOG_CCE ) output
1      CNE = pow10( LOG_CNE ) output
1      CEE = pow10( LOG_CEE ) output
1      ACE = pow10( LOG_ACE ) output
1      ANE = pow10( LOG_ANE ) output
1      AEE = pow10( LOG_AEE ) output
0      mut = pow10(LOG_mut)   output
0      R0 = ACE/CCE   hide
0      L0 = log(R0)   hide
0      GCE = L0/293   output
0      R1 = ANE/CNE   hide
0      L1 = log(R1)   hide
0      GNE = L1/345   output
0      R2 = AEE/CEE   hide
0      L2 = log(R2)   hide
0      GEE = L2/295   output
```

MODEL MIG1,2

Template file (.tpl)

```
//Parameters  for the coalescence simulation program : simcoal.exe
9 samples to simulate
//Deme sizes (haploid number of genes)
CCE
0
0
0
0
CNE
CEE
0
0
//Sample sizes
0
7 121
11 131
16 138
9 152
0
0
11 128
11 148
//Growth rates
GCE
0
0
0
0
GNE
GEE
0
0
//Number of migration matrices : If 0 : No migration between demes
0
//Historical event: time, source, sink, migrants, new deme size, new growth rate, new migration
matrix
16 historical events
121 1 0 1 1 GCE 0
128 7 6 1 1 GEE 0
131 2 0 1 1 GCE 0
138 3 0 1 1 GCE 0
TADM2 0 6 P2 1 GEE 0
```

```
148 8 6 1 1 GEE 0
152 4 0 1 1 GCE 0
TADM1 0 6 P1 1 GEE 0
293 0 0 1 1 0 0
294 0 5 0.4 1 GNE 0
295 6 6 1 1 0 0
296 6 5 0.4 1 GNE 0
345 5 5 1 1 0 0
1724 0 5 1 1 0 0
1725 6 5 1 1 0 0
1726 5 5 1 1 0 0
//Number of independent chromosome
1 1
//Number of contiguous linkage blocks
1
//Per Block: Data type, No. of loci, Recombination rate to the right-side locus, plus optional
parameters
DNA 8399 0 mut 0.9375
```

Estimation file (.est)

```
// Priors and rules file
// *********************
[PARAMETERS]
//#isInt? #name       #dist.#min     #max
//all N are in number of diploid individuals
0       LOG_CCE unif    3       5       hide
0       LOG_CNE unif    3       5       hide
0       LOG_CEE unif    3       5       hide
0       LOG_ACE unif    2       4       hide
0       LOG_ANE unif    3       5       hide
0       LOG_AEE unif    2       4       hide
0       LOG_mut unif    -7      -6      hide
1       TADM2   unif    139     147     output
0       P2      unif    0.01    0.5     output
1       TADM1   unif    172     207     output
0       P1      unif    0.01    0.5     output
[RULES]
LOG_ACE < LOG_CCE
LOG_ANE < LOG_CNE
LOG_AEE < LOG_CEE
[COMPLEX PARAMETERS]
1       CCE = pow10( LOG_CCE ) output
1       CNE = pow10( LOG_CNE ) output
1       CEE = pow10( LOG_CEE ) output
1       ACE = pow10( LOG_ACE ) output
1       ANE = pow10( LOG_ANE ) output
1       AEE = pow10( LOG_AEE ) output
0       mut = pow10(LOG_mut)   output
0       R0 = ACE/CCE    hide
0       L0 = log(R0)    hide
0       GCE = L0/293    output
0       R1 = ANE/CNE    hide
0       L1 = log(R1)    hide
0       GNE = L1/345    output
0       R2 = AEE/CEE    hide
0       L2 = log(R2)    hide
0       GEE = L2/295    output
```

MODEL MIG2,3

Template file (.tpl)

```
//Parameters  for the coalescence simulation program : simcoal.exe
9 samples to simulate
//Deme sizes (haploid number of genes)
CCE
0
0
0
0
```

```
CNE
CEE
0
0
//Sample sizes
0
7 121
11 131
16 138
9 152
0
0
11 128
11 148
//Growth rates
GCE
0
0
0
0
GNE
GEE
0
0
//Number of migration matrices : If 0 : No migration between demes
0
//Historical event: time, source, sink, migrants, new deme size, new growth rate, new migration
matrix
16 historical events
121 1 0 1 1 GCE 0
128 7 6 1 1 GEE 0
131 2 0 1 1 GCE 0
TBM 6 0 Pbm 1 GCE 0
138 3 0 1 1 GCE 0
TADM2 0 6 P2 1 GEE 0
148 8 6 1 1 GEE 0
152 4 0 1 1 GCE 0
293 0 0 1 1 0 0
294 0 5 0.4 1 GNE 0
295 6 6 1 1 0 0
296 6 5 0.4 1 GNE 0
345 5 5 1 1 0 0
1724 0 5 1 1 0 0
1725 6 5 1 1 0 0
1726 5 5 1 1 0 0
//Number of independent chromosome
1 1
//Number of contiguous linkage blocks
1
//Per Block: Data type, No. of loci, Recombination rate to the right-side locus, plus optional
parameters
DNA 8399 0 mut 0.9375
```

Estimation file (.est)

```
// Priors and rules file
// *********************
[PARAMETERS]
//#isInt? #name       #dist.#min     #max
//all N are in number of diploid individuals
0       LOG_CCE unif    3       5       hide
0       LOG_CNE unif    3       5       hide
0       LOG_CEE unif    3       5       hide
0       LOG_ACE unif    2       4       hide
0       LOG_ANE unif    3       5       hide
0       LOG_AEE unif    2       4       hide
0       LOG_mut unif    -7      -6      hide
1       TBM     unif    132     137     output
0       Pbm     unif    0.01    0.5     output
1       TADM2   unif    139     147     output
0       P2      unif    0.01    0.5     output
```

```
[RULES]
LOG_ACE < LOG_CCE
LOG_ANE < LOG_CNE
LOG_AEE < LOG_CEE
[COMPLEX PARAMETERS]
1       CCE = pow10( LOG_CCE ) output
1       CNE = pow10( LOG_CNE ) output
1       CEE = pow10( LOG_CEE ) output
1       ACE = pow10( LOG_ACE ) output
1       ANE = pow10( LOG_ANE ) output
1       AEE = pow10( LOG_AEE ) output
0       mut = pow10(LOG_mut)   output
0       R0 = ACE/CCE   hide
0       L0 = log(R0)   hide
0       GCE = L0/293   output
0       R1 = ANE/CNE   hide
0       L1 = log(R1)   hide
0       GNE = L1/345   output
0       R2 = AEE/CEE   hide
0       L2 = log(R2)   hide
0       GEE = L2/295   output
```

Supplementary References

1. Gimbutas M. 1979 The three waves of the Kurgan people into old Europe. *Archives Suisses d'Anthropologie Génerale* **43**, 113-117.

2. Wiślański T. 1966 *Kultura amfor kulistych w Polsce północno-zachodniej*. Poznań.

3. Godłowski K., Kozłowski J. 1976 *Historia starożytna ziem polskich. Państwowe Wydawnictwo Naukowe*. Warszawa.

4. Gąssowski J. 1985 *Kultura pradziejowa na ziemiach Polski. Państwowe Wydawnictwo Naukowe*. Warszawa.

5. Ciesielska A. 2011 *Społeczeństwa Europy pradziejowej. Wydawnictwo Naukowe UAM*. Poznań.

6. Budnik A., Wrzesiński J. 2002 Kierzkowo - między inhumacją a ciałopaleniem. In *Wrzesiński J* (ed. Popiół i Kość. Sobótka W.M.Ś.i.S.D.w.S., AKME Zdzisław Wiśniewsk), pp. 125-145.

7. Bakker J. 1992 *he Ditch Hunebedden. Megalithic tombs of the Funnel Beaker culture*. Michigan.

8. Mathieson I., Alpaslan R., S, Posth C., Szécsényi-Nagy A., Rohland A., Mallick S., Olalde I., Broomandkhoshbacht I., Cheronet O., Fernandes D., et al. 2017 The Genomic History Of Southeastern Europe. *Preprint at bioRxiv*. (doi:https://doi.org/10.1101/135616).

9. Alexander D.H., Novembre J., Lange K. 2009 Fast model-based estimation of ancestry in unrelated individuals. *Genome Res* 19(9), 1655-1664. (doi:10.1101/gr.094052.109).

10. Purcell S., Neale B., Todd-Brown K., Thomas L., Ferreira M.A., Bender D., Maller J., Sklar P., de Bakker P.I., Daly M.J., et al. 2007 PLINK: a tool set for whole-genome association and population-based linkage analyses. Am J Hum Genet 81(3), 559-575. (doi:10.1086/519795).

11. Haak W., Lazaridis I., Patterson N., Rohland N., Mallick S., Llamas B., Brandt G., Nordenfelt S., Harney E., Stewardson K., et al. 2015 Massive migration from the steppe was a source for Indo-European languages in Europe. *Nature* **522**(7555), 207-211. (doi:10.1038/nature14317).

12. Jakobsson M., Rosenberg N.A. 2007 CLUMPP: a cluster matching and permutation program for dealing with label switching and multimodality in analysis of population structure. *Bioinformatics* 23(14), 1801-1806. (doi:10.1093/bioinformatics/btm233).

13. Rosenberg N.A. 2004 Distruct: a program for the graphical display of population *Molecular Ecology Notes* 4, 137-138.

14. Patterson N., Moorjani P., Luo Y., Mallick S., Rohland N., Zhan Y., Genschoreck T., Webster T., Reich D. 2012 Ancient admixture in human history. Genetics 192(3), 1065-1093. (doi:10.1534/genetics.112.145037).

15. Busing FMTA, Meijer E, R. V.D.L. 1999 Delete- m Jackknife for Unequal m. *Stat Comput* 9, 3-8.

16. Pickrell J.K., Pritchard J.K. 2012 Inference of population splits and mixtures from genome-wide allele frequency data. PLoS Genet 8(11), e1002967. (doi:10.1371/journal.pgen.1002967).

17. Petkova D., Novembre J., Stephens M. 2016 Visualizing spatial population structure with estimated effective migration surfaces. Nat Genet 48(1), 94-100. (doi:10.1038/ng.3464).

18. Dabney J., Knapp M., Glocke I., Gansauge M.T., Weihmann A., Nickel B., Valdiosera C., Garcia N., Paabo S., Arsuaga J.L., et al. 2013 Complete mitochondrial genome sequence of a Middle Pleistocene cave bear reconstructed from ultrashort DNA fragments. *Proc Natl Acad Sci U S A* **110**(39), 15758-15763. (doi:10.1073/pnas.1314445110).

19. Modi A., Tassi F., Susca R.R., Vai S., Rizzi E., Bellis G., Luglie C., Gonzalez Fortes G., Lari M., Barbujani G., et al. 2017 Complete mitochondrial sequences from Mesolithic Sardinia. *Sci Rep* **7**, 42869. (doi:10.1038/srep42869).

20. Meyer M., Kircher M. 2010 Illumina sequencing library preparation for highly multiplexed target capture and sequencing. *Cold Spring Harb Protoc* **2010**(6), pdb prot5448. (doi:10.1101/pdb.prot5448).

21. Rohland N., Harney E., Mallick S., Nordenfelt S., Reich D. 2015 Partial uracil-DNA-glycosylase treatment for screening of ancient DNA. *Philos Trans R Soc Lond B Biol Sci* **370**(1660), 20130624. (doi:10.1098/rstb.2013.0624).

22. Maricic T., Whitten M., Paabo S. 2010 Multiplexed DNA sequence capture of mitochondrial genomes using PCR products. *PLoS One* **5**(11), e14004. (doi:10.1371/journal.pone.0014004).

23. Andrews R.M., Kubacka I., Chinnery P.F., Lightowlers R.N., Turnbull D.M., Howell N. 1999 Reanalysis and revision of the Cambridge reference sequence for human mitochondrial DNA. *Nat Genet* 23(2), 147. (doi:10.1038/13779).

24. Li H., Durbin R. 2009 Fast and accurate short read alignment with Burrows-Wheeler transform. *Bioinformatics* **25**(14), 1754-1760. (doi:10.1093/bioinformatics/btp324).

25. Jonsson H., Ginolhac A., Schubert M., Johnson P.L., Orlando L. 2013 mapDamage2.0: fast approximate Bayesian estimates of ancient DNA damage parameters. *Bioinformatics* **29**(13), 1682-1684. (doi:10.1093/bioinformatics/btt193).

26. Fu Q., Mittnik A., Johnson P.L., Bos K., Lari M., Bollongino R., Sun C., Giemsch L., Schmitz R., Burger J., et al. 2013 A revised timescale for human evolution based on ancient mitochondrial genomes. *Curr Biol* **23**(7), 553-559. (doi:10.1016/j.cub.2013.02.044).

27. Milne I., Bayer M., Cardle L., Shaw P., Stephen G., Wright F., Marshall D. 2010 Tablet--next generation sequence assembly visualization. *Bioinformatics* 26(3), 401-402. (doi:10.1093/bioinformatics/btp666).

28. Sawyer S., Krause J., Guschanski K., Savolainen V., Paabo S. 2012 Temporal patterns of nucleotide misincorporations and DNA fragmentation in ancient DNA. *PLoS One* **7**(3), e34131. (doi:10.1371/journal.pone.0034131).

29. Edgar R.C. 2004 MUSCLE: multiple sequence alignment with high accuracy and high throughput. *Nucleic Acids Res* 32(5), 1792-1797. (doi:10.1093/nar/gkh340).

30. van Oven M., Kayser M. 2009 Updated comprehensive phylogenetic tree of global human mitochondrial DNA variation. *Hum Mutat* **30**(2), E386-394. (doi:10.1002/humu.20921).

31. Kloss-Brandstatter A., Pacher D., Schonherr S., Weissensteiner H., Binna R., Specht G., Kronenberg F. 2011 HaploGrep: a fast and reliable algorithm for automatic classification of mitochondrial DNA haplogroups. *Hum Mutat* **32**(1), 25-32. (doi:10.1002/humu.21382).

32. Excoffier L., Lischer H.E. 2010 Arlequin suite ver 3.5: a new series of programs to perform population genetics analyses under Linux and Windows. *Mol Ecol Resour* 10(3), 564-567. (doi:10.1111/j.1755-0998.2010.02847.x).

33. R Development Core Team. 2011 R: A Language and Environment for Statistical Computing. Vienna, Austria : the R Foundation for Statistical Computing. *ISBN: 3-900051-07-0 Available online at http://wwwR-projectorg/*.

34 Paradis E., Claude J., Strimmer K. 2004 APE: Analyses of Phylogenetics and Evolution in R language. *Bioinformatics* **20**(2), 289-290.

35 Bandelt H.J., Forster P., Sykes B.C., Richards M.B. 1995 Mitochondrial portraits of human populations using median networks. Genetics **141**(2), 743-753.

36. Breiman L. 2001 Random forests. *Mach Learn* **45**, 5-32.

37. Pudlo P., Marin J.M., Estoup A., Cornuet J.M., Gautier M., Robert C.P. 2016 Reliable ABC model choice via random forests. *Bioinformatics* **32**(6), 859-866. (doi:10.1093/bioinformatics/btv684).

38. Excoffier L., Dupanloup I., Huerta-Sánchez E., Sousa V.C., Foll M. 2013 Robust demographic inference from genomic and SNP data. *PLOS Genetics* **9**(10):e1003905.

39. Wegmann D., Leuenberger C., Neuenschwander S., Excoffier, L. 2010. ABCtoolbox: a versatile toolkit for approximate Bayesian computations. *BMC Bioinformatics* **11**, 116. Available at: http://www.biomedcentral.com/1471-2105/11/116.

40. Skoglund P., Malmstrom H., Raghavan M., Stora J., Hall P., Willerslev E., Gilbert M.T., Gotherstrom A., Jakobsson M. 2012 Origins and genetic legacy of Neolithic farmers and hunter-gatherers in Europe. *Science* **336**(6080), 466-469. (doi:10.1126/science.1216304).

41. Lipson M., Szécsényi-Nagy A., Mallick S., Pósa A., Stégmár B., Keerl V., Rohland N., Stewardson K., Ferry M., Michel M., et al. 2017 Parallel ancient genomic transects reveal complex population history of early European farmers. *Preprint at bioRxiv*. (doi:doi: https://doi.org/10.1101/114488).

42. Excoffier L., Lischer H.E. 2010 Arlequin suite ver 3.5: a new series of programs to perform population genetics analyses under Linux and Windows. *Mol Ecol Resour* **10**(3), 564-567. (doi:10.1111/j.1755-0998.2010.02847.x).

43. Beaumont M.A., Zhang W., Balding D.J. 2002 Approximate Bayesian computation in population genetics. *Genetics* **162**(4), 2025-2035.

44. Hamilton G., Stoneking M., Excoffier L. 2005 Molecular analysis reveals tighter social regulation of immigration in patrilocal populations than in matrilocal populations. *Proc Natl Acad Sci U S A* **102**(21), 7476-7480. (doi:10.1073/pnas.0409253102).

45. Wegmann D., Leuenberger C., Neuenschwander S., Excoffier L. 2010 ABCtoolbox: a versatile toolkit for approximate Bayesian computations. *BMC Bioinformatics* **11**, 116. (doi:10.1186/1471-2105-11-116).

46. QGIS Development Team. 2017 QGIS Geographic Information System. *Open Source Geospatial Foundation Project*.

47. Gamba C., Jones E.R., Teasdale M.D., McLaughlin R.L., Gonzalez-Fortes G., Mattiangeli V., Domboroczki L., Kovari I., Pap I., Anders A., et al. 2014 Genome flux and stasis in a five millennium transect of European prehistory. *Nat Commun* **5**, 5257. (doi:10.1038/ncomms6257).

48. Allentoft M.E., Sikora M., Sjogren K.G., Rasmussen S., Rasmussen M., Stenderup J., Damgaard P.B., Schroeder H., Ahlstrom T., Vinner L., et al. 2015 Population genomics of Bronze Age Eurasia. *Nature* **522**(7555), 167-172. (doi:10.1038/nature14507).

49. Mathieson I., Lazaridis I., Rohland N., Mallick S., Patterson N., Roodenberg S.A., Harney E., Stewardson K., Fernandes D., Novak M., et al. 2015 Genome-wide patterns of selection in 230 ancient Eurasians. *Nature* **528**(7583), 499-503. (doi:10.1038/nature16152).

50. Gunther T., Valdiosera C., Malmstrom H., Urena I., Rodriguez-Varela R., Sverrisdottir O.O., Daskalaki E.A., Skoglund P., Naidoo T., Svensson E.M., et al. 2015 Ancient genomes link early farmers from Atapuerca in Spain to modern-day Basques. *Proc Natl Acad Sci U S A* **112**(38), 11917-11922. (doi:10.1073/pnas.1509851112).

51. Skoglund P., Northoff B.H., Shunkov M.V., Derevianko A.P., Paabo S., Krause J., Jakobsson M. 2014 Separating endogenous ancient DNA from modern day contamination in a Siberian Neandertal. *Proc Natl Acad Sci U S A* **111**(6), 2229-2234. (doi:10.1073/pnas.1318934111).

52. Keller A., Graefen A., Ball M., Matzas M., Boisguerin V., Maixner F., Leidinger P., Backes C., Khairat R., Forster M., et al. 2012 New insights into the Tyrolean Iceman's origin and phenotype as inferred by whole-genome sequencing. *Nat Commun* **3**, 698. (doi:10.1038/ncomms1701).

53. Lazaridis I., Patterson N., Mittnik A., Renaud G., Mallick S., Kirsanow K., Sudmant P.H., Schraiber J.G., Castellano S., Lipson M., et al. 2014 Ancient human genomes suggest three ancestral populations for present-day Europeans. *Nature* **513**(7518), 409-413. (doi:10.1038/nature13673).

54. Olalde I., Allentoft M.E., Sanchez-Quinto F., Santpere G., Chiang C.W., DeGiorgio M., Prado-Martinez J., Rodriguez J.A., Rasmussen S., Quilez J., et al. 2014 Derived immune and ancestral pigmentation alleles in a 7,000-year-old Mesolithic European. *Nature* **507**(7491), 225-228. (doi:10.1038/nature12960).

55. Fu Q., Posth C., Hajdinjak M., Petr M., Mallick S., Fernandes D., Furtwangler A., Haak W., Meyer M., Mittnik A., et al. 2016 The genetic history of Ice Age Europe. *Nature* **534**(7606), 200-205. (doi:10.1038/nature17993).

56. Raghavan M., Skoglund P., Graf K.E., Metspalu M., Albrechtsen A., Moltke I., Rasmussen S., Stafford T.W., Jr., Orlando L., Metspalu E., et al. 2014 Upper Palaeolithic Siberian genome reveals dual ancestry of Native Americans. *Nature* **505**(7481), 87-91. (doi:10.1038/nature12736).

57. Fu Q., Li H., Moorjani P., Jay F., Slepchenko S.M., Bondarev A.A., Johnson P.L., Aximu-Petri A., Prufer K., de Filippo C., et al. 2014 Genome sequence of a 45,000-year-old modern human from western Siberia. *Nature* **514**(7523), 445-449. (doi:10.1038/nature13810).

58 Posth C., Renaud G., Mittnik A., Drucker D.G., Rougier H., Cupillard C., Valentin F., Thevenet C., Furtwangler A., Wissing C., et al. 2016 Pleistocene Mitochondrial Genomes Suggest a Single Major Dispersal of Non-Africans and a Late Glacial Population Turnover in Europe. *Curr Biol* **26**(6), 827-833. (doi:10.1016/j.cub.2016.01.037).

59. Ermini L., Olivieri C., Rizzi E., Corti G., Bonnal R., Soares P., Luciani S., Marota I., De Bellis G., Richards M.B., et al. 2008 Complete mitochondrial genome sequence of the Tyrolean Iceman. *Curr Biol* **18**(21), 1687-1693. (doi:10.1016/j.cub.2008.09.028).

60. Krause J., Briggs A.W., Kircher M., Maricic T., Zwyns N., Derevianko A., Paabo S. 2010 A complete mtDNA genome of an early modern human from Kostenki, Russia. *Curr Biol* **20**(3), 231-236. (doi:10.1016/j.cub.2009.11.068).
