# Supplementary material for: Genome diversity in the Neolithic Globular Amphorae culture and the spread of Indo-European languages
Source: Proc Biol Sci. 2017 Nov 22;284(1867):20171540. doi: 10.1098/rspb.2017.1540 (PMC5719168; doi:10.1098/rspb.2017.1540)

Supplementary figures

Figure S1 - Diagram of the Kurgan hypothesis, as revised by Marija Gimbutas.

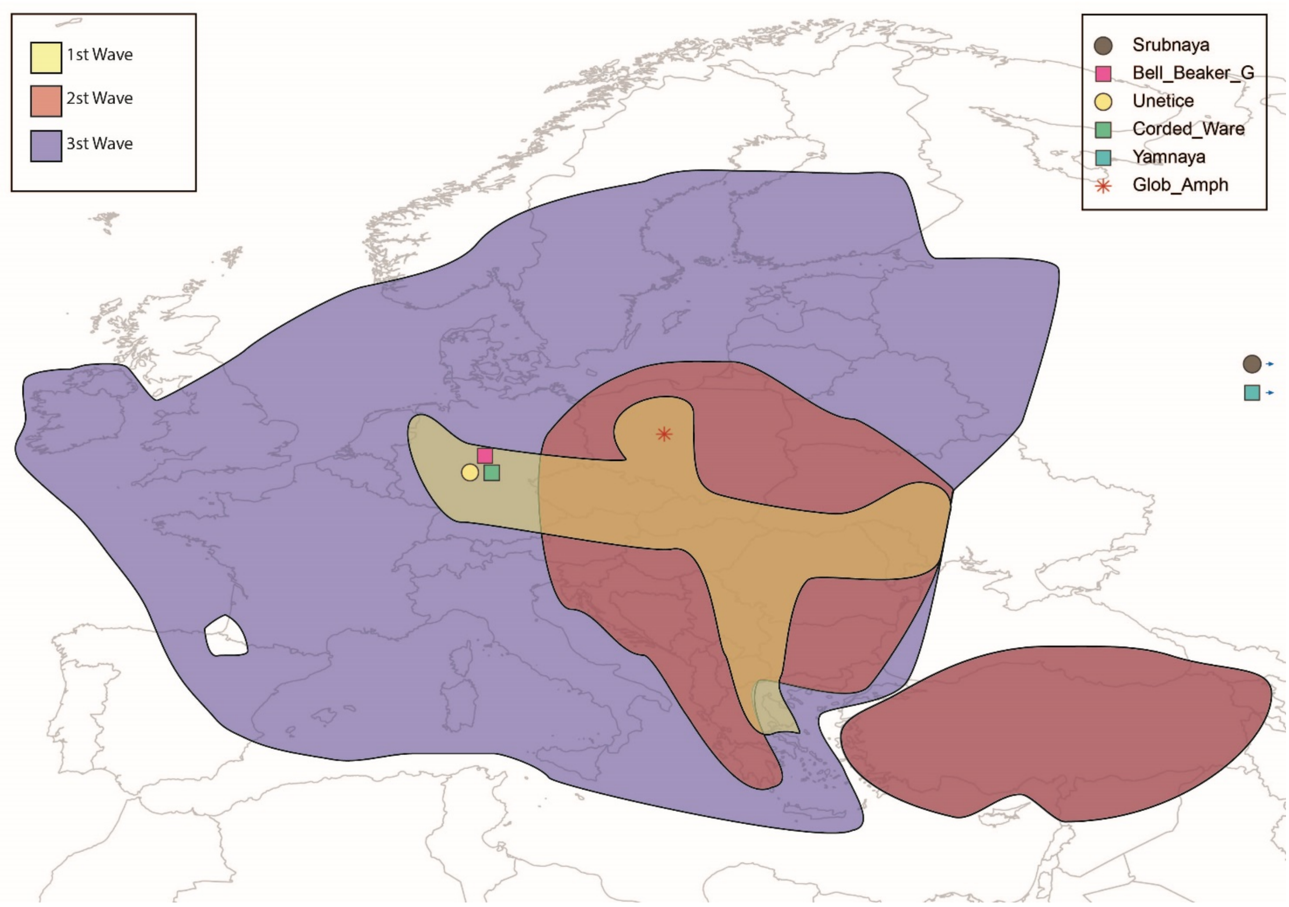

The background shading indicates the tree migratory waves proposed by Marija Gimbutas, and personally checked by her in 1995. The symbols refer to the ancient populations considered in the ABC analysis (supplementary table S7)

Figure S2 - Principal Component Analysis on the whole dataset.

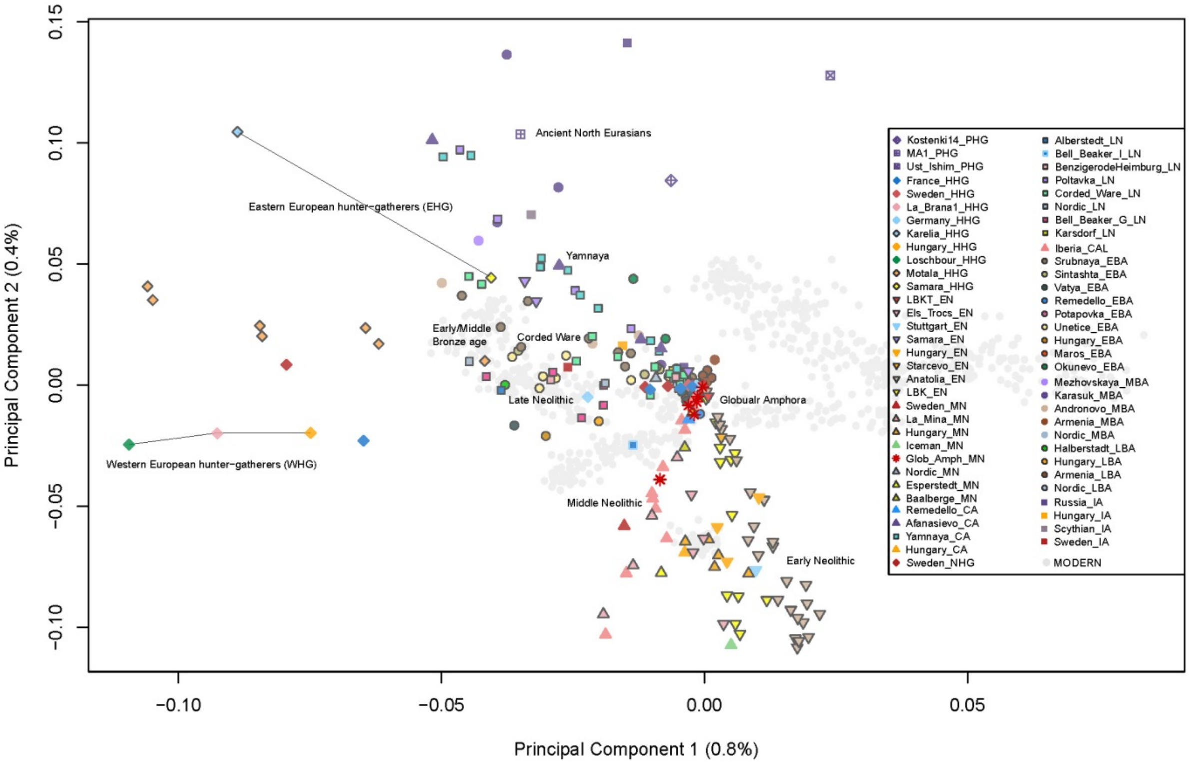

To analyse the Globular Amphorae individuals in the context of ancient and present-day genetic diversity, we merged them with: 249 ancient individuals (reported in table S2) and 777 West Eurasian individuals (table S3). The plot shows a PCA performed using only transversions (111,208 SNPs). Modern individuals are grey dots, colored and labeled symbols the ancient individuals (as reported in figure 1).We obtained the same general pattern found analysing the optimized dataset (see figure 3a). The newly reported GAC individuals fell within a cluster comprising most Early- and Middle Neolithic individuals. We recognize a clear separation between hunter-gathers and later samples, with the Bronze Age individuals at the top, the Late Neolithic samples in a central position and the Early and Middle Neolithic samples at the bottom.

Figure S3 - Distribution of the genotyped SNPs in the ancient populations.

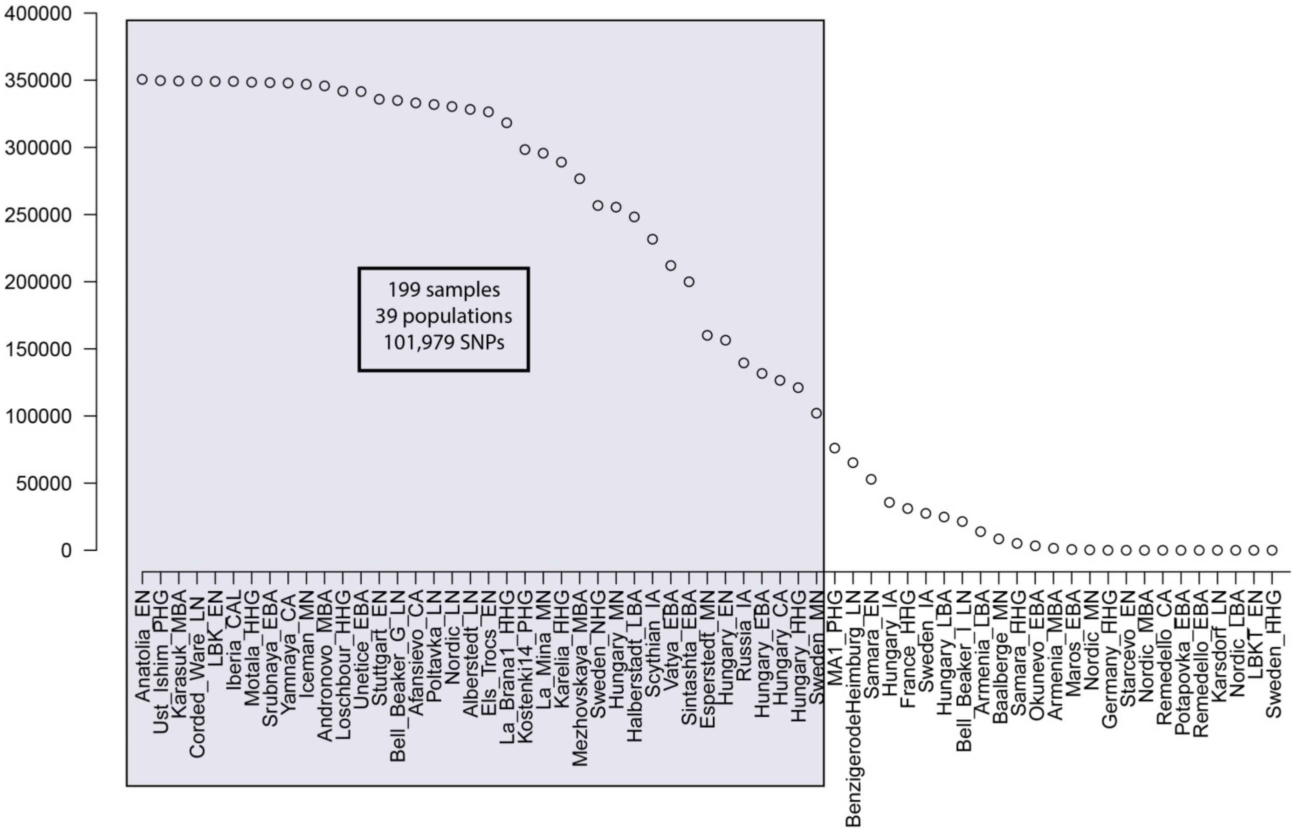

The circles represent the decreasing number of SNPs covered in at least one GA individual, considering the other ancient populations one by one. The purple inset highlights the subset used for all population genetic analyses, i.e. 199 samples belonging to 39 populations covered at 101,979 SNPs.

Figure S4 - Principal Component Analysis of the 199 ancient individuals included in *(A)* dataset

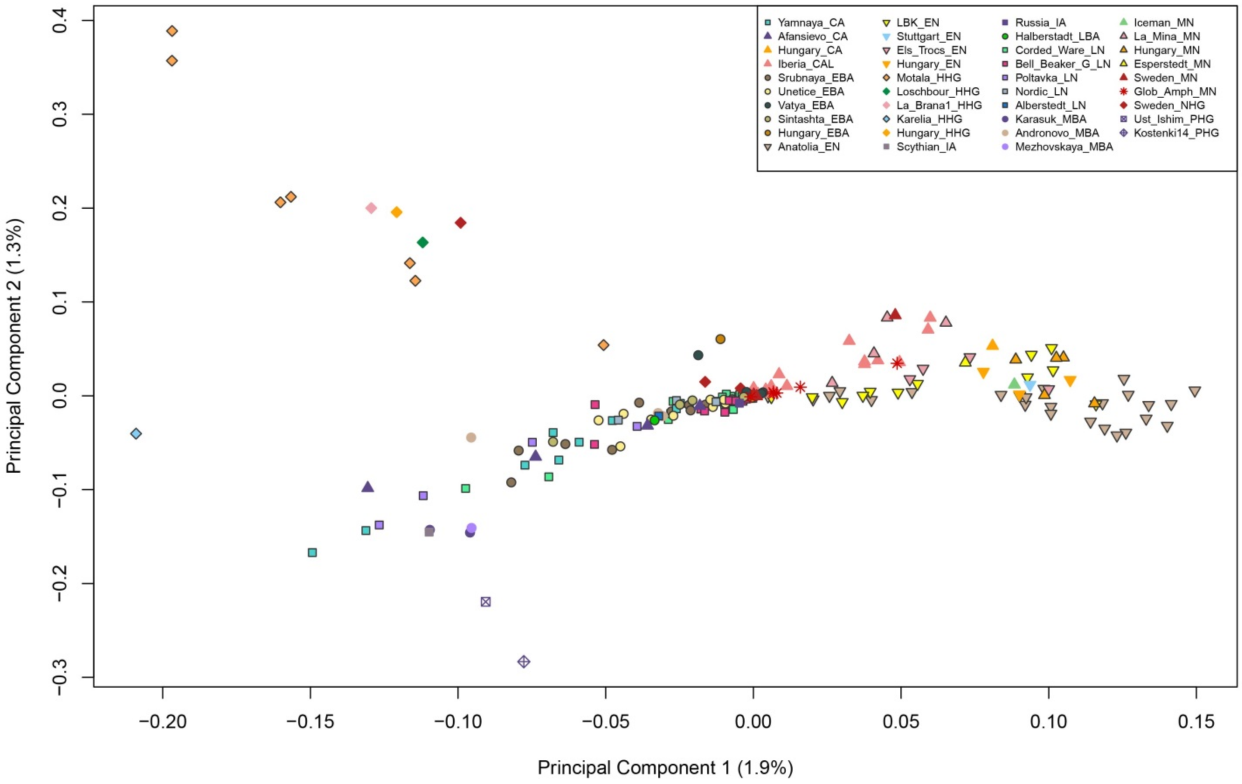

The PCA was performed using only on 18,198 transversions (to avoid confounding effects related to postmortem damage). We used the same coloured and labelled symbols to represent the ancient individuals reported in figure 1.

Figure S5 - Complement of Principal Component Analysis in figure 3a.

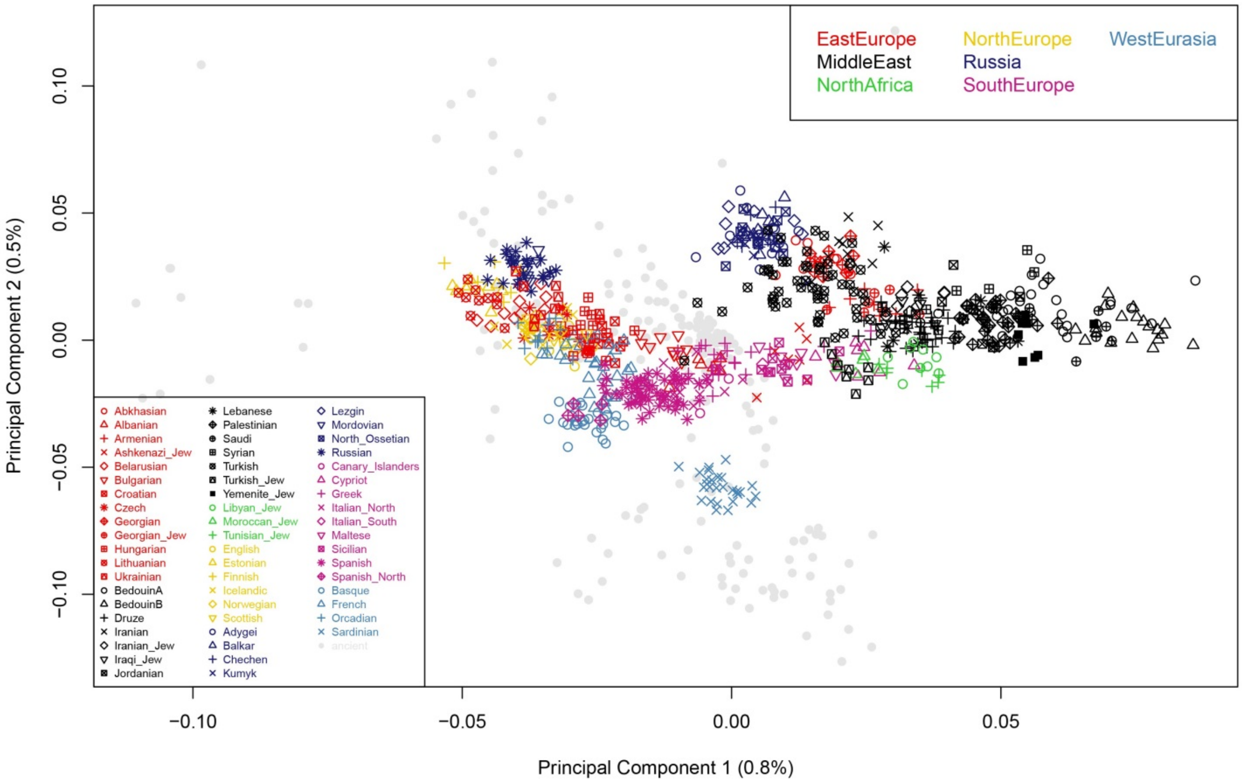

The PCA analysis is performed on the same set of individual reported in figure 3a. Modern individuals are coloured by geographical region (instead of using grey points as in figure 3a) and the shape of the symbols indicates the population grouping. The ancient individuals are shown as grey dots.

Figure S6 - Multi-dimensional scaling (MDS) analysis.

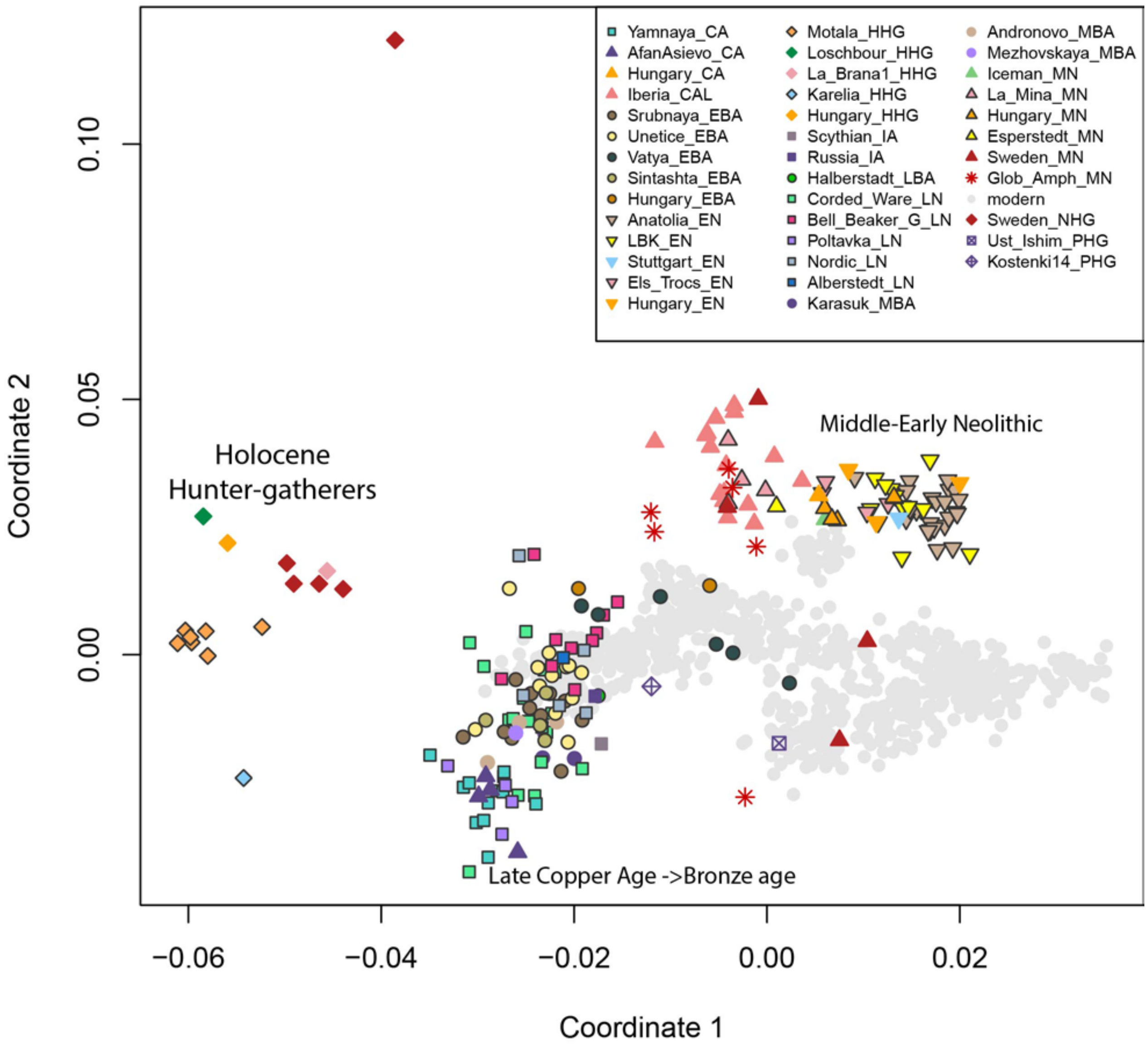

The MDS is based on a matrix of genetic distances between pairs of individuals in the dataset *(AP)*, taking into account for each pair only the shared non-missing genotypes. We represented modern individuals as grey dots, and used coloured and labelled symbols to represent the ancient individuals

Figure S7 - Admixture analysis based only on ancient variation.

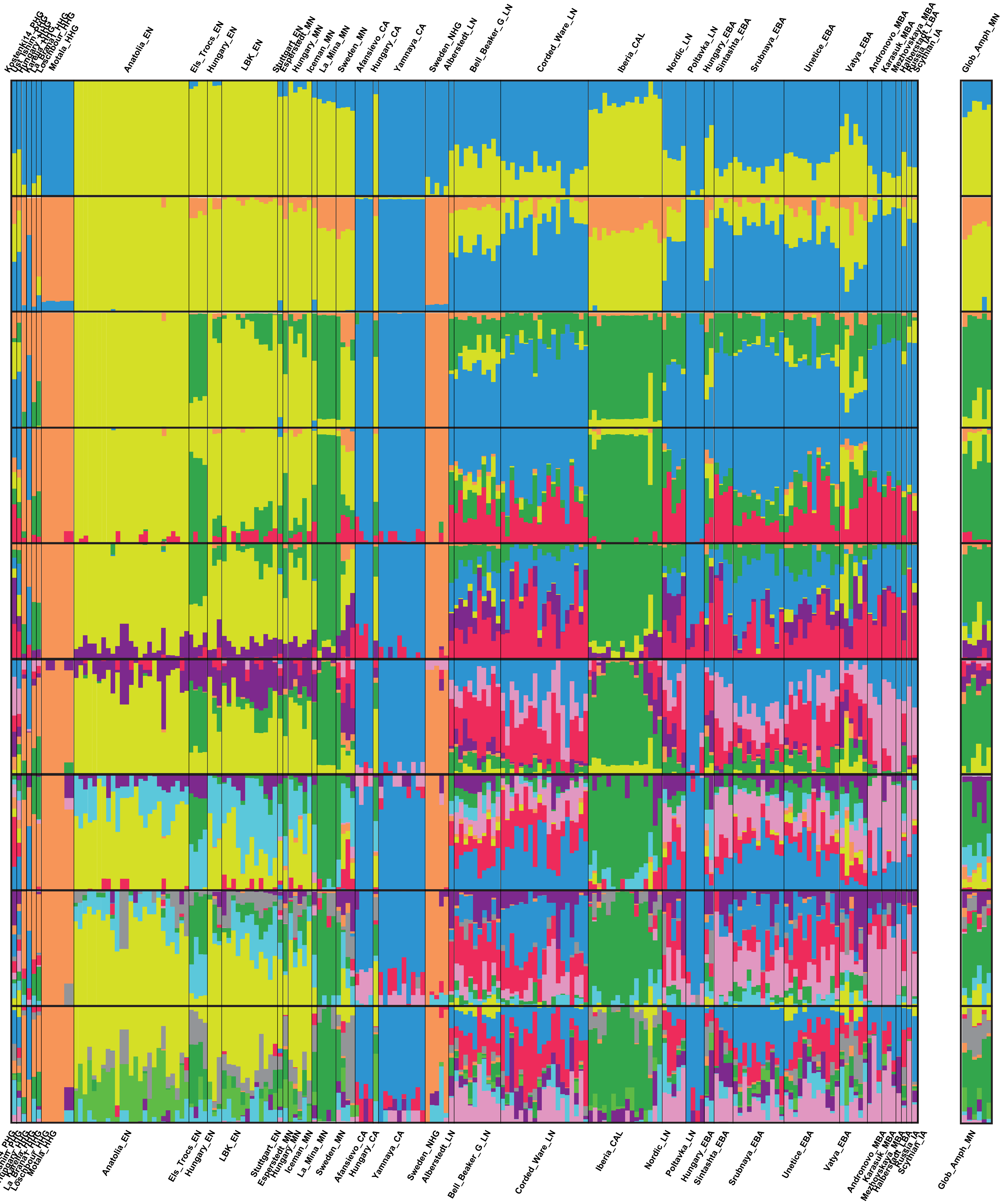

Ancestry proportions inferred from model-based clustering in the ancient individuals. Admixture plots for K=2 to K=10 (Related to fig 2B).The populations are plotted from left to the right according to the following order: Anatolia_EN, Ust_Ishim_PHG, Karasuk_MBA, Corded_Ware_LN, LBK_EN, Iberia_CAL, Motala_HHG, Srubnaya_EBA, Yamnaya_CA, Iceman_MN, Andronovo_MBA, Loschbour_HHG, Unetice_EBA, Stuttgart_EN, Bell_Beaker_G_LN, Afansievo_CA, Poltavka_LN, Nordic_LN, Alberstedt_LN, Els_Trocs_EN, La_Brana1_HHG, Kostenki14_PHG, La_Mina_MN, Karelia_HHG, Mezhovskaya_MBA, Sweden_NHG, Hungary_MN, Halberstadt_LBA, Scythian_IA, Vatya_EBA, Sintashta_EBA, Esperstedt_MN, Hungary_EN, Russia_IA, Hungary_EBA, Hungary_CA, Hungary_HHG, Sweden_MN. The GAC samples of this study are displayed in the last box on the right.

Figure S8 - Admixture analysis based on modern and ancient variation

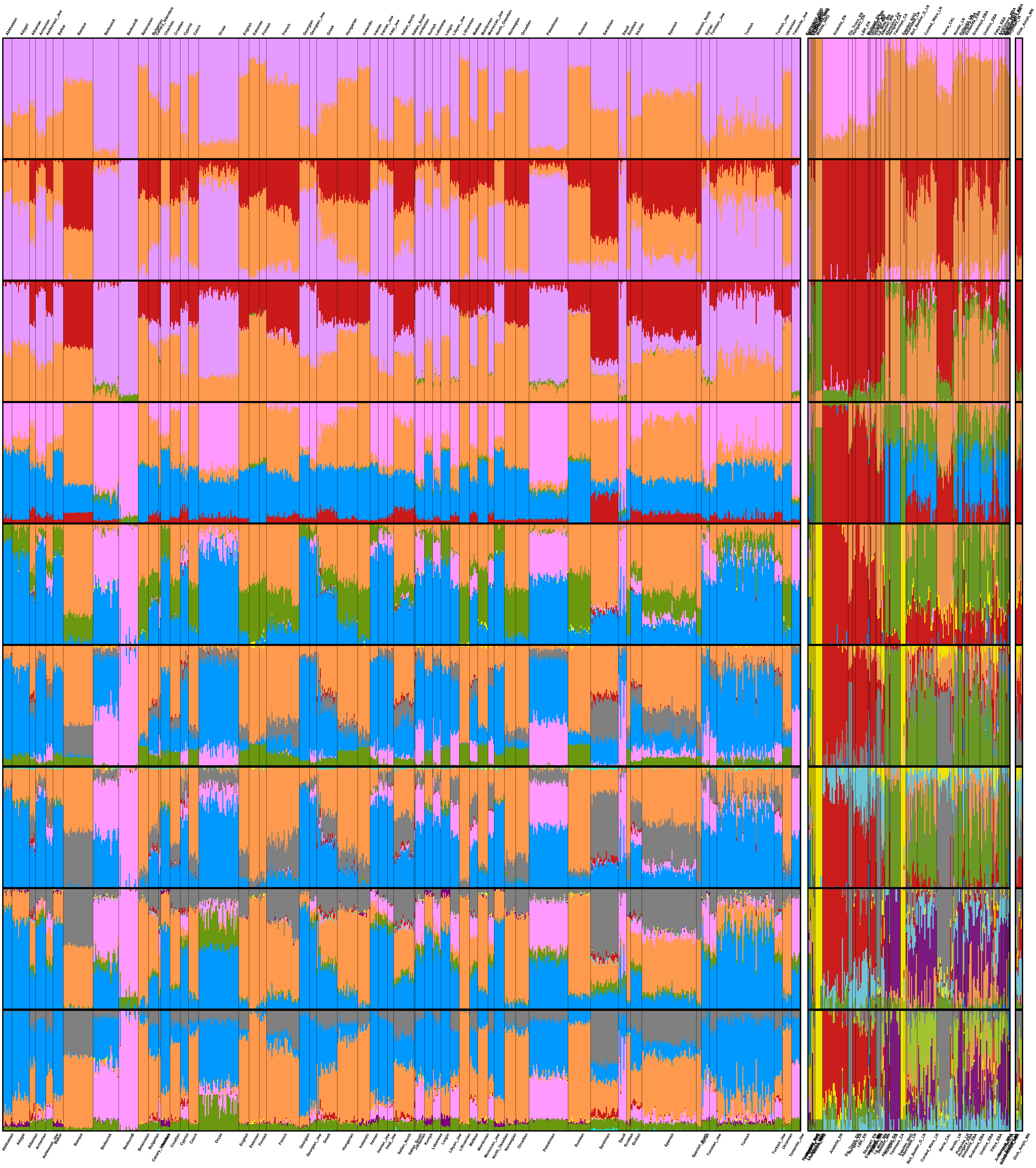

As we did for the dataset including only the ancient samples, we carried out admixture analysis using ADMIXTURE [9], including the 976 individuals (777 modern humans and 199 ancient ones, as detailed in table S2 and S3). The initial set of 101,979 SNPs was pruned for linkage disequilibrium in PLINK [10] using parameters --indep-pairwise 200 25 0.5, resulting in a pruned set of 76,231 SNPs used for analysis. We explored between K= 2 and K=10 using 10 replicates per K with different random seeds. Common signals between the different replicates were identified using the LargeKGreedy mode of CLUMPP [12] and plotted using the software Distruct[13]. Ancient samples are positioned on the right.

From left to right, the populations are as follows: Abkhasian, Adygei, Albanian, Armenian, Ashkenazi_Jew, Balkar, Basque, BedouinA, BedouinB, Belarusian, Bulgarian, Canary_Islanders, Chechen, Croatian, Cypriot, Czech, Druze, English, Estonian, Finnish, French, Georgian, Georgian_Jew, Greek, Hungarian, Icelandic, Iranian, Iranian_Jew, Iraqi_Jew, Italian_North, Italian_South, Jordanian, Kumyk, Lebanese, Lezgin, Libyan_Jew, Lithuanian, Maltese, Mordovian, Moroccan_Jew, North_Ossetian, Norwegian, Orcadian, Palestinian, Russian, Sardinian, Saudi, Scottish, Sicilian, Spanish, Spanish_North, Syrian, Tunisian_Jew, Turkish, Turkish_Jew, Ukrainian, Yemenite_Jew, Anatolia_EN, Ust_Ishim_PHG, Karasuk_MBA, Corded_Ware_LN, LBK_EN, Iberia_CAL, Motala_HHG, Srubnaya_EBA, Yamnaya_CA, Iceman_MN, Andronovo_MBA, Loschbour_HHG, Unetice_EBA, Stuttgart_EN, Bell_Beaker_G_LN, Afansievo_CA, Poltavka_LN, Nordic_LN, Alberstedt_LN, Els_Troc-s_EN, La_Brana1_HHG, Kostenki14_PHG, La_Mina_MN, Karelia_HHG, Mezhovskaya_MBA, Sweden_NHG, Hungary_MN, Halberstadt_LBA, Scythian_IA, Vatya_EBA, Sintashta_EBA, Esperstedt_MN, Hungary_EN, Russia_IA, Hungary_EBA, Hungary_CA, Hungary_HHG, Sweden_MN The GAC samples of this study are displayed in the last box on the right.

Figure S9 TreeMix plot

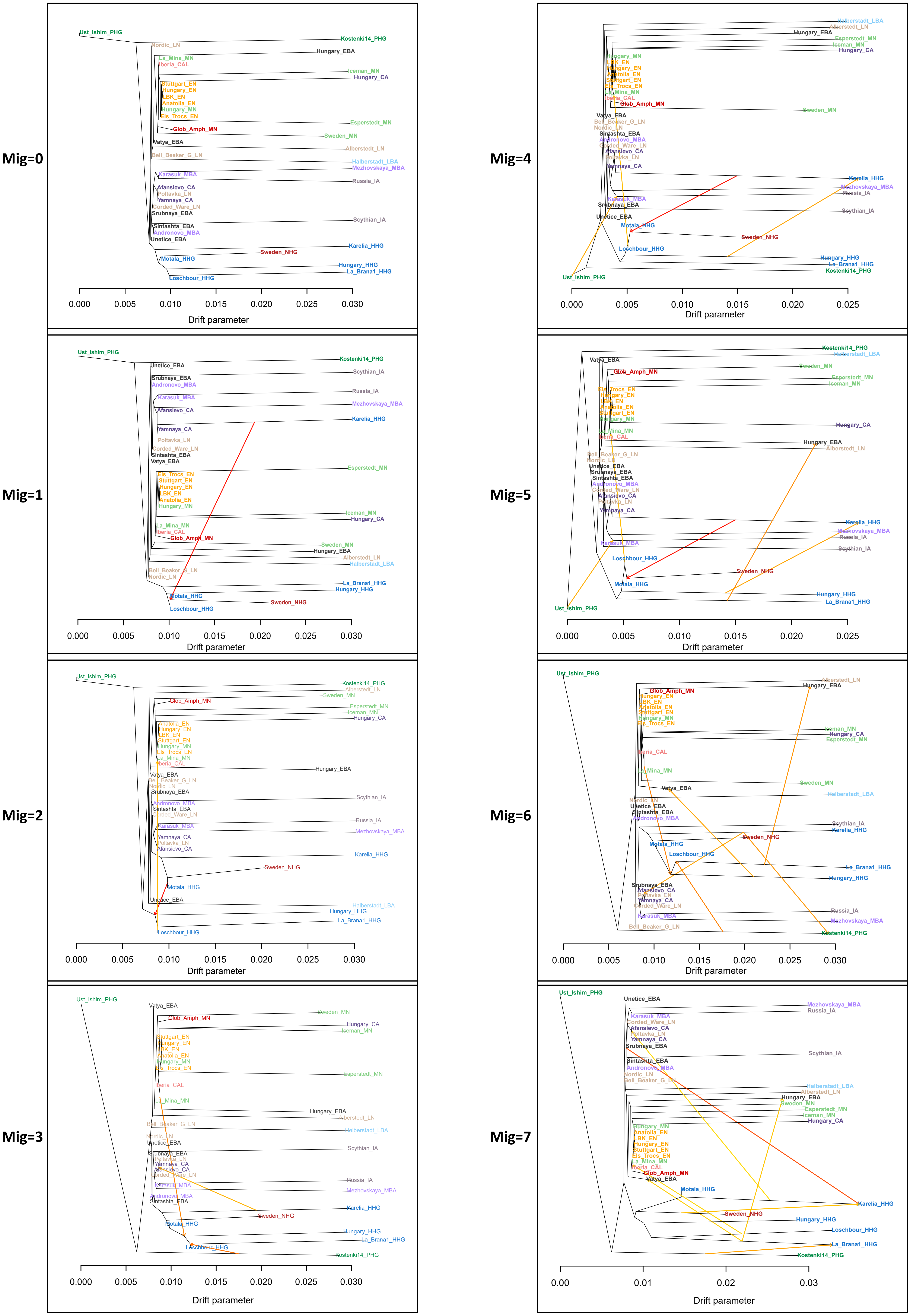

We tested different numbers of migration edges (from 1 to 7) to account for residual covariance not explained by the tree structure.

Figure S10 - EEMS analysis of effective migration rates (m).

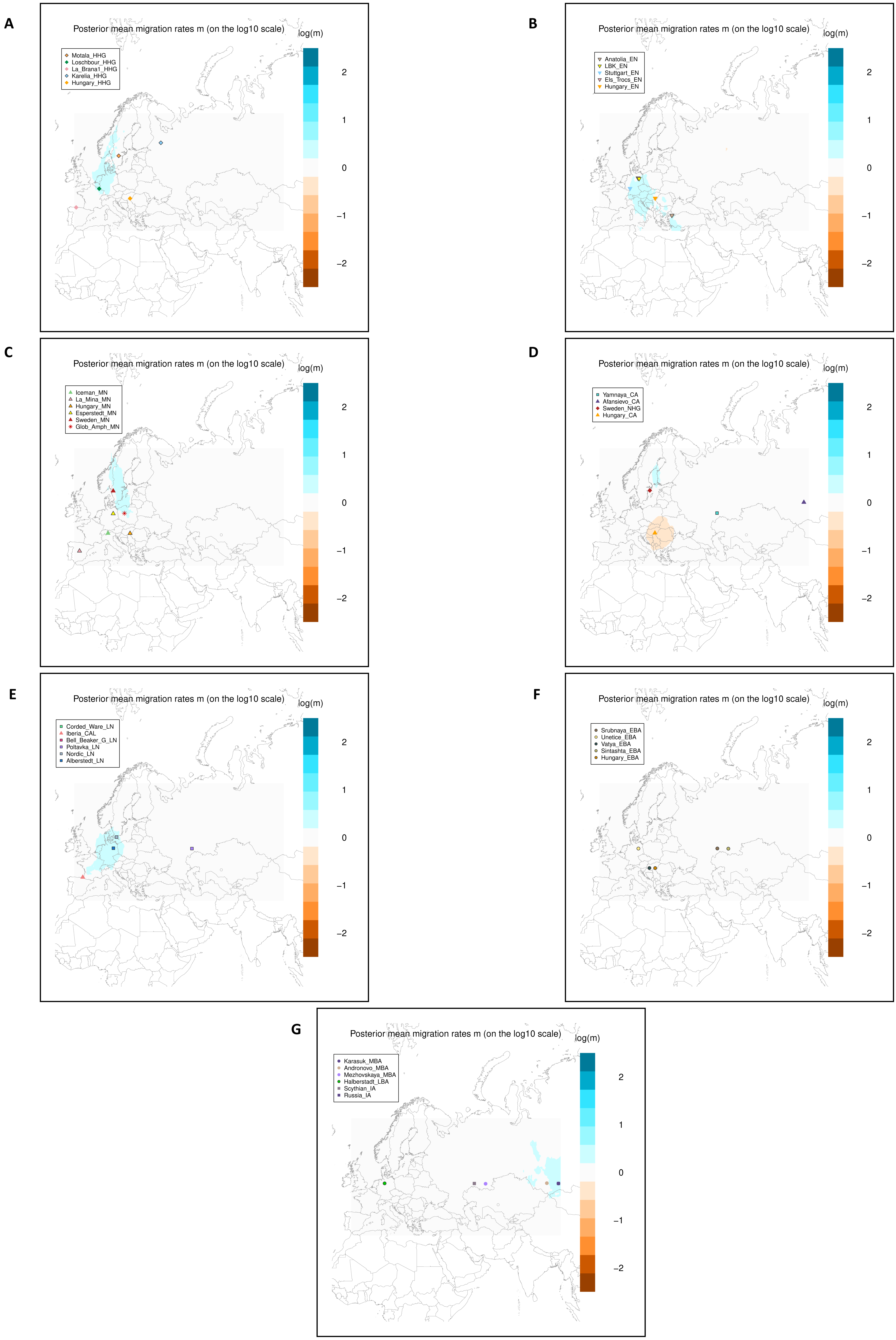

Figure S11- Median-joining network based on nucleotide variation within the dataset of 222 ancient mtDNA sequences.

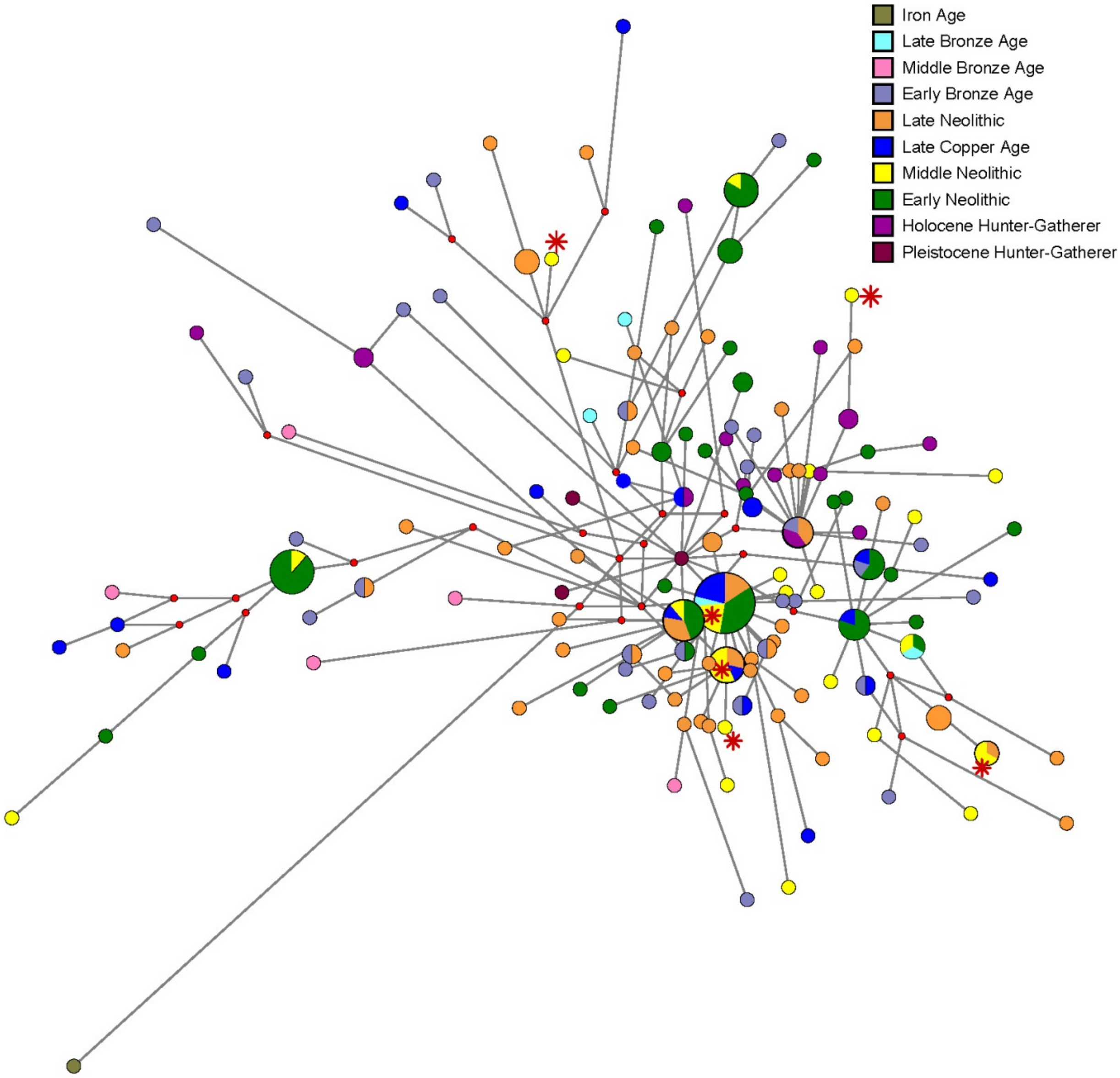

The phylogenetic networks based on nucleotide variation in the 222 mtDNA sequences, were constructed using the Median Joining algorithm [35] implemented in Network 5.0 program (http://www.fluxus-technology.com). The ε value was set to 0 and the transversions were weighted 3x the weight of transitions. Networks were subjected to maximum parsimony post-analysis.

The new GAC samples are highlighted by the red stars. The sequences are coloured according to their time period.

Figure S12- Neighbour Joining phylogenetic tree.

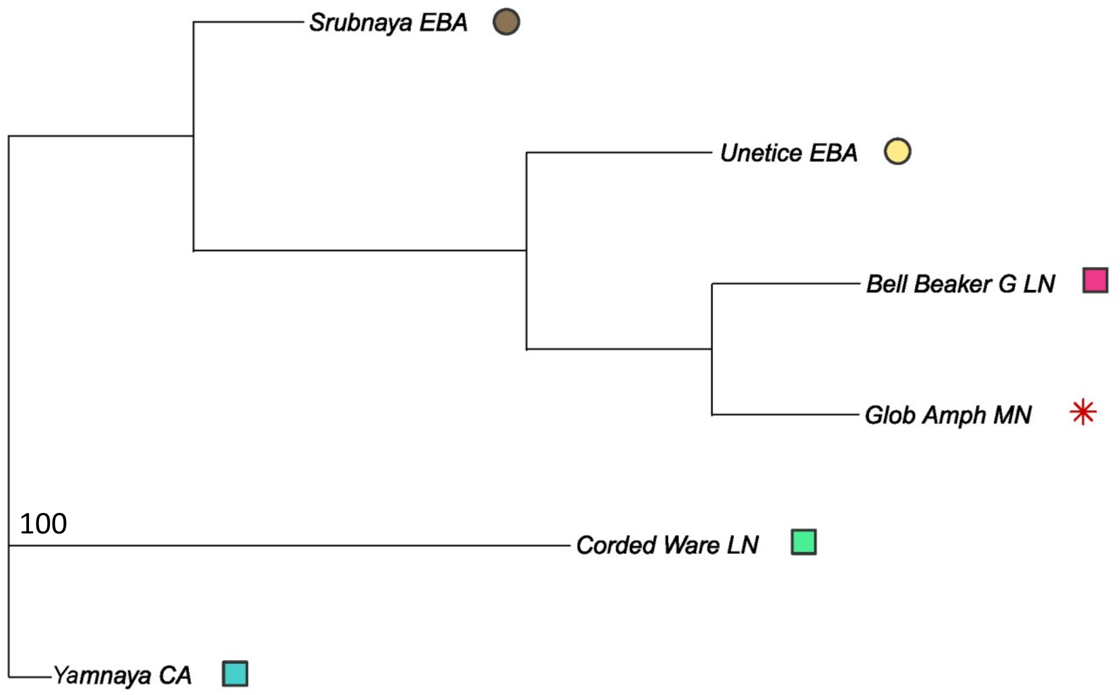

The analysis is based on the mtDNA samples included in the dataset used for the ABC analysis (detail in table S6). Only bootstrap values up to 50 are shown. Symbols and labels refer to the cultures reported in figure 1 (main text).

Figure S13- Median-joining network based on mtDNA nucleotide variation in the dataset used for the ABC analysis.

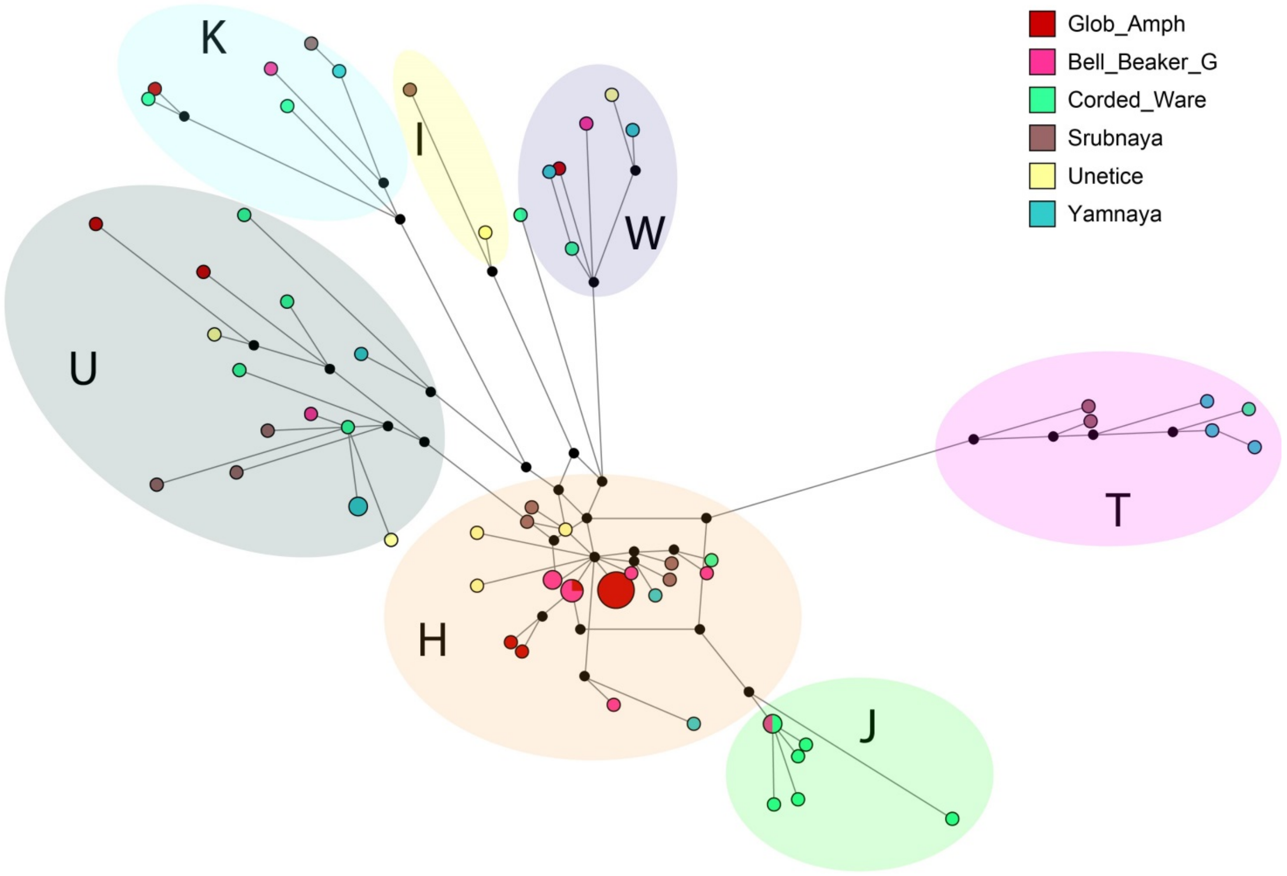

Colours represent the population to which the samples belong, the circles' size is proportional to the number of samples showing that sequence, and the background shading indicates the affiliation of the lineages to the major haplogroups.

Figure S14 - Geographic distribution of mitochondrial haplogroup frequencies.

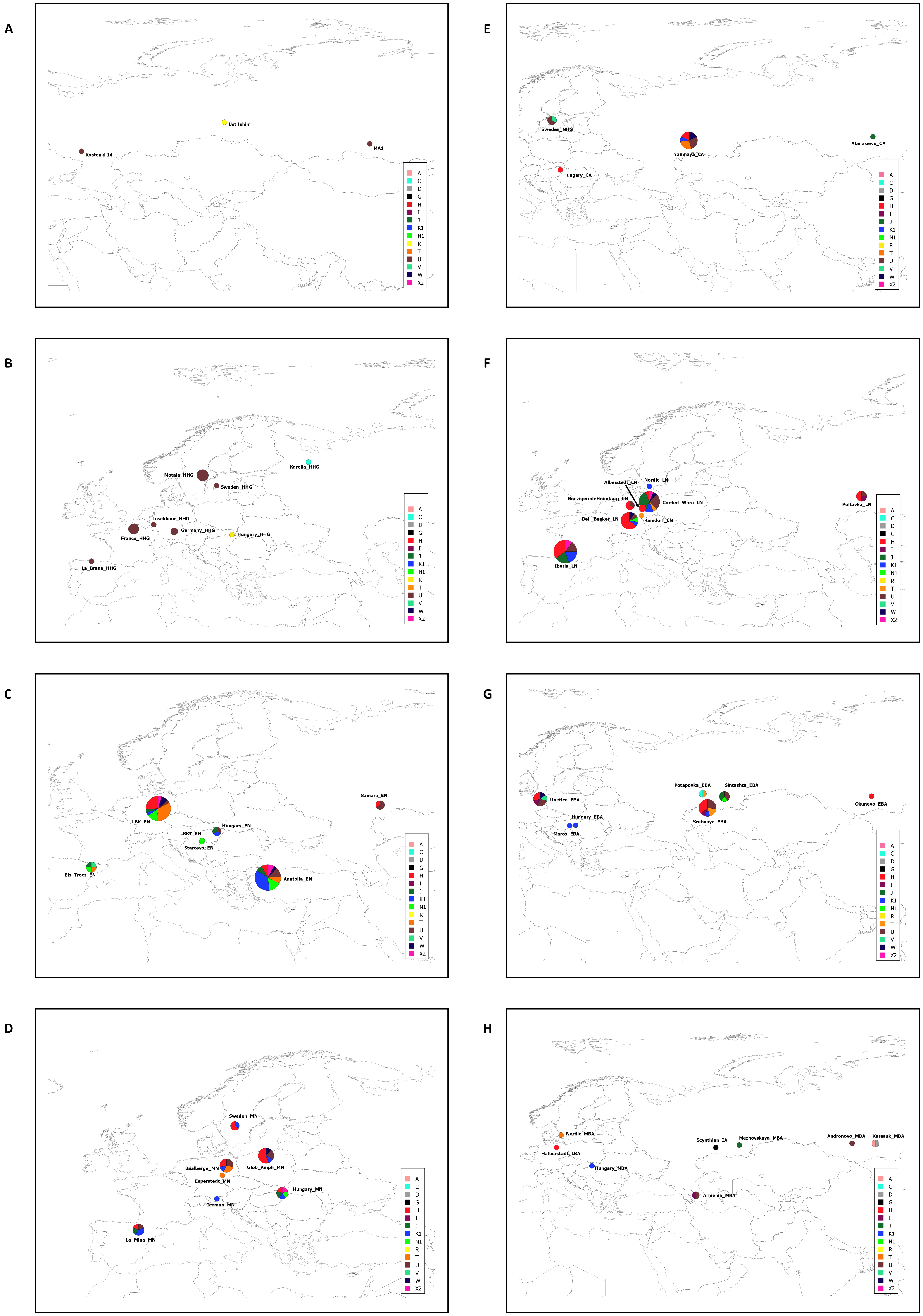

Haplogroup frequencies were obtained for (A) PHG (B) HHG, (C) EN, (D) MN, (E) CA, (F) LN, (G) EBA and (H) LBA+MBA+IA time period. The color assigned to each haplogroup is represented on the lower right part of each plot. Haplogroup frequencies were plotted geographically using QGIS v2.14 [46].

Figure S15 - Linear discriminant analysis (A) and Principal component analysis (B) of the statistics generated by the MIG2 and the MIG2,3 models.

A

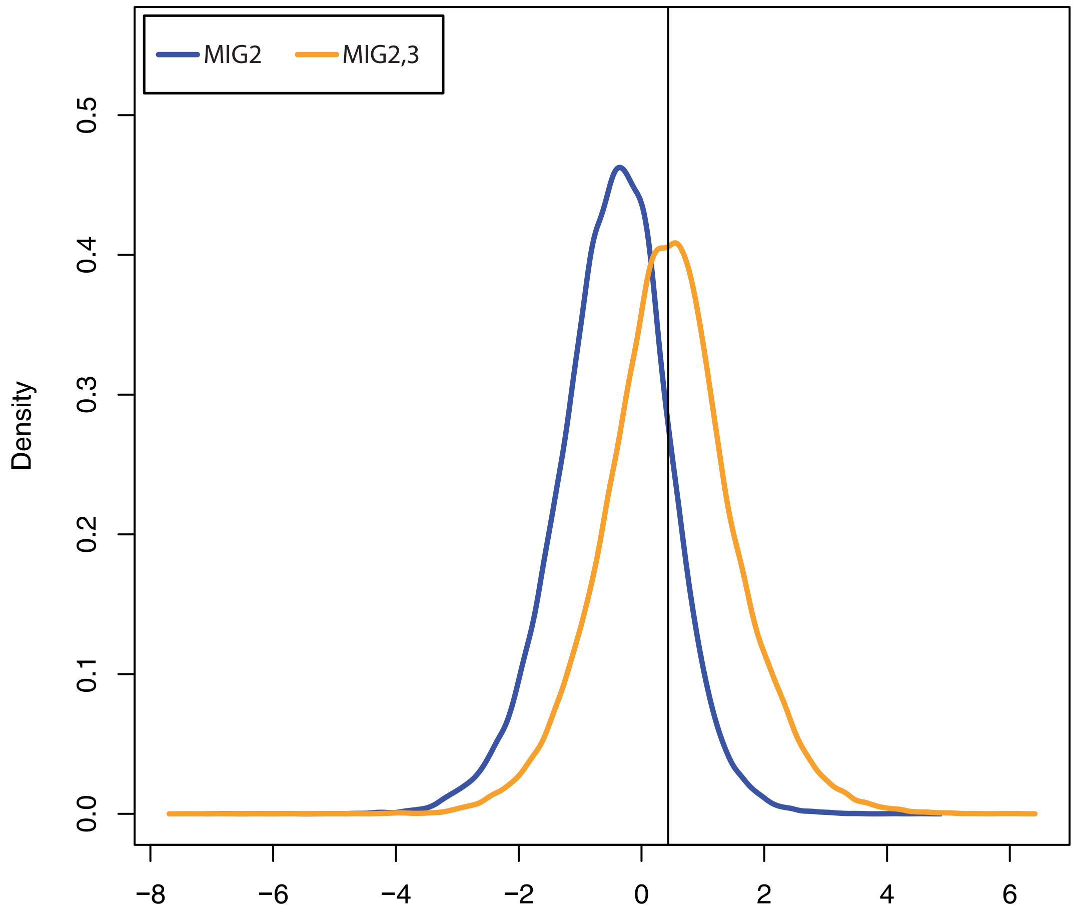

B

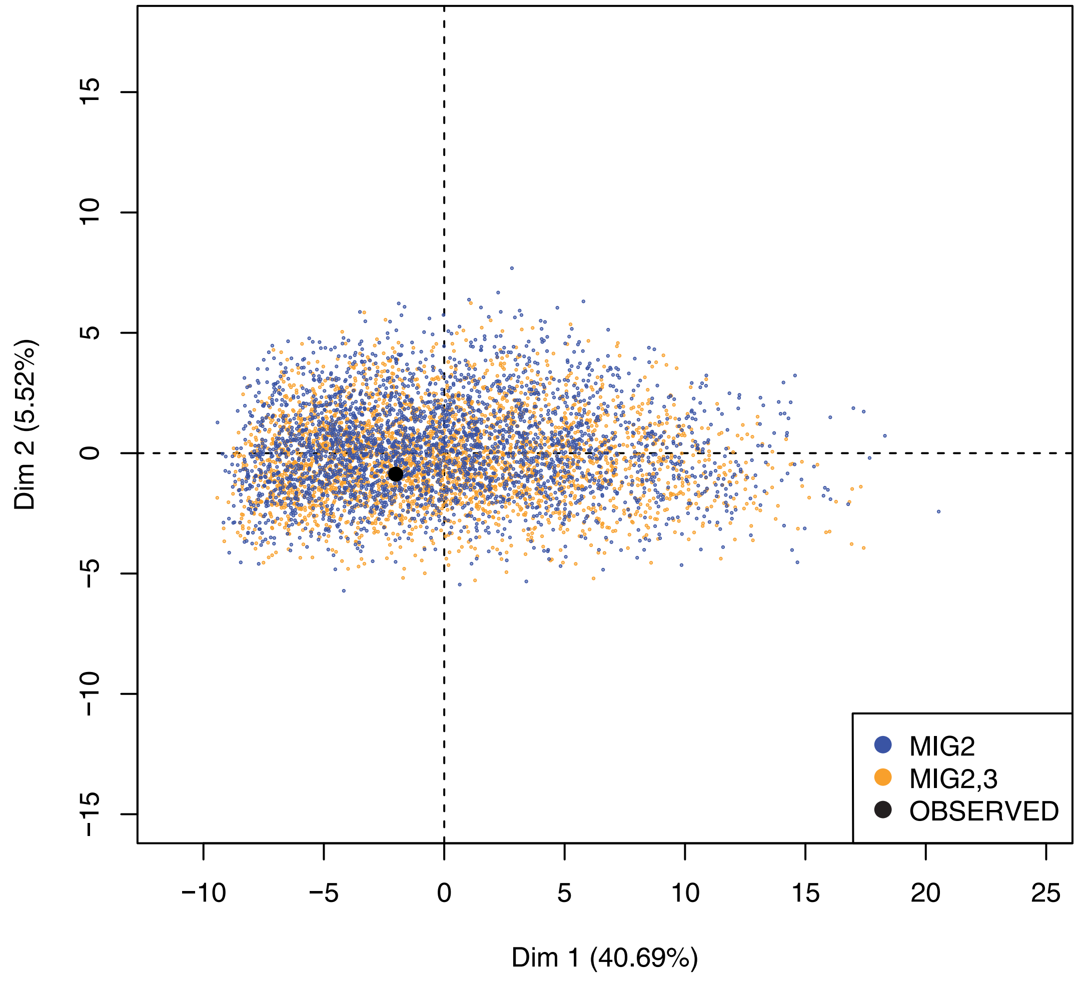

Supplement: Supplementary Figures [file rspb20171540supp2.pdf]
